# Guidelines for preparation and flow cytometry analysis of human nonlymphoid tissue DC

**DOI:** 10.1002/eji.202250325

**Published:** 2024-12-12

**Authors:** Diana Dudziak, Lukas Heger, William W Agace, Joyce Bakker, Tanja D. de Gruijl, Regine J. Dress, Charles‐Antoine Dutertre, Thomas M. Fenton, Marieke F. Fransen, Florent Ginhoux, Oded Heyman, Yael Horev, Florian Hornsteiner, Vinitha Kandiah, Paz Kles, Ruth Lubin, Gabriel Mizraji, Anastasia Prokopi, Or Saar, Sieghart Sopper, Patrizia Stoitzner, Helen Strandt, Martina M Sykora, Elisa C. Toffoli, Christoph H. Tripp, Kim van Pul, Rieneke van de Ven, Asaf Wilensky, Simon Yona, Claudia Zelle‐Rieser

**Affiliations:** ^1^ Institute of Immunology Jena University Hospital Friedrich‐Schiller‐University Jena Germany; ^2^ Laboratory of Dendritic Cell Biology Department of Dermatology University Hospital Erlangen Erlangen Germany; ^3^ Department of Transfusion Medicine and Hemostaseology University Hospital Erlangen Erlangen Germany; ^4^ LEO Foundation Skin Immunology Research Center Department of Immunology and Microbiology University of Copenhagen Copenhagen Denmark; ^5^ Immunology Section Lund University Lund Sweden; ^6^ Institute for Infection and Immunology Cancer Immunology Amsterdam The Netherlands; ^7^ Cancer Center Amsterdam Cancer Immunology Amsterdam The Netherlands; ^8^ Amsterdam UMC location Vrije Universiteit Medical Oncology Amsterdam The Netherlands; ^9^ Institute of Systems Immunology Hamburg Center for Translational Immunology (HCTI) University Medical Center Hamburg‐Eppendorf Hamburg Germany; ^10^ Inserm U1015, Gustave Roussy Villejuif France; ^11^ School of Infection and Immunity University of Glasgow Glasgow UK; ^12^ Department of Pulmonary Diseases Amsterdam UMC location Vrije Universiteit Amsterdam The Netherlands; ^13^ Singapore Immunology Network (SIgN), Agency for Science, Technology and Research Singapore Singapore; ^14^ Department of Immunology and Microbiology Shanghai Institute of Immunology Shanghai Jiao Tong University School of Medicine Shanghai China; ^15^ SingHealth Duke‐NUS Academic Medical Centre Translational Immunology Institute Singapore Singapore; ^16^ INSERM U1015, Gustave Roussy Cancer Campus Villejuif France; ^17^ Department of Periodontology Hadassah Medical Center Faculty of Dental Medicine Hebrew University of Jerusalem Israel; ^18^ Department of Dermatology, Venereology & Allergology Medical University of Innsbruck Innsbruck Austria; ^19^ Faculty of Dental Medicine The Institute of Biomedical and Oral Research Hebrew University of Jerusalem Israel; ^20^ Internal Medicine V, Hematology and Oncology Medical University of Innsbruck Innsbruck Austria; ^21^ Tyrolean Cancer Research Center Innsbruck Austria; ^22^ Department of Otolaryngology, Head and Neck Surgery Amsterdam UMC location Vrije Universiteit Amsterdam The Netherlands

**Keywords:** dendritic cells, nonlymphoid tissues, tumor, tumor‐draining lymph node, flow cytometry

## Abstract

This article is part of the Dendritic Cell Guidelines article series, which provides a collection of state‐of‐the‐art protocols for the preparation, phenotype analysis by flow cytometry, generation, fluorescence microscopy, and functional characterization of mouse and human dendritic cells (DC) from lymphoid organs, and various nonlymphoid tissues. Within this article, detailed protocols are presented that allow for the generation of single‐cell suspensions from human nonlymphoid tissues including lung, skin, gingiva, intestine as well as from tumors and tumor‐draining lymph nodes with a subsequent analysis of dendritic cells by flow cytometry. Further, prepared single‐cell suspensions can be subjected to other applications including cellular enrichment procedures, RNA sequencing, functional assays, etc. While all protocols were written by experienced scientists who routinely use them in their work, this article was also peer‐reviewed by leading experts and approved by all co‐authors, making it an essential resource for basic and clinical DC immunologists.

## Graphical abstract

We here provide detailed protocols for isolating single cell suspension from human nonlymphoid tissues, such as gingiva, lung, intestine, skin, and different tumor tissues as well as tumor‐draining lymph nodes. Further, we provide gating strategies for flow cytometric analysis of human DC subpopulations in diverse nonlymphoid tissues.

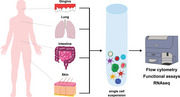

## 1 Preparation of human single‐cell suspensions of the lung

### 1.1 Introduction

**Figure 1:**
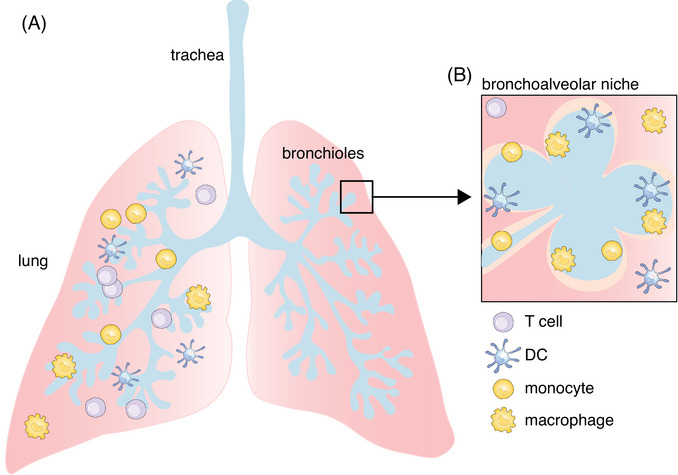
Schematic overview of the lung. (A) Schematic view of the lung showing the trachea and bronchioles (light blue) and lung (pink) tissue. Immune cell populations, such as dendritic cells, monocytes, macrophages, and tissue‐resident T cells can be spread out across the entire lung and are partially found in the airways as well. (B) Schematic view of the bronchoalveolar niche, depicting the alveolar and interstitial niches. DC and monocytes can be found spread across the entire bronchoalveolar niche, while alveolar and interstitial macrophages are refined to their specific niche.

The lung is one of the main barrier tissues shielding the host from the environment and thus is a tissue highly vulnerable to allergens, polluting agents, or infective pathogens. In general, the respiratory tract can be divided into the upper and the lower airways with the upper airways consisting of the nasal tract, pharynx, and larynx. The lower airways, forming the lung, can be divided into bronchioles and alveoli which are in close contact with a network of capillaries and the interface of active oxygen and carbon dioxide exchange [1]. Immune cells, including dendritic cells (DC), can be found across all parts of the respiratory tract, specifically the lung (Fig. 1), as well as in the lung‐draining lymph nodes [2, 3]. These cells are critical for maintaining lung tissue homeostasis, host resistance to infections, allergens cancer, and other pulmonary diseases. This has become very obvious recently in patients with COVID‐19, a disease caused by infection with SARS‐CoV2, where a massive dysregulation of monocytes and macrophages but also DC responses and functions results in severe lung damage and fibrosis [4, 5, 6].

To study lung immune cells, several methods of sampling the human respiratory tract and lung are possible. As it is tricky to obtain lung tissue from healthy patients, one of the most common methods is to use bronchioalveolar lavage fluid (BALF) for immune cell analyses. Therefore, a bronchoscopy with a bronchial wash can be performed to obtain lavage fluid. This wash or lavage fluid mostly contains cells lining the airways or mucosal surface of the lung, rather than tissue‐embedded cells. Sequential lavages are possible and allow further separate bronchial (bronchial wash) and alveolar (BALF) samples [1]. While this method results in minimal manipulation of immune cells prior to flow cytometric analyses, it only provides a limited overview of the lung immune compartment, and DC generally are less frequent in the BALF than in lung tissue. To obtain the most immune cells from the lung, including resident cells, or to study lung architecture and spatial distribution of immune cells across the alveolar or interstitial spaces whole tissue sections are necessary. Most often these come from surgical resection of lung tissue during tumor removal, tissue removal during other acute or chronic lung diseases (such as chronic obstructive pulmonary disease), or from postmortem biopsies and organ donors. During the removal of the tumor tissue surrounding healthy tissue will be sampled as well, which then can be analyzed as healthy control tissue.

In the below protocol, we will focus on how to prepare single‐cell suspensions, with a focus on the isolation of DC (and other mononuclear phagocytes), from human lung tissue.

### 1.2 Materials

#### 1.2.1 Reagents

A complete list of reagents is provided in Table 1.

**Table 1:** Reagents, enzymes, chemicals, and solutions.

Reagent	Manufacturer	Ordering number
Enzymes		
Collagenase IV	Sigma	C5138
DNAse I	Roche	10104159001
Chemicals and solutions		
Dulbecco's phosphate‐buffered saline (PBS)	Sigma	D8537
Ficoll‐plaque	GE Healthcare	17‐1440‐02
Fetal bovine serum (FBS)	Serana	0261S‐FBS‐SA‐015
1× RBC lysis buffer	eBioscience	00‐4333‐57
RPMI 1640	HyClone	SH30255.01
Trypan blue stain (0.4%)	Invitrogen	T10282

#### 1.2.2 Equipment

The necessary equipment are listed in Table 2.

**Table 2:** Necessary equipment.

Equipment	Company	Purpose
Centrifuge “Allegra X‐15R”	Beckman‐Coulter	Centrifugation of 50 mL tubes, 15 mL tubes, and V‐bottom plates
Countess 3 Cell counter	Invitrogen	Cell counting
Countess Cell Counting Chamber Slides (#C10312)	Invitrogen	Cell counting
Incubator “HERAEUS BBD6220”	Thermo Scientific	Cabinet‐style incubator with 5% CO_2_ and 37°C for digestion of lung tissue
Sterile bench	–	Performance of all aseptic procedures
Scissors, fine, autoclaved	–	Scissor for mincing lung tissue
Six‐well plates (#140675)	Thermo Scientific	Storage and digestion of lungs
96‐well V‐bottom plate (651 180)	Greiner bio‐one	Sample preparation for flow cytometry
2 mL microcentrifuge tubes (#0030120094)	Eppendorf	Storage and mincing of lung tissue for digest
5 mL polystyrene flow cytometry tubes (#352008)	Falcon	Staining of samples for flow cytometry
50 mL conical tubes (#352070)	Falcon	Centrifugation of cell suspensions
Serological pipettes (e.g. #GN606180)	Greiner	Pipetting
Falcon 70 µm Cell Strainer, for 50 mL tubes (#352350)	Corning	Filtration of lung tissue, generation of single‐cell suspensions

### 1.3 Step‐by‐step sample preparation

#### 1.3.1 Preparation of stocks and solutions

##### 1.3.1.1 DNAse I

Prepare deoxyribonuclease I (DNAse I) solution under sterile conditions. Reconstitute lyophilized DNAse I in sterile and double‐distilled water to reach a concentration of 10 mg/mL. Prepare aliquots (e.g. enough DNAse I for 50 mL buffer) and store them at −20°C. Aliquots are meant for single use and avoid freeze‐thaw cycles.

##### 1.3.1.2 Collagenase IV

Prepare collagenase IV solution under sterile conditions. Dissolve collagenase IV in sterile, double‐distilled water to reach a concentration of 10 mg/mL. Prepare aliquots (e.g. enough Collagenase IV for 50 mL buffer) and store them at ‐20°C. Aliquots are meant for single use and avoiding freeze‐thaw cycles.

##### 1.3.1.3 Fetal bovine serum

Thaw FBS in a water bath, at 37°C. Incubate thawed FBS for 30 min at 56°C in a water bath to inactivate. Working in a sterile bench, filter inactivated FBS through a sterile 0.22 µm membrane (Corning #431118) into a sterile storage bottle (Corning #430518) and aliquot into 50 mL tubes. Store aliquots at −20°C.

##### 1.3.1.4 Digestion buffer for isolation of DC from the lung tissue

Add 0.2 mg/mL Collagenase IV + 0.05 mg/mL of DNase I in RPMI 1640 containing 10% FBS.

#### 1.3.2 Preparation of single‐cell suspensions from lung tissue

1. Prepare 20 mL of digestion buffer.
2. Allow Ficoll‐Paque and 1× RBC lysis buffer to equilibrate to room temperature (RT).
3. Transfer the lung sample into a 2 mL microcentrifuge tube containing 0.5 mL of the digestion buffer (note: biopsy size used here was around 1–2 cm, per 2 mL tube).
4. Using sterile scissors mince the lung tissue into tiny pieces (approx. 1–2 mm).
5. Transfer the minced lung and digestion buffer mixture into one well of a six‐well plate and add another 4 mL of digestion buffer (per well).
6. Incubate for 1 h at 37°C.
7. At the end of incubation, gently pipette the mixture up and down about 6–8 times using a 10 mL sterile, disposable serological pipette, to disrupt the remaining tissue and achieve a single‐cell suspension.
8. Transfer the lung suspension over a 70 µm cell strainer into a 50 mL conical tube.
9. Rinse the well of the six‐well plate with 1 mL PBS* and add this to the cell suspension in the 50 mL conical tube (via 70 µm cell strainer; to ensure minimum cell loss).
10. Adjust the volume of the lung suspension to a total of 50 mL, with PBS*.
11. Centrifuge at 365 × *g* for 5 min, at 25°C.
12. Aspirate supernatant and resuspend the cell pellet in 40 mL of PBS* to achieve a thorough dilution of the lung cell suspension.
13. Aliquot 10 mL of RT Ficoll‐Paque into a new clean 50 mL conical tube.
14. Carefully transfer the 40 mL of the diluted lung cell suspension as a top layer onto the 10 mL of prewarmed (RT) Ficoll‐Paque.
15. Centrifuge at 1800 × *g* for 25 min, at RT. Critical: set centrifuge to acceleration = 0–1 and brake = 0–1.
16. Collect the layer of mononuclear cells, which is found at the plasma (PBS)‐Ficoll‐Paque interface, and transfer it into a new clean 50 mL conical tube.
17. Top up with PBS* to a final volume of 50 mL.
18. Centrifuge at 365 × *g* for 5 min, at 4°C. Critical: Set the centrifuge to maximum acceleration and maximum brake.
19. Aspirate the supernatant.
20. Re‐suspend the pellet in 1 mL of 1X RBC lysis buffer and incubate for 5 min at RT.
21. Top up with PBS* to a final volume of 50 mL.
22. Centrifuge at 365 × *g* for 5 min, at 4°C.
23. Aspirate the supernatant and resuspend the cell pellet (which contains the DC) in 1 mL of PBS*.
24. Count cells (e.g. mix 10 µL of trypan blue with 10 µL of cell suspension, in a 96‐well plate well, and add 10 µL to the counting chamber to count) and proceed with the flow cytometry staining protocol.

*Note: PBS can be used either at RT or cold/at 4°C. In general, cells are more stable and show improved viability if kept at colder temperatures during processing but it may be of advantage to keep PBS at RT for the Ficoll‐Paque gradient since these steps are performed at RT. However, we have not noticed a significant difference in outcome based on the PBS temperature for the Ficoll‐Paque gradient.

### 1.4 Data analysis

Examples of flow cytometry data analysis of human lung DC subsets using the described single cell preparation are covered in detail in the section **2** **Flow cytometric analysis of the human lung DC compartment**.

### 1.5 Pitfalls

Most lung biopsies will contain blood, as it is difficult to perform perfusion of the entire lung during surgery. While red blood cells can be removed by performing RBC lysis (as described in steps 20–23), the remaining immune cell populations analyzed may contain a mixture of circulating and resident cells.

Depending on the source of the lung biopsy, for example, fresh vs. postmortem, healthy vs. diseased tissue, the cell viability and recovery of cell numbers may vary. To obtain the best yields work quickly and keep samples on ice whenever possible. Additionally, using cold (4°C) reagents from step 17 on (after density gradient centrifugation) could be beneficial as well.

### 1.6 Top tricks

The tissue digestion buffer works best when prepared freshly before each use. Upon preparation keep the digestion buffer at RT for short‐term use for optimal enzyme activity (store on ice, if a few hours are between preparation and use).

It has been reported that FBS may decrease collagenase activity, thus digestion buffers may be prepared with or without FBS or lower concentrations (5% FBS instead of 10%), depending on the researcher's preference. For this protocol, we have not noticed a difference in outcome with or without the addition of FBS.

After the digest, make sure to rinse the well in which digestion was performed to keep as many cells as possible and reduce cell loss during preparation. Similarly, aspirating supernatants will reduce cell loss compared with simply discarding supernatants.

Similarly, 1× RBC lysis buffer works best when prewarmed to RT before use.

Fresh biopsies usually lead to better results for flow cytometry, or single‐cell RNA‐sequencing analyses, than frozen‐thawed samples. If possible, use samples fresh. If this is not possible, try to prepare repetitive lung samples always in the same way, for example, frozen‐thawed only, instead of mixing fresh/frozen‐thawed, for the most comparable results.

Work quickly and keep samples on ice whenever possible for best outcome, regarding cell viability, numbers, and surface marker phenotypes.

## 2 Flow cytometric analysis of the human lung DC compartment

### 2.1 Introduction

DC are innate immune cells that orchestrate innate and adaptive immune responses and are crucial for tissue homeostasis, response to inflammation, and during infection with various pathogens. The high heterogeneity among DC subsets is also reflected by their diverse functions, especially in tissues as exposed to the environment as the lung. Here, they can initiate maintaining and balancing processes of immune tolerance versus immunity to tissue and environmental antigens.

DC were first described by Steinman and Cohn [7] in the 1970s, who identified a novel cell type with a stellate morphology, thus naming them DC. Within the respiratory tract, DC were described in the nasal mucosa, and epithelium, the submucosa of airways, the lung parenchyma, and within alveolar surfaces — basically covering the entire tract. It was shown, that their half‐life within the airways is rather short, with a replacement of DC about every two days [1, 3]. Here, DC are situated perfectly to sample foreign antigens arriving through the airways into the lungs. Upon detection of foreign antigens, DC migrate to the lung draining lymph nodes to present these antigens to T cells there, inducing activation and proliferation of T cells and protective immune responses. This ability of antigen presentation is one of the key features of DC, which are often simply described as “professional antigen‐presenting cells” however it is not unique to them [8]. The capacity of DC (and others) to efficiently present antigens frequently is measured using mixed lymphocyte reaction assays, where antigen‐experienced DC are co‐cultured with T cells whose proliferation rate and cytokine response then is measured.

Human DC can be divided into conventional DC (cDC), which are further characterized as either CLEC9A^+^XCR1^+^CADM1^+^CD141^+^ cDC1 or CD1c^+^ cDC2, and CD123^+^ plasmacytoid DC (pDC) [2]. With the improvement of analyses methods, including single‐cell sequencing and multiparameter flow cytometric phenotyping additional human DC subsets have been described, including mregDC, CD14^+^ DC3 and pre‐DC, which are progenitors of cDC found across all tissues [9, 10]. While cDC1 is capable of cross‐presentation of antigens and is specialized in responding to intracellular pathogens, thus initiating T_H_1 responses, cDC2 may be more specialized to extracellular pathogens and initiate T_H_2 or T_H_17 responses [11]. On the other hand, pDC, whose origin and functions suggest they actually may not be DC at all but some sort of innate lymphoid cell type [12], are poorly equipped for antigen presentation. Rather, these cells are “professional” type I interferon‐producing cells. They constitutively express the transcription factor IRF7, which enables them to secrete large amounts of type I interferon immediately in response to, for example, viral infection [13].

In order to understand how DC function and to study their diverse roles during homeostasis, inflammation, cancer, autoimmunity, infections, and other diseases, a common protocol and proper analysis of the DC compartment is essential. Employing a combination of methods, including phenotypical, transcriptomic, and functional analyses, can help to correctly define different DC across tissues and states. Here, we provide a protocol for the flow cytometric analysis of human lung DC populations.

### 2.2 Materials

#### 2.2.1 Reagents

A list of chemicals and solutions for flow cytometry staining is provided in Table 3, and a list of antibodies to stain for DC in the human lung is provided in Table 4.

**Table 3:** Reagents, enzymes, chemicals, and solutions.

Reagent	Manufacturer	Ordering number
Chemicals and solutions		
CompBeads Anti‐Mouse Igk	BD Bioscience	552843
Dulbecco's PBS without calcium and magnesium	Sigma	D8537
EDTA	Promega	V4231
Fetal bovine serum (FBS)	Serana	0261S‐FBS‐SA‐015
LIVE/DEAD fixable blue dead cell stain kit	Life Technologies	L23105
Sodium Azide	Sigma‐Aldrich	13412

**Table 4:** Antibodies.

Antibodies	Fluorochrome	Isotype	Clone	Manufacturer	Ordering number	Dilution
CADM1	Purified	Chicken IgY	3E1	MBL	CM004‐3	1:100
CD1c	Super Bright 436	Mouse IgG1	L161	eBioscience	62‐0015‐42	1:100
CD3ε	BV605	Mouse IgG1	UCHT1	BioLegend	300460	1:200
CD5	APC/R700	Mouse IgG1	UCHT2	BD Biosciences	565121	1:100
CD14	Spark Blue 550	Mouse IgG1	M5E2	BioLegend	367147	1:100
CD16	BV650	Mouse IgG1	3G8	BioLegend	302018	1:100
CD19	BV650	Mouse IgG1	HIB19	BioLegend	302238	1:200
CD20	BV650	Mouse IgG2b	2H7	BioLegend	302336	1:200
CD45	PerCP	Mouse IgG1	HI30	eBioscience	45‐0459‐73	1:200
CD123	PE/Dazzle 594	Mouse IgG1	S18016C	BioLegend	396605	1:100
CD141	BV421	Mouse IgG1	1A4	BD Biosciences	565321	1:100
CD169	BUV661	Mouse IgG1	7‐239	BD Biosciences	750363	1:100
HLA‐DR	APC/Fire810	Mouse IgG2a	L243	BioLegend	307674	1:100
anti‐Chicken IgY	Alexa Fluor 647	Donkey Fab'2	N/A	Jackson Immuno‐research	703‐606‐155	1:200
CD88	APC/Fire750	Mouse IgG2a	S5/1	BioLegend	344315	1:100

#### 2.2.2 Equipment

The necessary equipment are listed in Table 5.

**Table 5:** Necessary equipment.

Equipment	Company	Purpose
Centrifuge “Allegra X‐15R”	Beckman–Coulter	Centrifugation of 50 mL tubes, 15 mL tubes, and V‐bottom plates
Sterile bench	–	Performance of all aseptic procedures
96‐well V‐bottom plate (#GN651180)	Greiner	Sample preparation for flow cytometry
2 mL microcentrifuge tubes (#0030120094)	Eppendorf	Storage and mincing of lung tissue for digest
5 mL polystyrene flow cytometry tubes (#352008)	Falcon	Staining of samples for flow cytometry
50 mL conical tubes (#352070)	Falcon	Centrifugation of cell suspensions
Serological pipettes (e.g. #GN606180)	Greiner	Pipetting
Falcon 70 µm cell strainer, for 50 mL tubes (#352350)	Corning	Filtration of lung tissue, generation of single‐cell suspensions

### 2.3 Step‐by‐step sample preparation

#### 2.3.1 Preparation of stocks and solutions

##### 2.3.1.1 Fetal bovine serum

Thaw FBS in a water bath, at 37°C. Incubate thawed FBS for 30 min at 56°C in a water bath to inactivate. Working in a sterile bench, filter inactivated FBS through a sterile 0.22 µm membrane (Corning #431118) into a sterile storage bottle (Corning #430518) and aliquot into 50 mL tubes. Store aliquots at −20°C.

##### 2.3.1.2 Flow cytometry buffer (FACS buffer)

Add 2% FBS + 2 mM EDTA (solved in PBS) + 0.05% sodium azide in 1× PBS. Store at 4°C for long‐term storage or on ice for immediate use and during the experiment.

#### 2.3.2 Flow cytometry staining of human lung DC

In the section **1** **Preparation of human single‐cell suspensions of the lung**, we described how to prepare single‐cell suspensions from human lung tissue. Single‐cell suspensions then are transferred either to a 96‐well V‐bottom plate or 5 mL polystyrene flow cytometry tubes to perform antibody staining for flow cytometric analysis.

1. Prepare lung single‐cell suspensions as described in the section **1** **Preparation of human single‐cell suspensions of the lung**.
2. Aliquot cells into either a 5 mL polystyrene FACS tubes or a V‐shaped 96‐well plate (nonculture‐treated). The following protocol is used for staining DC, optimal 1–5 × 10^6^ cells/5 mL polystyrene FACS tube for staining. Recommended: Keep extra unstained cells for compensation controls/flow cytometer setup.
3. Centrifuge at 650 × *g* for 2 min, at 4°C.
4. Aspirate the supernatant.
5. Resuspend the cell pellet in 1 mL of PBS containing LIVE/DEAD Fixable Blue Dead Cell Stain (1:1000) and incubate for 20 min, at 4°C in the dark. Without washing, directly add human AB serum or FBS to the cell suspension (5% of serum in 1 mL of cell suspension). Incubate for 15 min, at 4°C in the dark, in order to block FC receptors on the immune cells and neutralize free Live/Dead molecules that bind protein N‐terminal amines.
6. During this incubation time prepare an antibody cocktail (Table 6). Add all primary antibodies into a tube containing FACS buffer, according to the dilution stated in Table 6, to a final volume of 50 µL.
7. Add 2 mL of FACS buffer (if using a 96‐well plate add 200 µL) and centrifuge at 650 × *g* for 2 min, at 4°C.
8. Aspirate the supernatant.
9. Resuspend the cell pellet in 50 µL of antibody cocktail. Incubate for 30 min, at 4°C in the dark. Recommended: During this incubation period prepare unstained, single‐stained, and live/dead control for the flow cytometer setup. For unstained and Live/Dead controls use cells (∼200 µL of cell suspension for each). Stain live/dead as before. For single stains of each antibody, use either beads or cells. Prepare on the tube with beads or cells for each antibody used and stain in FACS buffer in the same concentration/dilution used for preparing the antibody cocktail before.
10. Add 2 mL of FACS buffer (if using a 96‐well plate add 200 µL), and centrifuge at 650 × *g* for 2 min, at 4°C.
11. Aspirate the supernatant.
12. Optional: If you chose to include CADM1 into your staining (to target cDC1) and since a purified antibody is used to stain for CADM1 on DC, you will need to perform an additional staining step (otherwise proceed with step 13.):
  - Resuspend the cell pellet in 50 µL of FACS buffer containing anti‐Chicken‐IgY‐Alexa‐Fluor 647 (to target CADM1). Incubate for 15 min, at 4°C. Then add 2 mL of FACS buffer (if using a 96‐well plate add 200 µL) and centrifuge at 650 × *g* for 2 min, at 4°C.
  - Aspirate the supernatant.
13. Resuspend the cell pellet in 200–400 µL of FACS buffer, filter through a 70 µm cell strainer into a new (clean) 5 mL polystyrene FACS tube, and analyze using a suitable flow cytometer.

**Table 6:** Antibody cocktail for lung DC staining.

Antibodies	Fluorochrome	Isotype	Clone	Manufacturer	Ordering number	Dilution
CD1c	Super Bright 436	mouse IgG1	L161	eBioscience	62‐0015‐42	1:100
CD3ε	BV605	Mouse IgG1	UCHT1	BioLegend	300460	1:200
CD5	APC/R700	mouse IgG1	UCHT2	BD Biosciences	565121	1:100
CD14	Spark Blue 550	mouse IgG1	M5E2	BioLegend	367147	1:100
CD16	BV650	mouse IgG1	3G8	BioLegend	302018	1:100
CD19	BV650	mouse IgG1	HIB19	BioLegend	302238	1:200
CD20	BV650	mouse IgG2b	2H7	BioLegend	302336	1:200
CD45	PerCP	mouse IgG1	HI30	eBioscience	45‐0459‐73	1:200
CD123	PE/Dazzle 594	mouse IgG1	S18016C	BioLegend	396605	1:100
CD141	BV421	mouse IgG1	1A4	BD Biosciences	565321	1:100
CD169	BUV661	mouse IgG1	7‐239	BD Biosciences	750363	1:100
HLA‐DR	APC/Fire810	mouse IgG2a	L243	BioLegend	307674	1:100
CD88	APC/Fire750	mouse IgG2a	S5/1	BioLegend	344315	1:100

### 2.4 Data analysis

**Figure 2:**
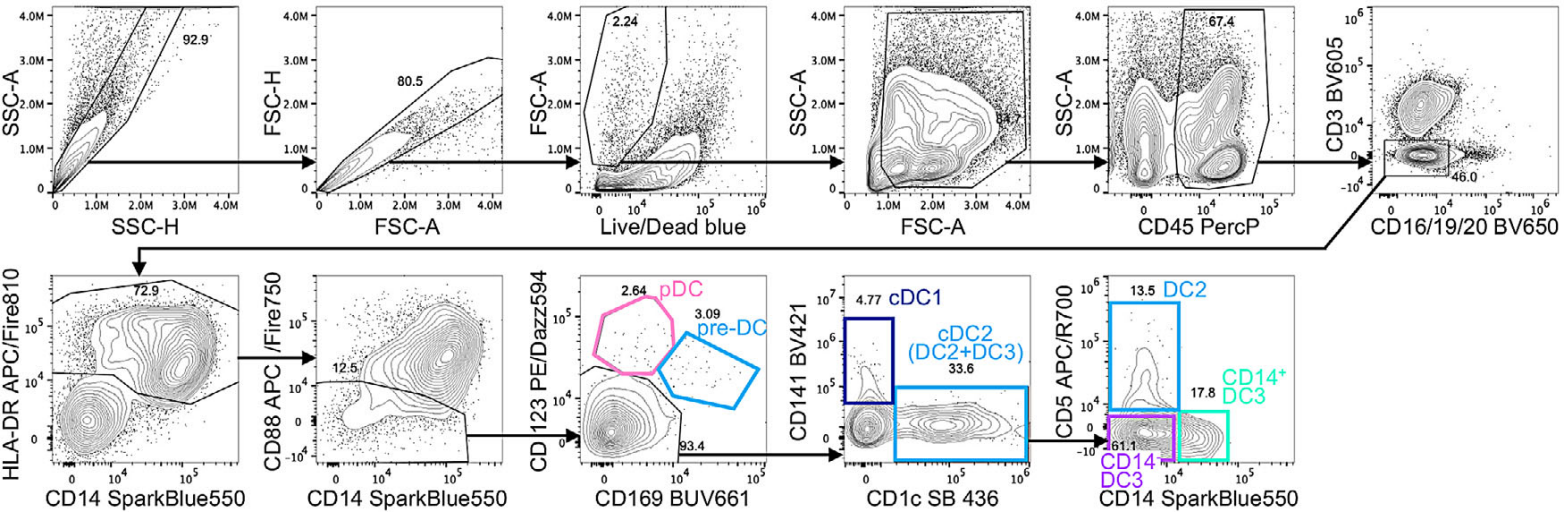
Flow cytometric analyses of human lung DC subsets. As depicted, human lung samples are pregated on single, live, CD45^+^ cells. The CD45^+^ population of the lung then is further gated on CD3^−^ and CD16/CD19/CD20^−^ cells (also called LIN^−^), before gating on all HLA‐DR^+^ cells. From here, monocytes are excluded by gating on CD88^−^ cells only and CD14^−^CD88^−^ and CD14^+^ CD88^−^ are used for subsequent gating on the different DC subsets. Within the CD88^−^ population, pDC can be identified as CD123^+^CD169^−^ cells and pre‐DC as CD123^int/+^CD169^+^ cells. Subsequently, the CD123/CD169 double negative population is further gated on CD141^+^ cDC1 (optional also CADM1^+^) or CD1c^+^ cells, which contain a mixture of cDC2 (DC2 and DC3). These CD1c^+^ cDC2 can be subdivided using CD5 and CD14 and by gating on the CD5^+^CD14^−^ DC2, CD5^−^CD14^−^ DC3, and CD5^−^CD14^+^ DC3 subsets. Samples shown here were acquired on a CYTEK Aurora 5L.

Flow cytometry data acquisition was performed on a CYTEK Aurora 5L flow cytometer. Subsequently, data were analyzed using the FlowJo software (BD). Here, we provide an exemplary gating strategy to properly identify pre‐DC and DC subsets in the human lung, while excluding contaminating monocytes (Fig. 2).

### 2.5 Pitfalls

Given that most lung biopsies come from not‐perfused tissue, these lung samples will contain a mixture of circulating and resident cells.

Depending on the source and handling of the lung biopsy, for example, fresh vs. postmortem, healthy vs. diseased tissue, the cell surface marker, transcription factor expression levels, and/or cytokine secretion capacity may be altered.

Monocytes often can be found contaminating the DC gate. Thus, we recommend adding CD88 to the flow panel to ensure accurate identification of CD88^+^ monocytes vs. CD88^−^ DC.

### 2.6 Top tricks

We recommend working quickly and keeping samples on ice whenever possible for best outcomes, regarding cell viability, numbers, and surface marker phenotypes. Further, the viability of lung cells may vary with each biopsy received and be dependent on healthy versus diseased tissue, how the sample was taken, how the biopsy was kept from sampling till processing (RT vs. on ice), and how much time has passed between initial sampling, processing, and analyses.

While the staining procedure described here is mostly for staining in 5 mL flow cytometry polystyrene tubes, staining in 96‐well round or V‐bottom plates is very commonly used. Especially when handling larger numbers of samples (or performing in vitro assays with cells precultured in 96‐well plates this will save time. However, the use of 96‐well plates may increase the risk of cross‐contamination during staining or wash steps.

We recommend aspirating any supernatant after centrifugation steps, as this will yield consistent staining results. Discarding the supernatant can result in a higher loss of cells while leaving an unknown residual volume that may affect staining dilutions and thus results.

Given the high overlap between surface markers of monocytes and DC, pre‐DC, and pDC, we recommend adding additional surface markers such as CD88 or CD5 to help correctly identify cell populations (see Pitfalls).

CLEC9A is a very good marker to identify human cDC1 and may be used in place of CADM1. CADM1 however has proven as a more reliable cDC1 marker across different species.

If preparing lung DC from different batches/days of preparation for single‐cell RNA sequencing, we recommend adding a common reference population to each analysis (e.g. a certain macrophage population).

We highly recommended to count cell numbers before flow cytometry staining. However, alternatively, for example, CountBright Absolute Counting Beads (#C36950, Invitrogen) may be used. These can simply be added to each sample and acquired during flow cytometry acquisition.

Antibody concentrations/dilutions can differ between tissues. As a guideline for first‐time users, we recommend checking the manufacturer's recommendations when trying a new antibody and titrate dilutions to identify the correct dilution for one's sample. Common dilutions for anti‐human antibodies often can range from 1:20 to 1:200.

In our example, here, we acquired cells using the CYTEK Aurora 5L with standard configuration. The choice of available option of analyzer will affect the antibody selection and panel that can be used to stain one's samples. Thus, getting acquainted with the settings and configuration of the flow cytometer beforehand is recommended. To help adjust the antibody panel to one's flow cytometer, we recommend checking the spectral overlap, fluorochrome brightness, and similarity index using one of the freely online available spectral viewers (for example https://spectrum.cytekbio.com) and/or contacting your local flow cytometry specialist for advice on optimal antibody/fluorochrome “placement”.

The use of LIVE/DEADTM fixable blue dead cell stain for the exclusion of dead cells allows for samples to be fixed (while the use of e.g. DAPI would not). Thus, using this protocol cytokine staining can easily be performed, as well, by adding the appropriate cytokine antibodies.

Keeping an empty channel while acquiring samples on the flow cytometer may be useful to exclude autofluorescence from certain cell populations, for example, macrophages, before gating on DC subsets. The specific channel chosen for this purpose will depend on the flow cytometer used, thus the channels available to begin with, and will help visualize and correct for autofluorescence. Some analyzers specifically allow for the extraction of autofluorescence as an additional marker that then can be used for compensation. However, this needs to be adjusted based on the target cell population. Thus, and in general, we recommend always acquiring unstained cells of the target tissue or target cells, as well, as this will help identify and extract autofluorescence signals.

### 2.7 Summary table

The overall phenotypes of immune cells covered by the markers included in the panel are detailed in Table 7.

**Table 7:** Summary of marker expression on analyzed cell populations.

Population	Marker negative	Marker positive
pDC	Live/Dead, CD3, CD19, CD20, CD16, CD5, CD88, CD169	HLA‐DR^+^, CD123^+^
Pre‐DC	Live/Dead, CD3, CD19, CD20, CD16, CD88	HLA‐DR^+^, CD169^+^, CD123^int/+^, CD5^+^
cDC1	Live/Dead, CD3, CD19, CD20, CD16, CD88, CD123, CD169, CD1c	HLA‐DR^+^, CD141^+^, CADM1^+^
cDC2	Live/Dead, CD3, CD19, CD20, CD16, CD88, CD123, CD169, CD141	HLA‐DR^+^, CD1c^+^
DC2	Live/Dead, CD3, CD19, CD20, CD16, CD88, CD123, CD169, CD14	HLA‐DR^+^, CD1c^+^, CD5^+^
CD14^−^ DC3	Live/Dead, CD3, CD19, CD20, CD16, CD88, CD123, CD169, CD14, CD5	HLA‐DR^+^, CD1c^+^
CD14^+^ DC3	Live/Dead, CD3, CD19, CD20, CD16, CD88, CD123, CD169, CD5	HLA‐DR^+^, CD1c^+^, CD14

## 3 Preparation of single‐cell suspensions from human skin

### 3.1 Introduction

DC are distributed over body surfaces such as the skin and the mucosa. They are perfectly positioned to collect microbial, environmental, and self‐antigens, critical for their main function, namely eliciting specific T‐cell responses. Upon antigen capture and ligation of toll‐like receptors, resident immature DC become activated and migrate into secondary lymphoid organs. There, T‐cell immunity or tolerance is induced. Hence, DC serve as a functional bridge between innate and adaptive immunity [14]. DC can be grouped according to ontogeny or function, the strong stimulatory capacity for naive T cells being the cardinal functional feature of DC, irrespective of their ontogeny [15, 16]. The two main groups of DC are cDC and pDC, the latter are mainly, though not only, present in the blood. The cDC reside in peripheral tissues and secondary lymphoid organs and can be found circulating in the blood [17, 18]. Different DC subsets populate the human skin identified by phenotypical markers [19, 20, 21]. DC in the epidermis are classically termed Langerhans cells (LC). They display high levels of CD1a and Langerin/CD207 [22]. The underlying dermis harbors two well‐defined subsets of dermal DC. The largest subset is characterized by a CD1c^+^CD1a^+^ phenotype and is called dermal cDC2. The CD14^+^ dermal DC population was regarded in the first place as a spontaneously migrating DC population from skin explant culture ex vivo. However, recent transcriptomic profiling showed that they are more closely related to monocytes/macrophages [23]. The cross‐presenting and by far smallest DC subset in the human dermis are BDCA‐3 (CD141)^+^ XCR1^+^ cDC1 [24, 25]. The individual DC subsets not only differ in their phenotype but also have distinct immunological functions [21, 26]. Human LC have been shown to potently induce cytotoxic T cells that can kill melanoma and other cancer cells besides also inducing Th1‐skewed CD4^+^ T‐cell responses [21]. IL‐15, produced by human Langerhans cells appears to play a critical role [27]. However, the dermal cDC1 subset excels in priming cytotoxic T cells as they possess all the required machinery for efficient cross‐presentation [28]. Dermal cDC2 are potent inducers of CD4^+^ T‐helper cell responses such as Th1, Th2, and Th17 as well as humoral responses [21]. For investigations of the diverse skin DC subsets, it is essential to isolate them from human skin for subsequent analysis by flow cytometry or functional assays after further purification steps, for example, magnetic bead isolation and flow cytometric cell sorting. There are several approaches, for example, LC are usually purified after enzymatic separation and digestion of the epidermis [29]. This protocol describes how DC can be isolated from full‐thickness skin.

### 3.2 Materials

#### 3.2.1 Reagents

A complete list of reagents is provided in Table 8

**Table 8:** Reagents, enzymes, chemicals, and solutions.

Reagent	Manufacturer	Ordering number
Gentamicin	Gibco	15750045
RPMI 1640 without glutamine	Lonza	12‐167Q
L‐Glutamine	Lonza	BE17‐605E/U1
Heat‐inactivated FCS	PAN‐Biotech	P30‐3031
Collagenase Type IV	Worthington‐biochem	LS004186
Deoxyribonuclease I (DNAse I)	SIGMA Aldrich	DN‐25
Hank's salt solution with calcium and magnesium, without phenol red	Pan‐Biotech	P04‐32105
Dulbecco's PBS without calcium and magnesium	Gibco	14190‐094
CaCl_2_	SIGMA	449709‐10G
Trypan blue	SIGMA	T8154‐100ML

#### 3.2.2 Equipment

The necessary equipment are listed in Table 9.

**Table 9:** Necessary equipment.

Equipment	Company	Purpose
Scalpel	Aesculap	Trimming of subcutaneous fat from dermis
Forceps	Aesculap	Holding the skin while trimming off subcutaneous fat and during cutting of 5×5 mm pieces for digestion step
50 mL canonical tube	FALCON	For disinfection of skin pieces in RPMI‐gentamycin solution and centrifugation of cell suspensions
Large petri dish (100 × 15 mm)	FALCON	Skin preparation (trimming of fat and cutting into smaller pieces)
Six‐well plates	Thermo Scientific	Skin digestion
Small petri dish (60 × 15 mm)	FALCON	Skin digestion
Serological pipettes (5 mL/10 mL/25 mL)	Greiner Bio‐One	Pipetting
100 µm filter for 50 mL tubes (#352360)	Corning	Filtering cell suspension
2 mL syringes Discardit II	BD Biosciences	Plunger used to press tissue through cell strainer
Incubator	Thermo Scientific	For skin digestion at 37°C
Centrifuge	Heraeus Multifuge 3 S‐R	Centrifugation of FACS tubes
Neubauer chamber 0.100 mm; 0.0025 mm^2^	Superior Marienfeld	Cell counting with a hemocytometer

### 3.3 Step‐by‐step sample preparation

#### 3.3.1 Preparation of stocks and solutions

##### 3.3.1.1 Disinfection medium

RPMI medium supplemented with 50 µg/mL gentamicin. Store at 4°C for max. 3 weeks.

##### 3.3.1.2 R10 culture medium

RPMI medium supplemented with 10% heat‐inactivated FCS, 2 mM l‐glutamine, and 50 µg/mL gentamicin. Store at 4°C for max. 3 weeks.

##### 3.3.1.3 Collagenase Type IV

For preparing the 5 mg/mL stock solution, dissolve 50 mg Collagenase Type IV in 10 mL Hank's balanced salt solution (with Ca^2+^ and Mg^2+^) and store aliquots at −20°C.

##### 3.3.1.4 DNAse I

Dissolve 1 g DNAse I in 100 mL 5 mM calcium chloride (dissolved in Aqua dest). Sterile filter the 10 mg/mL DNAse I solution through a 0.22 µm Stericup and store aliquots at −20°C.

#### 3.3.2 Skin preparation

1. Healthy human skin is obtained from plastic surgery, usually breast and abdomen reduction but also whole body lifting.
2. For disinfection, transfer skin pieces into a 50 mL tube filled with 30 mL RPMI and 30 µL gentamicin and incubate for at least 30 min. The skin should be completely immersed in the medium.
3. Transfer skin pieces into a large sterile petri dish with dermal side up.
4. Cut into smaller skin pieces (approx. 5 × 3 cm size) in the petri dish.
5. Hold skin with one strong forceps and trim off subcutaneous fat with a scalpel.
6. Turn skin pieces around and transfer them to a small sterile petri dish with epidermal side up.
7. Cut into small 5 × 5 mm pieces for skin digestion.

#### 3.3.3 Enzymatic digestion of the skin

  1. Prepare enzyme solution: 2 mL R10‐medium and 500 µL collagenase IV (1:5, end concentration 1 mg/mL).
(1) Pipette enzyme solution into a small petri dish with 5 × 5 mm skin pieces.
(2) Skin pieces are digested at 37°C for 6 h minimum or overnight (max. 16 h).
(3) Following digestion, gently pipette skin pieces several times up and down and filter through a 100 µm cell strainer, press tissue through the cell strainer with the plunger of a syringe and wash the Petri dish and cell strainer by topping up to 50 mL with R10‐medium.
(4) Centrifuge at 485 × *g* for 5 min at 4°C and discard supernatant
(5) Resuspend cells in 1–2 mL R10 medium, count cells with a hemocytometer, and calculate the percentage of dead cells (typically not more than 20–30%).
(6) The cell numbers vary depending on donor skin but should be between 2 and 5 Mio cells from a 3 × 5 cm skin piece digested in one small Petri dish.

### 3.4 Data analysis

Examples of flow cytometry data analysis of DC subsets in human skin using the described single cell preparation are covered in detail in the section **4** **Flow cytometry analysis of DC subsets in human skin**.

### 3.5 Pitfalls

1. Remove fat carefully from the skin before starting the cutting procedures. To allow for optimal digestion ensure that the fat‐free skin is cut into 5 × 5 mm or even smaller pieces.
2. Longer digestion times can decrease surface marker expression, for example, CD1a but does not result in complete loss of markers.
3. Longer digestion times might induce activation of DC, for example, CD40, CD80, and CD86 upregulation.

### 3.6 Top tricks

1. If surgeons can provide dermatomized skin, a thickness of 1 mm works well and allows intradermal injections of reagents before skin preparation. Incubation with collagenase/DNase digestion mix overnight improves cell yields but might compromise surface marker expression.
2. Digestion in an FCS‐containing medium decreases the enzyme activity of collagenase but might help to preserve surface marker expression. A direct comparison of the medium with/without FCS could be used for the optimization of the flow cytometry panel.

## 4 Flow cytometry analysis of DC subsets in human skin

### 4.1 Introduction

Human skin comprises our outermost barrier against invading pathogens, toxins, and other harmful environmental influences. To maintain the homeostasis of our body, human skin has important physicochemical properties like the layer of corneocytes, an acidic pH, the production of antimicrobial peptides, and many others [30, 31]. On top of that, numerous immune cells of the innate and adaptive type are present in the skin. Most notable are DC that act as immune sentinels in our body's outermost barrier. DC can react to pathogen invasion or cell damage, which leads to their activation and migration to the draining lymph node (LN). Equipped with information on the invading pathogen, DC arrive in the LN and promote T‐ and B‐cell response [32, 33]. As such, DC are important key players in immune responses, linking the innate and adaptive immune response, but also maintaining tolerance.

Distinct DC subsets can be identified in the epidermal and dermal layers of the skin. In the epidermis, the only DC‐type are the epidermal LC, which are characterized by the surface molecule Langerin (CD207), CD1a, HLA‐DR, and CD1c [34, 35]. Another classical feature of LC is the tennis‐racket‐shaped intracellular organelles, called Birbeck granules [36].

The dermal layer contains mainly cDC, most abundantly cDC2 characterized by CD11c, CD1a (lower than LC), CD1c, and the transcription factor IRF4 [26, 37]. Next to cDC2, the dermis hosts a small population of cDC1 that can be distinguished from cDC2 by XCR1, high CD141, and low CD11c expression [25]. The cDC1 expresses the transcription factors IRF8 and BATF3 [25]. Besides cDC, the dermis also contains CD14^+^ cells, formally identified as DC, which are of monocytic origin and transcriptionally similar to FXIIIa^+^ macrophages [23]. Although these CD14^+^ cells are macrophage‐like, they emigrate in skin explant culture, which means they possess migratory capacities [23]. Dermal macrophages are highly autofluorescent (AF) and express CD14, CD163, and FXIIIa [26]. pDC are absent in healthy human skin but can be recruited under chronic inflammatory conditions and are then identified by the expression of CD123 [38]. Dermal B cells, which are also antigen‐presenting cells, can also be observed although in very low numbers and it is still a matter of discussion if they are skin‐resident immune cells [39].

The following protocols describe a 14‐color flow cytometry panel for the identification of DC from healthy human whole‐thickness skin digested with type IV collagenase (as described in the section **2** **Flow cytometric analysis of the human lung DC compartment**). The panel enables the separation of LC, cDC1, and cDC2 from T cells, B cells, and macrophage‐like CD14^+^ cells. Additionally, costimulatory molecules CD40, CD86, and CD80 further allow the analysis of the activation status of epidermal and dermal DC.

### 4.2 Materials

#### 4.2.1 Reagents

A complete list of reagents is provided in Tables 10 and 11.

**Table 10:** Reagents, enzymes, chemicals, and solutions.

Reagent	Manufacturer	Ordering number
Chemicals and solutions		
Dulbecco's PBS without calcium and magnesium	Gibco	14190‐094
AccuGENE 0.5 M EDTA solution	Lonza AccuGene	51201
Deoxyribonuclease I (DNAse I)	Sigma‐Aldrich	DN25
BSA (albumin bovine fraction V)	SERVA	11930
Fixable viability stain 440UV	BD Biosciences	566332
Human BD Fc block	BD Biosciences	564220
Brilliant stain buffer	BD Biosciences	563794

**Table 11:** Reagents and antibodies used for flow cytometric analysis.

Specificity	Fluorochrome	Clone	Manufacturer	Ordering number	Dilution
CD40	FITC	5c3	BioLegend	334306	1:50
CD14	BB630P	MΦP9	BD	Custom	1:200
CD1c	PE	AD5‐8E7	Miltenyi	130‐110‐536	1:100
CD11c	PE‐Cy7	B‐ly6	BD	561356	1:1000
CD141	APC	AD5‐14H12	Miltenyi	130‐113‐314	1:125
CD19	APC‐R700	HI98	BD	564977	1:400
CD16	APC‐eFluor780	eBioCB16	ThermoFisher	# 47‐0168‐42	1:50
CD207	VioBlue	MB22‐9F5	Miltenyi	130‐106‐147	1:50
HLA‐DR	BV480	G46‐6	BD	566113	1:200
CD80	BV650	L307.4	BD	564158	1:40
CD86	BV786	IT2.2	BioLegend	305442	1:500
Viability dye	440UV		BD	566332	1:250
CD3	BUV737	UCHT1	BD	612750	1:200
CD45	BUV805	HI30	BD	564914	1:200

#### 4.2.2 Equipment

The necessary equipment are listed in Table 12.

**Table 12:** Necessary equipment.

Equipment	Company	Purpose
1.5 mL or 2 mL reaction tube	Eppendorf	preparation of staining mix, live/dead dye solution, Blocking buffer
5 mL polystyrene round bottom tubes	FALCON	For cell staining and analysis
50 mL canonical tube	FALCON	Store cells after isolation
Serological pipettes (5 mL/10 mL/25 mL)	Greiner Bio‐One	Washing
CellTrics filter, 30 µm	Sysmex	For filtering cells before flow cytometry
Centrifuge	Heraeus Multifuge 3 S‐R	Centrifugation of cells during washing steps
Aurora spectral flow cytometer	Cytek	Flow cytometry analysis

### 4.3 Step‐by‐step sample preparation

#### 4.3.1 Preparation of stocks and solutions

Staining buffer: PBS supplemented with 1% BSA, 50 µM EDTA, and 50 µg/mL DNAse I.

Live/dead dye solution: Dilute Fixable Viability Stain 440UV 1:250 in PBS.

Blocking buffer: Use 2.5 µg Fc block for 10^6^ cells diluted in staining buffer.

Brilliant staining buffer: Dilute brilliant stain buffer according to manufacturer instructions (brilliant stain buffer available as 2× or 10× concentrate).

#### 4.3.2 Isolation and preparation of single‐cell suspensions from human skin

In the section **3** **Preparation of single‐cell suspensions from human skin**, we provide a detailed protocol for isolating cells from human whole skin tissue for analysis by flow cytometry.

#### 4.3.3 Antibody staining protocol for human skin single‐cell suspensions

1. Use between 3–4 × 10^6^ cells per staining.
2. Resuspend cells in 100 µL freshly prepared live/dead dye solution.
3. Incubate for 20 min at 4°C in the dark.
4. Wash cells by adding 1 mL of staining buffer.
5. Centrifuge at 485 × *g* for 5 min at 4°C.
6. Resuspend cells in 100 µL of blocking buffer.
7. Incubate for 15 min at 4°C, protected from light.
8. Wash cells by adding 1 mL of staining buffer.
9. Centrifuge at 485 × *g* for 5 min at 4°C.
10. Resuspend cells in 100 µL of antibody staining mix containing the correct final dilution in brilliant staining buffer (see Table 13).
11. Incubate for 30 min at 4°C in the dark.
12. Wash cells by adding 1 mL of staining buffer.
13. Centrifuge at 485 × *g* for 5 min at 4°C.
14. Resuspend cells in 200–300 µL of Staining buffer and keep in the dark at 4°C until analysis on Cytek Aurora. Before analysis, filter the cell through a 30 µm cell Trics filter.

**Table 13:** Staining workflow for human skin DC panel.

	Marker	Fluorochrome	Dilution	Diluent	Incubation (min/temp)
**1**	Viability dye	440UV	1:250	PBS	20’/4°C
					
**2**	Wash (staining buffer)				
	
**3**	Blocking buffer	–	2.5 µg/10^6^ cells	Staining buffer	15’/4°C
					
**4**	Wash (staining buffer)				
	
**5**	CD40	FITC	1:50	Brilliant stain buffer	30’/4°C
	CD14	BB630	1:200		
	CD1c	PE	1:100		
	CD11c	PE‐Cy7	1:1000		
	CD141	APC	1:125		
	CD19	APC‐R700	1:400		
	CD16	APC‐eFluor 780	1:50		
	CD207	VioBlue	1:50		
	HLA‐DR	BV480	1:200		
	CD80	BV650	1:40		
	CD86	BV786	1:500		
	CD3	BUV737	1:200		
	CD45	BUV805	1:200		
					
**6**	1× wash (staining buffer)				
					
**7**	Resuspend cells in 200–300 µL of staining buffer and keep in the dark at 4°C until acquisition				

A brief overview of the staining workflow is shown in Table 13.

### 4.4 Data analysis

Data acquisition was performed with a Cytek Aurora spectral flow cytometer equipped with 5 lasers and 64 detectors enabling full spectrum cytometry. The Cytek Aurora allows for the measurement of the entire emission spectra for each fluorochrome, across all lasers, compared with classical flow cytometry, which measures the peak emission of every fluorochrome [40, 41, 42]. Data were analyzed using FlowJo software.

**Figure 3:**
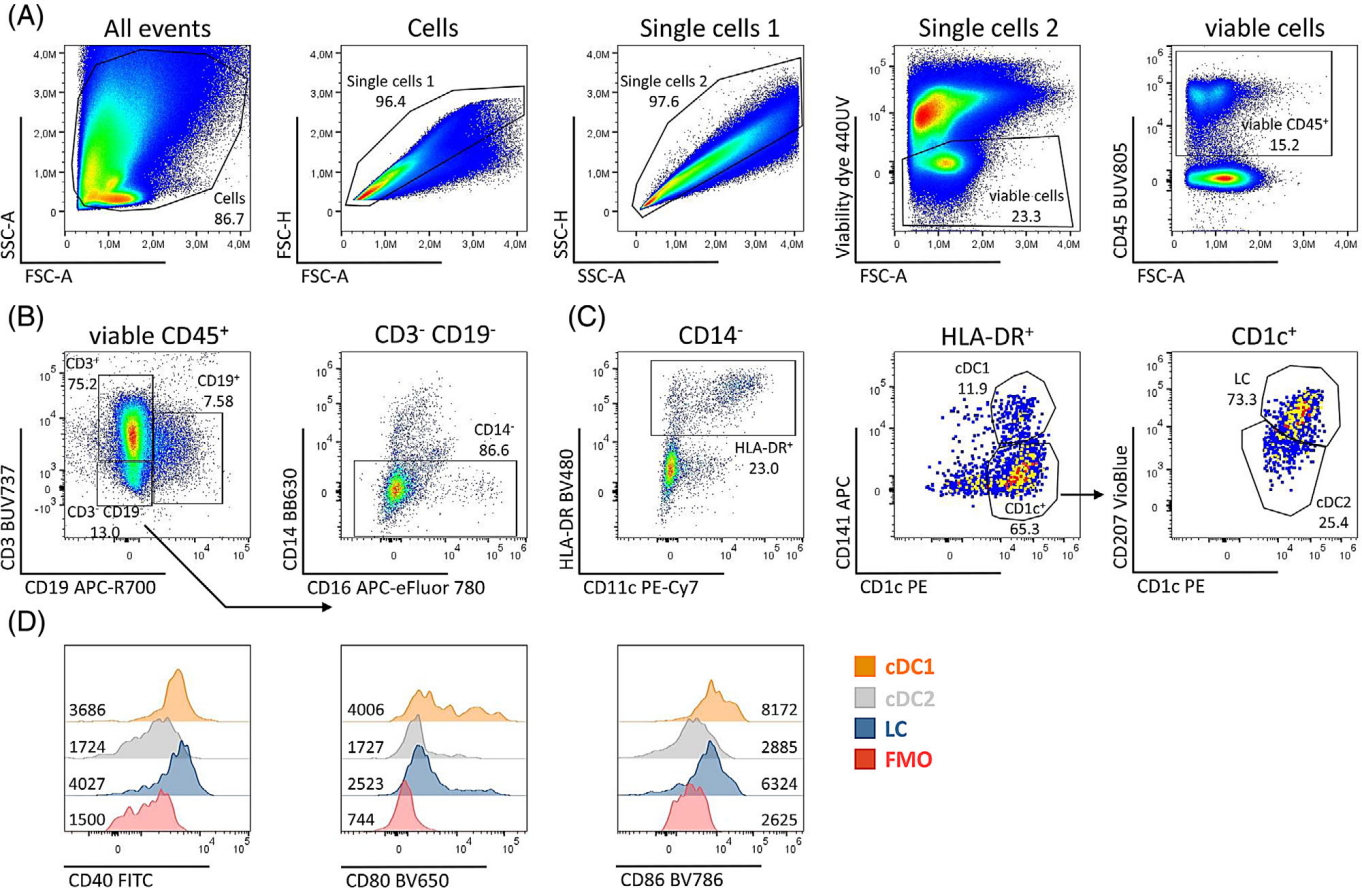
Gating strategy for flow cytometry analysis of human skin. The skin was enzymatically digested to generate single‐cell suspensions. (A) Gating strategy for viable CD45^+^ cells after exclusion of cellular debris, doublets, and dead cells. (B) CD3^+^ T cells and CD19^+^ B cells as well as CD14^+^ cells were excluded before DC characterization. (C) HLA‐DR expressing cells comprise DC, which can be further subdivided into CD141^+^ CD1c^+^ cDC1 and CD1c^+^ subsets, consisting of CD207^+^ LC and CD207^−^ cDC2. (D) Surface expression of co‐stimulatory markers CD40, CD80, and CD86 on the indicated DC subsets: cDC1 (orange), cDC2 (grey), and LC (blue). MFI values are shown. cDC, conventional DC; LC, Langerhans cells.

Fig. 3 shows a representative gating strategy for the identification of immune cell populations of human healthy skin. After removing cellular debris, dye aggregates, and cellular doublets, dead cells were excluded from the analysis using the fixable viability dye 440UV. CD45 allows the separation of the hematopoietic cell lineage from contaminating epithelial and mesenchymal cells (Fig. 3A). Next, CD3^+^ T cells and CD19^+^ B cells were identified. From the CD3^−^ CD19^−^ population, CD14‐expressing cells, which represent monocytic and macrophage‐like cells [23] were excluded for the subsequent DC analysis (Fig. 3B). We proceeded with the CD14‐ cells and gated DC based on their HLA‐DR expression (Fig. 3C). Most of these HLA‐DR^+^ cells were CD11c‐positive except for the CD141^+^ cDC1 as already described earlier [25]. The single CD1c^+^ DC can be further subdivided into Langerin/CD207^+^ LC and CD207^−^ dermal cDC2 (Fig. 3C). Note here we used dermatomized skin, so the dermal cDC2 compartment is underrepresented when compared with full‐thickness skin. By including antibodies against costimulatory molecules, CD40, CD80 and CD86, the activation status of DC subsets can be further analyzed. LC and cDC1 show slightly higher levels of CD40 and CD86 than cDC2 (Fig. 3D).

### 4.5 Pitfalls

1. Human tissue samples, even when obtained from healthy individuals, have a strong donor‐to‐donor variability. This can cause varying frequencies of immune cell populations detected by flow cytometry analysis of skin from different donors and sometimes even donor‐specific compensation is required.
2. Another important point to consider is the age of the donor and the extraction of the skin. Aged skin becomes thinner and shows increased water loss as well as fragmentation of collagen and elastin. Besides this, aging skin contains less LC and more regulatory T cells [43].
3. Furthermore, it is important to consider whether full‐thickness or dermatomized skin is used. Dermatomized skin lacks the full dermal compartment. Therefore, dermal cDC subsets might be underrepresented in this particular protocol, which is the case in the example shown in this section.
4. The length of the enzymatic digestion step might affect cell surface markers, for example, CD1a, and can also cause activation of DC. Thus, surface markers need to be tested in this regard. Cellular debris can interfere with the staining. Make sure to vortex the cell suspension thoroughly after adding a new staining.

### 4.6 Top tricks

#### 4.6.1 Antibody titration

All used antibodies were titrated, either to a selected optimal concentration or to saturation. Optimal antibody concentrations were considered the lowest amount of antibody that showed the best signal separation with minimal background staining. Prefixed dilutions need to be adapted when used on other instruments.

#### 4.6.2 Blocking buffer

Alternatively, to commercially available Fc Block reagents, 3% mouse serum can be used as most anti‐human antibodies are mouse monoclonal.

#### 4.6.3 Single color reference control

Using cells as single‐stained reference controls is superior to beads in our experience. Therefore, we used beads only in cases where we could not achieve a proper separation of positive and negative signals with the original antibody or the dummy approach (substitute with the same fluorochrome from the same company conjugated to an antibody against an abundantly expressed marker). Correct unmixing was monitored with single stained cells. Spectral unmixing is the mathematical method used to differentiate the fluorescence signals from each fluorochrome in an experiment. A major advantage of unmixing in comparison to conventional compensation is that autofluorescence can be handled as a separate parameter, making it possible to extract the autofluorescence of a sample. However, this method can only be applied to spectral flow cytometry. This can be very useful when working with tissues that exhibit a high autofluorescence, which might affect the resolution of the other fluorescent signals.

#### 4.6.4 Brilliant stain buffer

When using two or more BD Brilliant dye‐conjugated antibodies, we recommend using Brilliant Stain Buffer, as fluorescent dye interactions might lead to staining artifacts.

#### 4.6.5 Panel optimization

For future use, several adaptions or extensions of the panel are possible. For identification of cDC1 antibodies for XCR1 can be added, for identification of LC also combinations of CD1a^high^Langerin^+^ can be used. A fixation step after staining for surface molecules would allow including additional intracellular markers, for example, cytokines or transcription factors. For a comparison of inflamed or tumorigenic skin versus healthy skin, we suggest additionally including CD123 for the analysis of pDC.

### 4.7 Summary of the phenotype

Table 14 gives an overview of the phenotype of the analyzed DC subpopulations in human skin as shown in Fig. 3.

**Table 14:** Summary of marker expression on skin DC populations.

	CD141^+^ cDC1	LC	cDC2
CD14	−	−	−
CD1c	+	+	+
CD141	+	−	−
CD207	−	+	−
HLA‐DR	+	+	+

## 5 Preparation of single‐cell suspensions of human gingiva

### 5.1 Introduction

**Figure 4:**
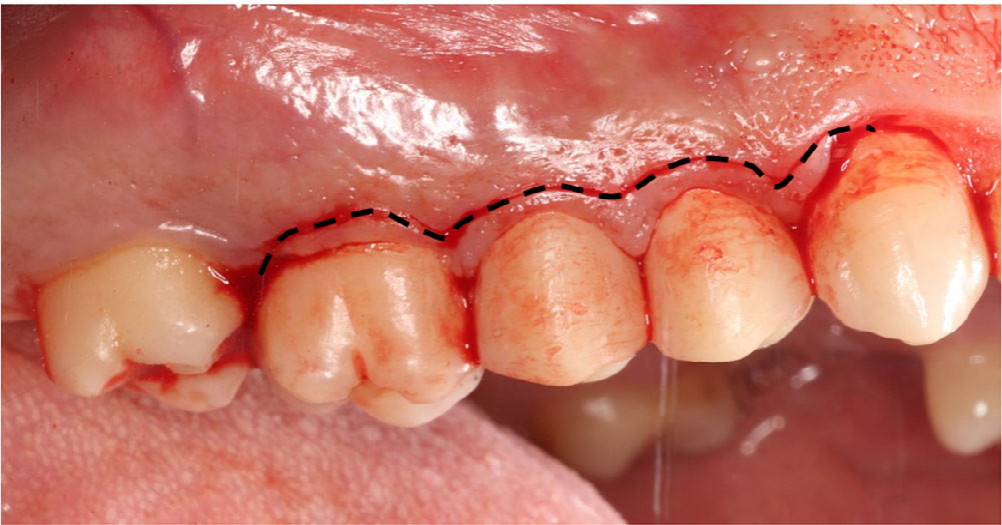
Dissection of the gingiva from the masticatory mucosa. A buccal view of the upper right molars, premolars, and an incision in the gingiva. The gingiva is part of the masticatory mucosa that surrounds the teeth. Using a sharp No. 15c scalpel blade, dissect a sample of fresh gingiva that includes the lamina propria and epithelium. The minimal sample size is 3 × 2 mm (∼15 mg). In this case, a 15 × 2 mm tissue sample was harvested (black dashed line).

**Figure 5:**
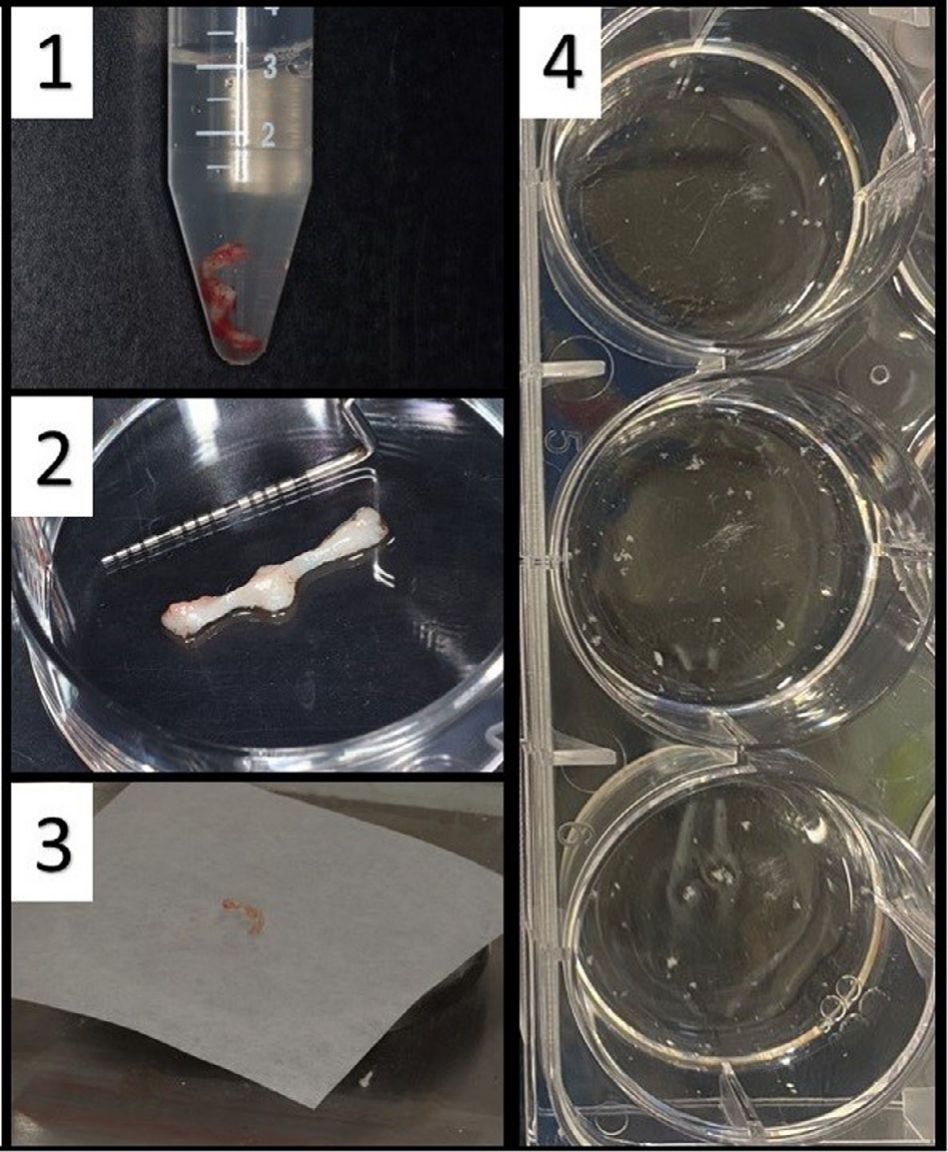
Processing the fresh gingival sample. (1) After dissecting the gingival sample, store the sample in 3 mL of FACS buffer (see the section **5**) in a 15 mL tube. (2) Clean the blood from the fresh sample, dry it carefully, and (3) weigh the tissue. (4) Place the sample in a six‐well plate. If the sample is bigger than 3 × 2 mm, divide it into 3 × 2 mm pieces and place each piece in a separate well. Chop the tissue into tiny pieces (smaller than 1 × 1 mm) using a no. 10 scalpel blade to allow better contact between the tissue and the digestion mix (see the section **5**). Add 1000 µL of digestion mix to each well.

The oral mucosa has direct contact with the external environment, and, therefore, it is constantly exposed to various physical, chemical, and microbial challenges. To protect the underlying tissues, the oral mucosa acts as a physical and immunological barrier that prevents pathogens from invading the tissue and causing damage. The oral mucosa is continuous with the skin of the lips and the mucosa of the soft palate and pharynx. It consists of three distinct types of mucosae: (1) the specialized mucosa, which covers the dorsum of the tongue and contains the taste buds, (2) the lining mucosa at the buccal aspects of the oral cavity, which is a non‐keratinized tissue, and (3) the masticatory mucosa, which includes the gingiva and the covering of the hard palate [44]. This third type of mucosa is keratinized and, therefore, protects the oral cavity from mechanical forces. The gingiva is composed of an epithelial layer and underlying connective tissue called lamina propria. Each layer has distinct characteristics and harbors specific immune cells [45]. The leukocytes residing within the gingiva are in close proximity to the dental biofilm and, hence, are essential for the establishment and maintenance of local immune homeostasis [46]. The stable host–microbial homeostasis is fundamental for maintaining periodontal health. When the immune homeostasis in the gingiva is dysregulated, periodontal disease and other pathologies can develop [47]. While the role of DC in mouse gingival immunity has been extensively studied, the importance of these cells in the human gingiva requires further elucidation. Thus, there is a need to characterize DC subpopulations in different compartments of the human gingiva. Herein, we present a detailed protocol for the dissection and processing of human gingival tissue to obtain a single‐cell suspension for flow cytometry analysis (Fig. 4 and 5).

### 5.2 Materials

#### 5.2.1 Reagents

A complete list of reagents is provided in Table 15.

**Table 15:** Reagents, antibodies, chemicals, and solutions.

Reagent	Manufacturer	Catalog number
Enzymes		
Collagenase, Type 2, CLS‐2	Worthington	WOLS04177
Deoxyribonuclease I (DNAse I)	Sigma	DN25‐1G
Chemicals and solutions		
Dulbecco's PBS 10×	Sigma	D1408‐6×650ML
Fetal bovine Serum (FBS)	Biological Industries	04‐127‐1A
Ethylenediaminetetraacetic acid (EDTA, 0.5M, pH 8.0)	BioPrep	EDTA‐500ML

#### 5.2.2 Equipment

The necessary equipment are listed in Table 16.

**Table 16:** Necessary equipment.

Equipment	Company	Purpose
Surgical scalpel blade no. 15c	Swann‐Morton	For dissection of the fresh gingiva sample from the oral mucosa. Alternatively, other blades can be used.
Surgical scalpel blade no. 10	Swann‐Morton	For chopping the tissue into tiny pieces
Periosteal elevator PH2 or Molt 9	Hu‐Friedy	To separate and elevate the fresh gingival sample from the oral mucosa
No. 31 Straight Semkin‐Taylor tissue plier	Hu‐Friedy	To hold the gingival sample
Finnpipette F3	Thermo Scientific	For pipetting cell suspension
15ml polypropylene centrifuge tubes	Miniplast Ein‐Shemer	Storage of the sample and centrifugation of cell suspensions
Six‐well plate	Thermo Fisher Scientific	Storage of oral tissues
70 µm filters	Falcon	For filtration of cells to generate single‐cell suspensions from oral tissue
Centrifuge 5810 R	Eppendorf	For centrifugation of cell suspensions
Incubator	Tuttnauer 12950XL	Incubator for tissue digestion
Invitrogen Countess II automated cell counter	Thermo Fisher Scientific	For counting cells

### 5.3 Step‐by‐step sample preparation

#### 5.3.1 Preparation of buffer and digestion mix

##### 5.3.1.1 Fetal bovine serum

Thaw FCS in a 37°C water bath. Once completely thawed, incubate for 60 min in a 56°C water bath to destroy complement activity. Aliquot the FCS into 50 mL portions and store at −20°C. Avoid freeze‐thaw cycles. Use aseptic techniques during the entire procedure.

##### 5.3.1.2 FACS buffer

Prepare a 2% FCS/PBS solution (v/v) by adding 10 mL of heat‐inactivated FCS to 490 mL of 1× PBS.

##### 5.3.1.3 Digestion mix

Prepare FACS buffer containing 2 mg/mL Collagenase II and 1 mg/mL DNase I. Make 1 mL digestion mix per sample.

#### 5.3.2 Isolation of mucosal tissues from the oral cavity

1. Proper local anesthesia.
2. Using a sharp no. 15c scalpel blade (or another appropriate blade/instrument), dissect a sample of fresh gingiva of at least 3 × 2 mm (∼15 mg).
3. Separate the dissected tissue from the oral cavity using a periosteal elevator and tissue plier.
4. Store the sample in a 15 mL tube containing 3 mL FACS buffer.
5. Insert the tissue sample into one well of a six‐well plate, clean the fresh sample from blood using a no. 10 scalpel blade, and then transfer the cleaned sample to a new well.
6. Carefully dry and weigh the sample.

#### 5.3.3 Preparation of single‐cell suspensions from oral mucosal tissues

1. The minimal size of a sample is 3 × 2 mm (∼15 mg). If the sample is bigger, divide it into 3 × 2 mm pieces. Place each piece in a separate well of a six‐well plate.
2. Chop the tissue into very small pieces (smaller than 1 × 1mm) using a no. 10 scalpel blade.
3. Add 1000 µL of digestion mix per sample.
4. Incubate the samples for 20 min in an incubator at 37°C.
5. Add 20 µL of 0.5 M EDTA per sample (final concentration of 10 mM), shake gently, and incubate for another 10 min at 37°C.
6. Pipette the sample up and down several times and pass it through a 70 µm cell strainer into a 15 mL tube.
7. Wash each well with 2 mL FACS buffer, and add the wash to the cell strainer.
8. Wash the cell strainer with an additional 10 mL FACS buffer.
9. Centrifuge the cells at 314 × *g* for 5 min at 4°C.
10. Discard the supernatant.
11. Resuspend the cells in 500 µL FACS buffer.
12. Count the cells and keep them on ice for further analysis.

### 5.4 Data analysis

Examples of flow cytometric data analysis of oral DC subsets using the described single cell preparation are discussed in detail in the section **6** **Flow cytometry analysis of human dendritic cell subsets in the gingiva**.

### 5.5 Pitfalls

1. To analyze subpopulations of rare cell types, such as DC, we recommend using a sample of at least 3 × 2 mm, which is equivalent to approximately 15 mg.
2. For a sample larger than 3 × 2 mm, use a proportional amount of digestion mix and number of wells in a six‐well plate.
3. To increase the viability of the cells, process the tissue promptly after dissecting the fresh gingiva sample, keep the samples on ice, and process the single‐cell suspension quickly.
4. Sharp blades help chop the sample into very small pieces.
5. To examine the gingiva around the teeth, we recommend dissecting the tissue no more than a 2 mm distance from the teeth.

### 5.6 Top tricks

1. In this case, the 15 × 2 mm^2^ sample was divided into six pieces to increase the contact area of the fresh gingival sample pieces with the digestion mix enzymes.
2. Prior to harvesting the tissue, prepare the protocol and reagents that will be needed after reaching a single cell suspension, for example, assemble buffers and antibodies for FACS staining.
3. Although FBS is known for decreasing collagenase activity, the viability of gingival cells is very sensitive and it is important to preserve it and the expression of surface markers. Thus, we decided to use a low concentration of FBS buffer (2%), which seemed to have the maximal effect.
4. The cells obtained with this protocol can be subsequently used not only for flow cytometry as described in the section **6** **Flow cytometry analysis of human dendritic cell subsets in the gingiva** but also for transcriptomics as well as functional assays.

## 6 Flow cytometry analysis of human dendritic cell subsets in the gingiva

### 6.1 Introduction

DC are a heterogeneous population of antigen‐presenting cells that are a part of the innate immune system. They exist in all lymphoid and most nonlymphoid tissues, including the gingiva. As professional antigen‐presenting cells, they bridge the innate and adaptive immune response and consequently have an essential role in establishing immunological memory [48]. In their immature state, DC constantly patrols the tissue for pathogens [49]. Upon encountering a foreign antigen, DC become activated and migrate to the draining lymph nodes while undergoing a maturation process enabling them to present antigens to CD4^+^ and CD8^+^ T cells [50]. By polarizing CD4^+^ T cells to the different subsets, DC play an important role in orchestrating the balance between immunity and tolerance. Thus, DC are critical for immune homeostasis, whereas impairment of their function may result in inflammatory diseases such as periodontitis [46, 51].

DC are derived from bone marrow CD34^+^ hematopoietic stem cells and can be subdivided into pDC and cDC [15]. They express high levels of HLA‐DR and lack the lineage markers CD3, CD19, CD20, and CD56 [25]. To date, four distinct subsets of antigen‐presenting cells (APC) were identified in healthy gingiva: conventional DC type 1 (cDC1), conventional DC type 2 (cDC2), LC, and pDC. During DC differentiation, the cells pass through an intermediate stage in which they are referred to as pre‐DC and express the markers CD123, CD45RA, AXL, and CD5. After final differentiation into DC, they are divided into cDC1 and cDC2, which are distinguished from each other by markers that are specific to each cell type: cDC1 cells express CD141 and CLEC9A, while cDC2 cells express CD1c [52]. The cDC2 population is the more abundant of the two cells in the gingiva and blood.

LC in the oral mucosa are a special subtype of APC derived from both monocytic precursors and preDC in mouse [53]. They reside in the oral mucosal epithelium where they constitute the most significant antigen‐presenting cell population. LC in the oral cavity express two major markers, CD207 (Langerin) and epithelial cell adhesion molecule (Epcam). These two markers represent the terminal differentiation of the cell.

pDC are a unique population of DC that secrete IFN‐1 in response to infection. pDC are characterized by the expression of CD123 and CD45RA, which distinguish them from other DCs. They do not express the pre‐DC markers, AXL or CD5.

Although DC were extensively studied in mice and some of the niches in human tissue, our understanding and characterization of these cells in the human gingiva remains limited. Therefore, it is important to study these cells in the gingiva to better understand their role and function in health and disease. Recently, we demonstrated that in the gingiva of periodontitis patients, LC are decreased while pDC were more abundant than in healthy gingiva [54]. Here, we present a detailed description of how to discriminate between the various DC subsets in human gingiva using flow cytometry.

### 6.2 Materials

#### 6.2.1 Reagents

A complete list of reagents is provided in Table 17.

**Table 17:** Reagents, antibodies, chemicals, and solutions.

Reagent	Manufacturer	Catalog no.
Antibodies		
CD123 anti‐human BUV395	BD	564195
HLA‐DR anti‐human APC‐R700	BD	565127
CD3 anti‐human BUV661	BD	741596
CD56 anti‐human BUV737	BD	612766
Axl anti‐human Alexa Fluor 647	R&D system	FAB154R‐100UG
CD45 anti‐human Brilliant Violet 750	BioLegend	368542
CD45RA anti‐human Brilliant Violet 711	BioLegend	304138
CD5 anti‐human Brilliant Violet 605	BioLegend	364020
CD1c anti‐human Brilliant Violet 650	BioLegend	331542
CD207 (Langerin) anti‐human APC	BioLegend	352206
CD141 anti‐human PerCP/Cyanine5.5	BioLegend	344112
CD66b anti‐human PE/Cyanine7	BioLegend	305126
CD19 anti‐human Brilliant Violet 510	BioLegend	302242
Zombie UV Fixable Viability Kit	BioLegend	423107
CD326 (EpCAM) anti‐human VioBlue	Miltenyi Biotec	130‐113‐266
Chemicals and solutions		
Brilliant stain buffer	BD	563794
Dulbecco's PBS without calcium and magnesium	Sigma	D8537
Fetal bovine serum (FBS)	Biological Industries	04‐127‐1A
Cytofix/Cytoperm fixation and permeabilization solution	BD	554722

#### 6.2.2 Equipment

The necessary equipment are listed in Table 18.

**Table 18:** Necessary equipment.

Equipment	Company	Purpose
Centrifuge 5810 R	Eppendorf	For centrifugation of cell suspensions
Finnpipette F3	Fisher Scientific	For pipetting cell suspensions
1.7 mL Eppendorf tubes	LIFE GENE	For the preparation of antibody staining mix
15 mL tubes	Greiner bio‐one	Centrifugation of cell suspensions
50 mL tubes	Greiner bio‐one	Aliquot of FBS
70 µm filters	Falcon	For filtration of cells to generate single‐cell suspensions from oral tissue
FACS tubes	BD	For sample acquisition at the flow cytometer
Cytek Aurora Flow Cytometry System	Cytec	For flow cytometric analysis of single‐cell suspensions

### 6.3 Step‐by‐step sample preparation

#### 6.3.1 Preparation of stocks and solutions

##### 6.3.1.1 Fetal bovine serum

Thaw FBS in a 37°C water bath. Once completely thawed, incubate for 60 min in a 56°C water bath to heat inactivate complement. Aliquot the FBS into 50 mL portions and store at −20°C. Avoid freeze‐thaw cycles. Use aseptic techniques throughout the procedure.

##### 6.3.1.2 FACS buffer

Prepare a 2% FCS/PBS solution (v/v) by adding 10 mL of heat‐inactivated FCS to 490 mL of 1X PBS.

##### 6.3.1.3 Antibody staining mix

Stain cells against extracellular molecules by adding 1–5 µL of each selected antibody per 1 × 10^6^ cells in a total volume of 150 µL.

The total volume should be prepared by adding 50 µL of Brilliant stain buffer, the antibodies listed in Table 19, according to their indicated dilution, to 76.5 µL of FACS buffer per sample.

**Table 19:** Dilution of antibodies used for flow cytometry.

Fluorophore	Antigen	Clone	Catalog no.	Company	Dilution
APC‐R700	HLA‐DR	G46‐6	565127	BD	1:150
BUV661	CD3	HIT3a	741596	BD	1:150
BUV737	CD56	NCAM16.2	612766	BD	1:150
APC‐Cy7	CD66	G10F5	305126	BioLegend	1:150
BV510	CD19	HIB19	302242	BioLegend	1:150
BV605	CD5	L17F12	364020	BioLegend	1:150
BV650	CD1c	L161	331542	BioLegend	1:150
BV750	CD45	2D1	368542	BioLegend	1:150
APC	CD207	10E2	352206	BioLegend	1:150
PerCP/Cyanine5.5	CD141	M80	344112	BioLegend	1:150
UV	Zombie		432107	BioLegend	1:150
BUV395	CD123	7G3	564195	BD	1.5:150
VioBlue	CD326(Epcam)	HEA‐125	130‐113‐266	Miltenyi Biotec	3:150
BV711	CD45RA	HI100	304138	BioLegend	3:150
AF647	AXL	108724	FAB154R‐100UG	R&D system	5:150

#### 6.3.2 Isolation and preparation of single‐cell suspensions from oral mucosal tissues

In the section **5** **Preparation of single‐cell suspensions of human gingiva**, we provide a detailed protocol of how to isolate the gingiva from the human oral cavity, followed by instructions on obtaining single‐cell suspensions from these tissues for further analysis by flow cytometry.

#### 6.3.3 Antibody staining of single‐cell suspensions from oral mucosal tissues for flow cytometry

1. Transfer the isolated single‐cell suspension to a 15 mL tube and store it at 4°C or on ice until the staining mix is prepared. The cells isolated from a 3 × 2 mm tissue sample are sufficient to analyze DC subpopulations as shown in this protocol.
2. Prepare 150 µL of antibody staining mix per sample in a 1.7 mL Eppendorf tube, as described in the antibody staining mix, (please see section **5** **Preparation of single‐cell suspensions of human gingiva**).
3. Add 150 µL of antibody staining mix to each sample and incubate for 30 min at 4°C.
4. Wash the cells by adding 2 mL FACS buffer and pipetting up and down.
5. Centrifuge the tubes at 314 × *g* for 5 min at 4°C.
6. Discard the supernatant and remove any residual liquid.
7. Resuspend the cells in 70 µL fixation/permeabilization solution (found in the BD Cytofix/Cytoperm kit).
8. The cells can be acquired directly or kept in the dark at 4°C until acquired on the flow cytometer.

### 6.4 Data analysis

**Figure 6:**
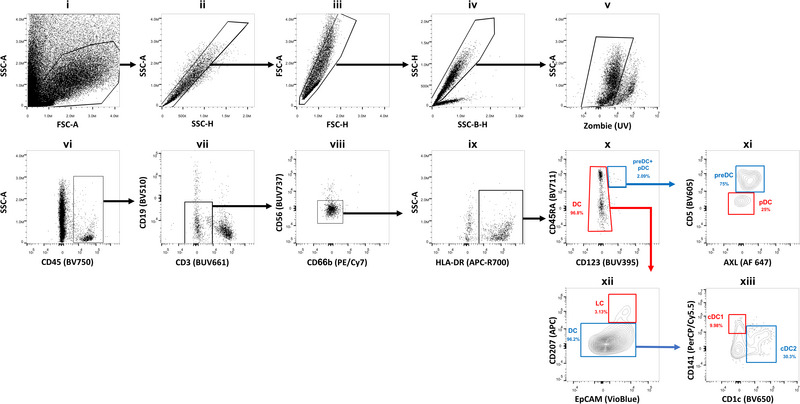
Gating strategy for the characterization of dendritic cell subsets in human gingiva. (i) Cells are pregated according to their size and granularity (FSC‐A/SSC‐A) to exclude debris and dead cells. (ii, iii) Doublets are excluded by gating on the FSC and SSC area (A) and height (H). (iv) Red blood cells are excluded by using SSC‐B‐H and SSC‐H. (v) Dead cells are excluded using Zombie staining. (vi) CD45^+^ hematopoietic cells are selected. (vii) CD3^+^ T cells and CD19^+^ B cells are excluded. (viii) CD66b^+^ neutrophils and CD56^+^ NK cells are excluded. (ix) HLA‐DR^+^ cells are selected. (x) CD123^+^, CD45RA^+^, and CD123‐CD45RA^+∖−^ cells are further gated, which represent the pre‐DC, pDC, and DC subpopulations, respectively. (xi) CD5^+^AXL^+^ cells represent the pre‐DC subpopulation and CD5‐AXL‐ cells constitute the pDC subpopulation. (xii) LC cells are isolated from other DC cells by their expression of both CD207 and Epcam. (xiii) Conventional DC (cDC) are divided into cDC1 and cDC2 according to their CD141 and CD1c expression. While cDC1 are positive for CD141 and negative for CD1c, cDC2 are positive for CD1a and negative for CD141. Data acquisition was performed on a Cytek Aurora Flow Cytometry System and data was analyzed using FlowJo V10.8.1 software.

Data acquisition was performed on a Cytek Aurora Flow Cytometer (Cytek Biosciences) equipped with 355, 405, 488, 561, and 640 nm lasers. Data were analyzed using FlowJo software. The gating strategy provided in Fig. 6 shows an example of the identification of the four DC subsets in the gingiva and can be applied to other oral tissues.

### 6.5 Pitfalls

1. Please note that antibody concentrations need to be calibrated and adjusted to each flow cytometer and its lasers. The suggested antibody dilutions in Table 19 are optimized for the Cytek Aurora Flow Cytometry System.
2. Varying DC subset distribution in the different oral mucosal tissues may be observed as every tissue has its unique leukocyte compartment.

### 6.6 Top tricks

1. To analyze subpopulations, we recommend harvesting a sample of at least 3 × 2 mm (∼15 mg) per sample to ensure a proper flow cytometry analysis.
2. To avoid cell loss during acquisition, increase the volume of FACS buffer used to resuspend the cells before acquiring.
3. It is recommended to use a Brilliant stain buffer for the polymer antibody staining mix to eliminate nonspecific reactivity between the polymer‐based fluorochromes, as this can result in undercompensation of the data.
4. For a correct setup of the flow cytometer, single‐stained cells or beads should be used. Further, unstained cells (FMO controls) or isotype controls for the marker of interest should be used.

### 6.7 Summary of the phenotype

The overall phenotype of DC and LC covered by the markers included in the antibody panel is detailed in Table 20.

**Table 20:** Summary of marker expression on analyzed cell populations.

Population	Marker negative	Marker positive
Pre‐DC	CD3, CD19, CD56, CD66b	CD45, HLA‐DR, CD123, CD45RA, AXL, CD5
cDC2	CD3, CD19, CD56, CD66b, CD123, CD207 (Langerin), Epcam, CD141	CD45, HLA‐DR, CD45RA^+/−^, CD1c, HLA‐DR
cDC1	CD3, CD19, CD56, CD66b, CD123, CD207 (Langerin), Epcam, CD1c	CD45, HLA‐DR, CD45RA^+/−^, CLEC9A, CD141, HLA‐DR
Langerhans cells	CD3, CD19, CD56, CD66b, CD123	CD45, HLA‐DR, CD45RA^+/−^, EpCam, Langerin, HLA‐DR
pDC	CD3, CD19, CD56, CD66b	CD45, HLA‐DR, CD123, CD45RA

## 7 Preparation of single‐cell suspensions from immune compartments of the human intestine

### 7.1 Introduction

The intestinal immune system can be broadly divided into intestinal inductive and effector sites. The former includes the intestinal draining mesenteric lymph nodes and the gut‐associated lymphoid tissues (GALT); the macroscopically visible Peyer's patches (PP) of the small intestine (SI) and the smaller but far more numerous isolated lymphoid follicles (ILFs) that are distributed along the whole intestine [55]. Within these sites, adaptive immune T and B cells undergo initial priming and differentiation. In contrast, the intestinal effector sites are the lamina propria (LP) and epithelium, where innate and primed adaptive immune cells localize to promote barrier integrity, protective immunity, and tolerance against food antigens and commensal microbes.

**Figure 7:**
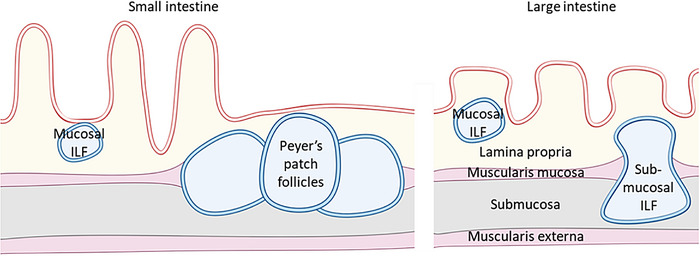
Illustration of immune compartments that can be isolated from the gut wall of small and large human intestines. The human gut wall can be processed and then peeled apart under a dissecting microscope to isolate the mucosa from the submucosa. From the mucosa, it is then possible to isolate the epithelium, lamina propria, and mucosal ILF. Small intestinal Peyer's patches can be macroscopically identified and removed from surrounding tissue, after which individual follicles can be stained and isolated using methylene blue. By staining the submucosa of the large intestine with methylene blue, individual submucosal ILF can be isolated.

Here, we describe the use of our novel protocols [56, 57], to isolate single‐cell suspensions from GALT free‐LP, PP, and ILF along the length of the human intestine (Fig. 7), which allows subsequent analysis of the major cDC subsets within these sites as exemplified in the section **8** **Flow cytometry analysis of cDC from immune compartments of the human intestine**. The protocol is amenable to multiple downstream cDC analysis, including in vitro culture as well as single‐cell transcriptomics, allowing cDC subset composition, transcription, and function to be compared across locations.

### 7.2 Materials

#### 7.2.1 Reagents

A complete list of reagents is provided in Table 21.

**Table 21:** Reagents, antibodies, chemicals, and solutions.

Reagent	Manufacturer	Ordering number
HBSS	Gibco	14170112
Methylene blue solution	Sigma‐Aldrich	03978
Collagenase D	Sigma‐Aldrich	11088858001
Liberase TM	Sigma‐Aldrich	5401119001
DNAse I	Roche	10104159001
RPMI	HyClone	SH30027.01
FCS (heat‐inactivated before use)	Atlanda Biologicals	S11150
Pen‐Strep‐Glutamine (100×)	HyClone	SV30082.01
PBS (10× stock)	Rockland	MB‐008
0.5 M EDTA (pH 8.0)	Hoefer	GR123‐100
1 M dithiothreitol	Merck	646563

#### 7.2.2 Equipment

Necessary equipment are listed in Table 22.

**Table 22:** Necessary equipment.

Equipment	Company	Ordering number
Class 2 biological safety cabinet	Labogene	Mars
Petri dishes	Fisher Scientific	12694785
Dumont #7 curved forceps	Agnthos	11274‐20
Curved dissecting scissors	Agnthos	03‐029‐115
No. 22 scalpel blades	Heinz Herenz Medizinalbedarf	1110922
No. 4 scalpel handles	Heinz Herenz Medizinalbedarf	1110705
Dissecting microscope with transmitted light source	VWR	630‐1943
Biopsy punches	WPI	WP3030
Incubator	Thermo Scientific	3111
50 mL tubes	Sarstedt	62.547.004
15 mL tubes	Sarstedt	62.554.502
1.5 mL tubes	Sarstedt	72.690.001
Thermoshaker	CarlRoth	MHR 13
100 µm filter	Corning	431752
Centrifuge	Thermo Scientific	75‐257‐406
Microcentrifuge	VWR	521‐1651
Pasteur pipettes	Fisher Scientific	07‐201‐926

### 7.3 Step‐by‐step sample preparation

#### 7.3.1 Preparation of solutions

Cold HBSS (HBSS with 1% penicillin/streptomycin, 16 mM Hepes):

1. 437 mL autoclaved Mili Q water
2. 50 mL HBSS 10X without Ca^2+^/Mg^+^
3. 8 mL 1M Hepes
4. 5 mL penicillin/streptomycin

Cold R5 (RPMI 1640 with 1% p/s, 5% heat‐inactivated FCS):

1. 475 mL RPMI 1640 (with l‐Glut)
2. 25 mL heat‐inactivated FCS
3. 5 mL penicillin/streptomycin

DTT buffer (R5 with 4 mM DTT):

1. 50 mL warm R5
2. 200 µL of 1M stock DTT (4 µL/mL)

EDTA buffer (HBSS with 5 mM EDTA):

1. 50 mL warm HBSS
2. 500 µL of 0.5 M stock (10 µL/mL)

Digest buffer (R5 with 60 µg/mL liberase TM or 100 µg/mL collagenase D, and 0.15 mg/mL DNAse):

1. 10 mL warm R5
2. 240 µL of 2.5 mg/mL TM stock (24 µL/mL)
3. Alternatively, 200 µL of 5 mg/mL collagenase D (20 µL/mL)
4. 150 µL of 10 mg/mL DNase I stock

Methylene blue (PBS with 0.1% methylene blue):

1. 1 mL of 1.5% methylene blue
2. 14 mL PBS

Methylene blue rinse buffer (PBS with 5 mM EDTA):

1. 50 mL warm PBS
2. 500 µL of 0.5 M EDTA

#### 7.3.2 Preparation of intestinal resection sample

 

1. Wash resection sample in cold R5
2. Place sample in Petri dish with cold R5

Isolate muscularis externa:

1. Cut away muscularis externa in strips using forceps and scissors. If digesting the muscularis externa, store on ice until digest step.

Wash off mucus:

1. Cut the remaining tissue into 5 cm^2^ sections for ease of processing.
2. For small intestinal tissue only, gently brush the villi with a scalpel under a dissecting microscope to remove large pieces of mucus.
3. Incubate each 5 cm^2^ tissue piece in a 50 mL tube with 10 mL DTT buffer in a 37°C shaking incubator for 10 min, ×2.
4. Place sample in a Petri dish lid with 5 mL cold R5.

Isolate submucosa:

1. Carefully trim away visible submucosa with scissors. If digesting the submucosa, store on ice until digest step.

Isolating lymphoid patches:

1. PP and (in some samples) caecal patches can be identified by (1) the presence of multiple visible follicles in the gut wall, (2) the presence of black dots in the submucosa, (3) the inability to peel mucosa from muscularis mucosa surrounding PP. These can be cut away from the surrounding tissue using a scalpel, and individual follicles can be isolated using a biopsy punch.

Separate mucosa from submucosa:

1. Using two sharp forceps, carefully and slowly peel mucosa from muscularis mucosa. A dissecting microscope is necessary to prevent tissue damage and tissue contamination during this step. Store separated tissues in R5 on ice.

Isolate submucosal ILF:

1. Wash muscularis mucosa/submucosa with PBS
2. Stain muscularis mucosa/submucosa in warm 0.1% methylene blue for 2 min
3. Wash in warm PBS
4. Wash in 10 mL warm MB rinse buffer three times for 2–5 min each, until buffer stays clear
5. Extract GALT from submucosa under dissecting microscope with transmitted light source in cold R5, using biopsy punch or scalpel. Store submucosal ILF on ice until digest step.

Isolate epithelium and intraepithelial lymphocytes:

1. Wash mucosa with HBSS
2. Shake 5 cm^2^ pieces of mucosa in 10 mL EDTA buffer in 37°c incubator for 10 min and repeat until no epithelium remains visible in suspension (usually ×3 for colon, ×4 for SI). If digesting the epithelium, store it on ice until digest step.

Isolate LP and mucosal ILF:

1. Check LP for mucosal ILF under a dissecting microscope with the transmitted light source. Remove mucosal ILF using a biopsy punch or scalpel. It may be necessary to gently move apart villi with a scalpel blade to visualize small ILF. Mucosal ILF are most commonly found in the terminal ileum and rectum.

Enzymatic digestion:

1. Using scalpels, cut the remaining LP and any other compartments into 2–3 mm^2^ pieces
2. Transfer fragments into 50 mL tube with 10 mL digest buffer per 5–7 cm^2^ tissue section and place in 37°C shaking incubator at 370 RPM for 45 min.
3. Dissociate digested tissue with a Pasteur pipette (squeeze 50 times). The buffer should become cloudy and no solid tissue should remain.
4. Pass the suspension through a 100 µm filter and dilute with 30 mL cold R5, then store on ice
5. For ILF, begin by cutting each ILF in two using a scalpel. Add 700 µL digest buffer in 1.5 mL tubes and incubate for 45 min at 37°C on a thermoshaker, at 800 rpm. ILF can be digested individually or in bulk.
6. Dissociate ILF by scraping thoroughly with the flat end of the syringe handle through a 100 µm filter
7. Centrifuge cells at 400*g* for 7 min.
8. Resuspend cells in cold R5.
9. Count cells.

### 7.4 Data analysis

Examples of flow cytometric data analysis of intestinal cDC using the described single cell preparation are discussed in detail in the section **8** **Flow cytometry analysis of cDC from immune compartments of the human intestine**.

### 7.5 Pitfalls

Problem: Low cell viability in digested samples

Potential solutions:

Titrate the digest enzyme concentration and increase mechanical disruption with the Pasteur pipette.

Digest for a shorter time and save the cell solution on ice while further digesting any remaining solids with fresh digest buffer.

Ensure tissues are kept on ice whenever possible.

Problem: Low cellular yield in digested samples

Potential solutions:

Increase digest enzyme concentration and mechanical disruption with Pasteur pipette.

Increase digest time.

Ensure submucosa was fully removed from mucosa under dissecting microscope.

### 7.6 Top tricks

At step 8, thorough removal of attached submucosa is necessary to allow methylene blue to penetrate and stain submucosal ILF

Avoid treating the mucosa with EDTA before peeling away from the submucosa, as the mucosa will become too fragile to separate.

Peeling the mucosa from the muscularis mucosa/submucosa is an acquired skill, which might seem impossible at first, but it becomes easy with practice. Start peeling from a corner, holding mucosa in left forceps and submucosa in right. Use sharp forceps with a curve. Try cutting tissue into 1 cm wide strips, and repeat DTT wash if no corners can be found initially. Peel in a continuous line, recovering once the line is lost is very difficult. Use only the tips of forceps to peel around holes left by ILF.

DNAse might not be required for digestion of the LP or ILF and could be left out if DNAse‐sensitive single‐cell sequencing is to be used without multiple washing steps.

## 8 Flow cytometry analysis of cDC from immune compartments of the human intestine

### 8.1 Introduction

cDC1 and cDC2 are found throughout the intestine in both intestinal inductive and effector sites and play a central role in initiating tolerogenic as well as immunogenic responses [58]. Studies in mice have demonstrated key nonredundant roles for intestinal cDC1 and cDC2 in intestinal immune homeostasis. For example, cDC2 appear to be important for intestinal Th17 homeostasis [59], for the generation of IgA and IgG antibody responses to flagellin [60], and for the induction of Th2 responses to intestinal parasites [61]. In contrast, cDC1 are required for the generation of the small intestinal intraepithelial lymphocyte and Th1 compartment [62], and for driving tolerogenic CD8^+^ T‐cell responses to epithelial‐derived self‐antigen [63]. There is also mounting evidence in mice that local environment factors imprint cDC with distinct phenotypes and functions along the length of the intestine and that functionally distinct cDC subsets reside in mouse PP and ILF [64, 65]. Despite the above, our knowledge of cDC heterogeneity and function in distinct immune compartments of the human intestine remain limited.

Here we described a flow‐cytometry based staining protocol for the identification of human intestinal cDC1 and cDC2 from intestinal tissue digestions generated following the protocols outlined in **7** **Preparation of single‐cell suspensions from immune compartments of the human intestine**.

### 8.2 Materials

#### 8.2.1 Reagents

A complete list of reagents is provided in Table 23.

**Table 23:** Reagents, antibodies, chemicals, and solutions.

Reagent	Manufacturer	Ordering number
PBS (10× stock)	Rockland	MB‐008
Bovine serum albumin (BSA) (30% w/v in 0.85% NaCl)	Sigma‐Aldrich	A7284
Sodium azide (10% w/v solution)	Teknova	S0209

#### 8.2.2 Equipment

Necessary equipment are listed in Table 24 and used antibodies in Table 25.

**Table 24:** Necessary equipment.

Equipment	Company	Ordering number
Class 2 biological safety cabinet	Labogene	Mars
50 mL tubes	Sarstedt	62.547.004
15 mL tubes	Sarstedt	62.554.502
1.5 mL tubes	Sarstedt	72.690.001
Centrifuge	Thermo Scientific	75‐257‐406
Microcentrifuge	VWR	521‐1651
Pasteur pipettes	Fisher Scientific	07‐201‐926

**Table 25:** Used antibodies.

Antibodies	Company	Ordering number	Dilution
BUV395 anti‐human CD45 clone HI30	BD	563791	1:100
BUV737 anti‐human CD38 clone HB7	BD	564686	1:50
BV786 anti‐human CD1c clone F10/21A3	BD	742750	3:100
BV711 anti‐human CD141 clone 1A4	BD	563155	1:50
PECF594 anti‐human CD19 clone HIB19	BD	562294	1:20
PECF594 anti‐human CD3 clone UCHT1	BD	562280	1:20
BB700 anti‐human CD123 clone 7G3	BD	566482	1:25
BV650 anti‐human CD163 clone GHI/61	BD	563888	1:50
BV605 anti‐human CD103 clone Ber‐ACT8	BioLegend	350218	3:100
BV421 anti‐human CD14 clone MφP9	BD	563743	1:50
AF700 anti‐human HLA‐DR clone G46‐6	BD	560743	3:100
Sytox green viability dye	Thermo Fisher	S7020	3:10.000

### 8.3 Step‐by‐step sample preparation

#### 8.3.1 Preparation of solutions

FACS buffer:

1. 500 mL PBS
2. 5 g bovine serum albumin
3. 0.25 g sodium azide

#### 8.3.2 Flow cytometry

In the section **7** **Preparation of single‐cell suspensions from immune compartments of the human intestine**, we provide a detailed protocol on how to isolate cells from human intestinal tissue for analysis by flow cytometry.

1. Resuspend up to 2 million cells per sample in 50 µL FACS buffer containing 4% mouse serum.
2. Incubate on ice for 15 min.
3. Add antibodies diluted to 50 µL in FACS buffer onto cells (see Table 25).
4. Incubate on ice for 30 min.
5. Add 900 µL FACS buffer to cells.
6. Centrifuge cells at 300 g for 5 min.
7. Discard supernatant and resuspend cells in 300 µL FACS buffer with 0.3 µL Sytox green viability dye. Note: Sytox green was used here to allow the sorting of viable cell populations. If cells are only to be analyzed by flow cytometry, fixation of cells after staining with fixable viability dyes is recommended.
8. Incubate on ice for at least 15 min and analyze cells on a flow cytometer.

### 8.4 Data analysis

**Figure 8:**
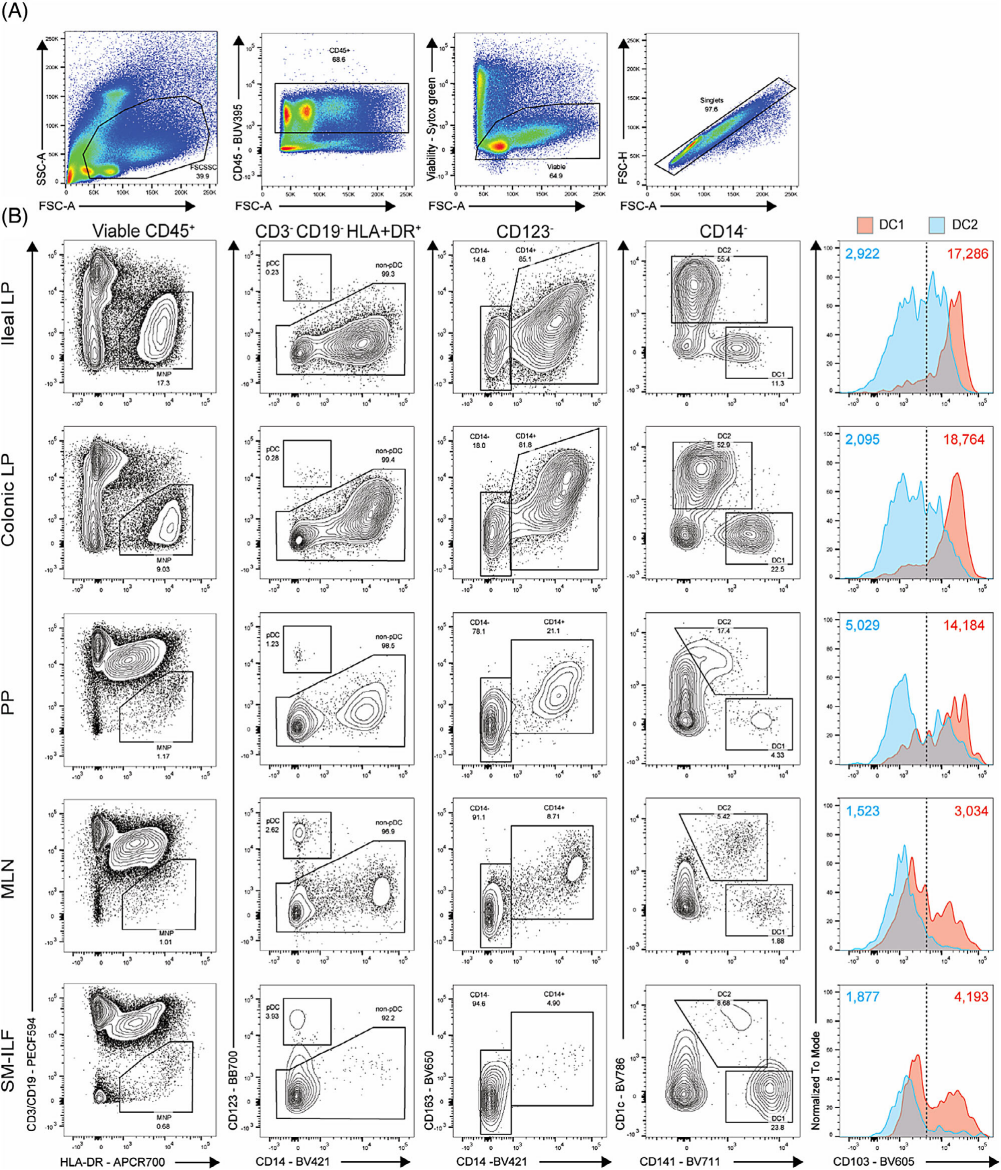
Flow cytometry analysis of cDC subsets isolated from different compartments of the human intestine, showing (A) pregating used for all compartments and (B) plasmacytoid DC and conventional DC subset gating, with slight gating variations for each compartment. Numbers in histograms represent the median fluorescence intensity of CD103 for DC1 (red) and DC2 (blue), dashed line delineates positive from negative CD103 signal. LP, lamina propria; MLN, mesenteric lymph nodes; PP, Peyer's patch; SM‐ILF, submucosal isolated lymphoid follicles. Representative data of 3–10 colorectal cancer patients per compartment, with tissue taken from unaffected areas.

Flow cytometry data was analyzed using Flowjo software (BD Life Sciences). Cells were first gated as viable, CD45^+^ single cells (Fig. 8A). The cells were then gated as CD3^−^ CD19^−^ HLADR^+^ mononuclear phagocytes and the gates were shifted slightly for each compartment analyzed (Fig. 8B). Contaminating CD123^+^ plasmacytoid DC and CD163^+^ CD14^+^ monocytes/macrophages were gated out before cDC were gated as CD141^+^ cDC1 and CD1c^+^ cDC2. Fluorescence minus‐one controls were used to check the validity of the gating strategy. CD103 expression is found to different extents on cDC subsets within different intestinal compartments, with highest expression on cDC1 in the ileal LP, and lowest expression on cDC2 from mesenteric lymph nodes and submucosal isolated lymphoid follicles (Fig. 8B).

### 8.5 Pitfalls

Problem: Low cell viability in digested samples

Potential solutions:

Titrate the digest enzyme concentration and increase mechanical disruption with the Pasteur pipette.

Digest for a shorter time and save the cell solution on ice while further digesting any remaining solids with fresh digest buffer.

Ensure tissues are kept on ice whenever possible.

Problem: Low cellular yield in digested samples

Potential solutions:

Increase digest enzyme concentration and mechanical disruption with Pasteur pipette.

Increase digest time.

Ensure submucosa was fully removed from mucosa under dissecting microscope.

### 8.6 Top tricks

Methylene blue is detected in filters excited by the red laser in flow cytometry, especially the APC channel. If this signal is too high, increase methylene blue washing steps and titrate the methylene blue staining concentration.

Expression of CD15 and CD56 were not found in the human intestinal HLA‐DR^+^ gate and so were here excluded from the ‘dump’ channel.

### 8.7 Summary of the phenotype

The overall phenotype of cDC1 and cDC2 covered by the markers included in the panel is detailed in Table 26.

**Table 26:** Summary of marker expression on analyzed cell populations.

DC subset	Phenotype
cDC1	CD45^+^ CD3^−^ CD19^−^ HLA‐DR^+^ CD123^−^ CD14^−^ CD163^−^ CD141^+^ CD1c^−^
cDC2	CD45^+^ CD3^−^ CD19^−^ HLA‐DR^+^ CD123^−^ CD14^−^ CD163^−^ CD141^−^ CD1c^+^

## 9 Preparation of single‐cell suspensions from human tumors

### 9.1 Introduction

Based on the level of immune infiltration, the tumor microenvironment (TME) can be classified as inflamed (i.e. heavily infiltrated), immune excluded (i.e. immune cells in surrounding stroma but not in tumor fields), or immune desert (i.e. absence of immune cells) [66]. Disease outcome and efficacy of immunotherapy is highly dependent on the TME infiltration status and the balance between anti‐tumor and protumor immune responses that make up this immune infiltrate. Priming and programming of the T cells that will eventually populate the TME takes place in tumor‐draining lymph nodes (TDLN). It is there that cDC that have either migrated from the tumor lesion or that reside in the TDLN and that have taken up tumor antigens, will prime antigen‐specific CD4^+^ and CD8^+^ T cells [67, 68]. Both migratory and lymph node‐resident (LNR)‐cDC, and cross‐talk between them, play key roles in the induction of effective antitumor T‐cell responses [69]. DC are not just key to the de novo induction of T‐cell responses, but also to the invigoration of exhausted T cells and their recruitment to the TME, all of which are prerequisites for effective immune checkpoint blockade [70, 71, 72, 73, 74, 75, 76].

Here, we present a protocol to isolate single‐cell suspension from tumor tissues, which can be subsequently used for flow cytometric analysis with polychromatic flow cytometry panels designed for the assessment of myeloid APC, including cDC, in clinical tumor sample‐derived single‐cell suspensions as detailed in the section **10** **Flow cytometry analysis of conventional dendritic cells in human tumors**.

### 9.2 Materials

#### 9.2.1 Reagents

A complete list of reagents is provided in Table 27.

**Table 27:** Reagents, antibodies, chemicals, and solutions.

Reagent	Manufacturer	Ordering number
RPMI 1640 medium	Lonza	BE12‐702F
Fetal calf serum	Hyclone	SV30160.03
Penicillin‐streptomycin‐glutamine	Gibco	10378016
Collagenase A	Roche	10103586001
Deoxyribonuclease I (DNAse I)	Roche	10104159001
BSA (albumin bovine fraction V)	Sigma‐Aldrich	B6917
Brilliant staining buffer	BD	563794
NH_4_Cl	Merck	101145
KHCO_3_	Merck	104854
EDTA	Merck	819040

#### 9.2.2 Equipment

Necessary equipment are listed in Table 28.

**Table 28:** Necessary equipment.

Equipment	Company	Purpose
1.5 or 2 mL reaction tube	Eppendorf	Preparation of staining mix, Live/Dead dye solution
Surgical blade (size no.22)	Swann Morton Ltd.	To scrape TDLN cut surface
Dissociation flask with magnetic stir bar	Wheaton USA	To contain TDLN cells during enzymatic digestion
Submersible magnetic stirrer	Thermo Scientific	To stir TDLN cells during enzymatic dissociation
100 µm cell strainer	FALCON	Filtering of cell suspension
50 mL conical tube	FALCON	Store and wash cells after isolation
Pipettes	Eppendorf	Washing and cell/mAb dispensing
Centrifuge	Hettich Zentrifugen	Centrifugation of cells

### 9.3 Step‐by‐step sample preparation

#### 9.3.1 Preparation of media and buffer

Complete medium: RPMI 1640 supplemented with 10% FCS, 100 µg/mL streptomycin sulfate, 100 I.E./mL sodium penicillin, 2 mM l‐glutamine (P/S/G).

Dissociation medium: RPMI 1640 supplemented with P/S/G, 0.1% DNase I, 0.14% collagenase A, and 5% FCS.

Shock buffer: Dilute 2.0 mL EDTA in 1 L H_2_O, 9.844 g NH_4_Cl, and 1.0 g KHCO_3_. To sterilize, filtrate the solution using a 0.22 µM filter.

#### 9.3.2 Tumor processing to single‐cell suspension

Tumors were sampled under written informed consent, according to protocols approved by the Amsterdam UMC institutional review board, under the supervision of a pathologist. Biopsies of at least 1 cm^3^ from surgical specimens were collected.

1. Collect tumor biopsy immediately after surgical removal in 10 mL complete medium in a 50 mL polypropylene tube. The tumor biopsies should preferably be at least 1 cm^3^. NB: cell yields can vary widely, depending on tumor type and consistency (ranging from <1 million to >50 million).
2. If further isolation and culture or functional testing of samples is planned, preferably process tumor biopsy in a sterile environment like a laminar flow hood; or keep the processed tumor biopsy as sterile as possible.
3. Cut the tumor fragment into 1–2 mm pieces with a sterile scalpel (no. 22) and sterile forceps on a sterile Petri dish on ice. Transfer the pieces to a 10 mL dissociation medium in a sterile flask with a magnetic stir bar. Rinse the petri dish with 2 × 10 mL dissociation medium and add to the flask (total volume 30 mL).
4. Place a magnet stirrer in a water bath at 37°C.
5. Put the flask on the stirrer in the water bath and switch on the stirrer; leave the suspension to stir at a gentle rotation for 45 min.
6. Switch off the stirrer and collect the medium by gently pouring it into a 50 mL conical PP tube. Resuspend the remaining fragments in another 30 mL of dissociation medium and let the suspension stir in a 37°C water bath on the magnetic stirrer for another 45 min.
7. Repeat step 6 1–2 more times (to a total of 3–4 dissociation rounds). After the last dissociation round, rinse the fragments with 30 mL complete medium, while pushing them through a cell strainer, using the blunt end of a sterile (2 mL) syringe rubber plunger.
8. Centrifuge the tubes for 5 min at 530 × *g* and 4°C.
9. Resuspend cell pellets and combine them in a 10 mL complete medium.
10. If visible erythrocyte contamination is present (i.e. the pellet is red), the erythrocytes should be lysed by incubating the cells at 4°C for 10 min with 10 mL shock buffer.
11. After the 10 min, add 40 mL of complete medium and centrifuge the cells for 5 min at 530 × *g* and 4°C.
12. Resuspend pellet in 3 mL complete medium.
13. Count cells and either cryostore or immediately use for flow cytometry analysis as detailed in the section **10** **Flow cytometry analysis of conventional dendritic cells in human tumors**.

### 9.4 Data analysis

Examples of flow cytometric data analysis of tumor DC subsets using the described single cell suspensions are shown in detail in the section **10** **Flow cytometry analysis of conventional dendritic cells in human tumors**.

### 9.5 Pitfalls

If yields allow, tumor‐derived single‐cell suspensions can be viably cryopreserved. For best results employ a controlled‐rate freezing set‐up. Of note, single‐cell suspensions used for this report were thawed from crystored samples. Be aware that this obviously precludes analysis of an important myeloid subset, that is, granulocytes.

### 9.6 Top tricks

#### 9.6.1 Cell viability

The interval from collection to digestion significantly impacts cell viability. Minimizing this timeframe is crucial. Moreover, the tissue should be maintained on ice consistently until digestion.

#### 9.6.2 Prevention of loss of membrane markers during enzymatic digestion

Membrane proteins may be cleaved during DNAse/Collagenase digestion. We have found that the addition of 5% FCS to the Dissociation medium could largely prevent this [77].

#### 9.6.3 Culture of tumor single‐cell suspensions

The obtained suspensions can also be used for in vitro culture and functional assessment of immune modulation. We have successfully studied cDC differentiation and activation as well as T‐cell activation, through exposure to TLR ligands, small molecules, immune checkpoint inhibitors, innate effector cells, or immune modulatory viral vectors in cultures that were maintained for up to 5 days. Although myeloid APC/cDC phenotypes changed, they could still reliably be assessed by FACS analysis at day 5 (and compared with a medium control condition).

#### 9.6.4 Alternative dissociation methods

Using the Miltenyi gentleMACS octo dissociator, eight tumor samples may simultaneously be dissociated, requiring only one round of dissociation, thus allowing for the processing of multiple tumor samples in considerably less time. NB: results may vary between tumor/tissue types; specialized kits are available for optimized yields of either tumor cells or infiltrating immune cells.

## 10 Flow cytometry analysis of conventional dendritic cells in human tumors

### 10.1 Introduction

cDC have been classified as cDC1 or cDC2, each with different transcriptional profiles directing their differentiation and functionality [78]. cDC1 has been identified as a subset with a particular ability of cross‐priming CD8^+^ effector cells [79], although in human lymph nodes this ability appears to be shared by all residing cDC subsets [80]. More recently in human peripheral blood a CD14^+^CD163^+^ proinflammatory cDC3 subset has been identified that shares characteristics with cDC2, but differentiates along a separate lineage from cDC1 and cDC2 both under steady‐state and inflammatory conditions [10, 78, 81].

Low frequencies of migratory cDC subsets in the sentinel lymph node (SLN) have been related to subsequent loco‐regional metastasis, consistent with the creation of premetastatic niches by suppressing the migration of antigen‐carrying cDC from the tumor to the TDLN [82]. This is in line with reported findings from mouse models by Binnewies et al. [83], showing that cDC2 migration from the tumor to TDLN was constrained by tumor‐associated regulatory T cells (T_reg_ cells), resulting in suboptimal priming of helper T cells and their failure to migrate to the tumor to support an anti‐tumor immune response. Similarly, we found cDC2 migrating from the dermis to facilitate effective PD‐1 and TIM3 inhibition in a spontaneous melanoma mouse model [84]. Flow cytometry‐based studies from our lab in single‐cell suspensions from human cancer samples have lent further support to the importance of DC in disease outcomes. In cervical adenocarcinoma, we found that recruitment of CD8^+^ T cells to the tumor depended on the rate of cDC1‐like cells in the TME, which was inversely correlated with a β‐catenin response signature and was associated with improved overall survival in patients [85], echoing previous findings of Spranger, Luke et al. [76, 86]. In melanoma, we found that decreased frequencies of tumor‐derived migratory cDC in the SLN preceded the hampered activation of the LNR‐cDC and that these events correlated with increased Breslow thickness of the primary lesion and to metastatic burden in the LN, respectively [82]. Altogether, these observations stress the vital role of tumor‐associated DC in mounting effective antitumor immunity. In line with this notion, their importance in effective immune checkpoint blockade has also emerged.

Oh et al. [74] showed that rather than PD‐L1 expressed on the tumor cell surface, PD‐L1 expressed by tumor‐infiltrating and cross‐presenting DC was decisive in PD‐1 blockade efficacy. Similarly, Garris et al. [87] demonstrated that effective (re‐)activation of antitumor T cells by PD‐1 blockade involved T‐cell/DC crosstalk and was licensed by IFN‐γ and IL‐12. This is all the more remarkable since macrophages are far more frequent than DC in tumors, and may be due to the fact that DC express high CD80 levels. CD80 interacts with PD‐L1 in‐cis [72], resulting in a block of PD‐1 binding to PD‐L1 but conserving CD80 co‐stimulatory activity through interactions with CD28 on progenitor‐exhausted or stem‐cell‐like T cells. Indeed, CD28 co‐stimulation, provided by DC in specialized niches in the TME, has been pinpointed as vital in the reversal of exhaustion of tumor‐infiltrating T cells and the licensing of PD‐1 blockade [88].

The above observations clearly stress the need to study and analyze DC in the context of the TME. A major challenge in this respect is the general phenotypic plasticity of myeloid cells, including DC. In general, DC have a propensity to take cues from their tissue environment and adjust transcriptional programs leading to phenotypic changes accordingly [89, 90]. Classically, human cDC may be identified by their expression of high levels of CD11c and HLA‐DR, either in conjunction with CD1c and/or CD141, denoting them as cDC1 or cDC2, respectively, or with CD1a, identifying them as skin‐derived migratory cDC or as monocyte‐derived DC. We and others have found that cDC under tumor‐imposed immune suppressive conditions can trans‐differentiate and acquire macrophage‐like traits, complicating their unequivocal identification as DC [91]. In addition, cDC precursor differentiation may be diverted to a macrophage‐like pathway. Indeed, in our experience, in human tumors, the vast majority of mononuclear myeloid cells (typically 90–99%) are M2‐macrophage‐like cells, characterized by CD14 expression often in conjunction with CD163, PD‐L1, DC‐SIGN, and CD141 [91, 92]. These are distinct from proinflammatory CD163^+^CD14^+^ cDC3, recently reported in oropharyngeal carcinoma [93], in that they are CD1c^−^ and express pro‐tumorigenic, proangiogenic and immune suppressive factors [92]. Recent findings show that the human precursor origins of these cells can be further delineated by the expression of CD88 and/or CD89, which, unlike cDC3, designates them as monocytic in origin [10]. Monocytes are recruited in large numbers to growing tumors and under immune suppressive conditions prevailing in the TME mostly differentiate into M2‐like macrophages with the above‐described typical phenotype [91, 94]. Indeed, exposure of monocytes to tumor‐derived supernatants in vitro results in their differentiation into M2‐like macrophages with the same phenotypic profile [91, 95]. Importantly, this also offers therapeutic options, as these monocyte‐derived macrophages may be converted into activated CD1c^+^CD1a^+^ MoDC through immune modulation [95, 96]. Indeed, MoDC can even acquire cDC1‐like properties and play a major role in directing antitumor immunity and facilitating PD‐1 blockade [97, 98].

Here, we present some representative data obtained with polychromatic flow cytometry panels designed for the assessment of myeloid APC, including cDC, in clinical tumor sample‐derived single‐cell suspensions.

### 10.2 Materials

#### 10.2.1 Reagents

A complete list of reagents is provided in Table 29 and used antibodies in Table 30.

**Table 29:** Reagents, chemicals, and solutions.

Reagent	Manufacturer	Ordering number
BSA (albumin bovine fraction V)	Sigma‐Aldrich	B6917
Brilliant staining buffer	BD	563794
NH_4_Cl	Merck	101145
KHCO_3_	Merck	104854
EDTA	Merck	819040

**Table 30:** Reagents and antibodies use for flow cytometric analysis.

Specificity	Fluorochrome	Clone	Manufacturer	Catalog #	Dilution
CD1a	PE	5c3 HI149	BD	555807	1:50
CD14	FITC	SK3	BD	345768	1:100
CD14	PerCP‐Cy5.5	MΦP9	BD	562692	1:20
CD1c	PE‐Cy7	L161	Sony	2257580	1:100
CD86	PE	2331(FUN‐1)	BD Pharmingen	555658	1:50
CD86	FITC	2331 (FUN‐1)	BD	555657	1:100
CD141	FITC	AD5‐14H12	Miltenyi Biotec	130‐090‐513	1:50
CD19	PE‐CF594	HIB19	BD	562294	1:100
CD80	FITC	L307.4	BD	557226	1:50
CD83	FITC	HB15a	Beckman Coulter	PN IM2410U	1:50
CD83	PE‐CF594	HB15e	BD	562631	1:25
CD163	PE	GHI/61	Sony	2268030	1:75
PD‐L1	FITC	MIH2	BioLegend	393606	1:25
7‐AAD			Sigma	A9400‐1MG	3 µl/tube
CD11c	APC	S‐HCL‐3	BD	333144	1:100
CD11c	APC‐CY7	Bu15	BioLegend	337218	1:50
CD45	AF700	HI30	BioLegend	304024	1:20
HLA‐DR	APC	L243	BD	347403	1:100
FVD (live/dead)	eFluor780		eBioscience	65‐0865‐14	1:1000
EpCAM	BV421	EBA‐1	BD	563180	1:100
CD163	BV421	GHI/61	BD	562643	1:75
CD1a	BV510	HI149	BD	563482	1:25
CD40	BV421	5C3	BD	563396	1:25
CD40	BV711	5C3	BioLegend	334334	1:50
CD80	BV650	L307.4	BD	564158	1:50
CD88	BV711	D53‐1473	BD	742319	1:50
CD89	BV421	A59	BD	744374	1:50
CD89	BV786	A59	BD	744379	1:50
CD141	BV711	1A4	BD	563155	1:50
TIM3	BV421	F38‐2E2	BioLegend	345008	1:20
PD‐L2	BV711	MIH18	BD	564258	1:25
PD‐L1	BV786	MIH1	BD	563739	1:25
HLA‐DR	BV786	L243	BioLegend	307642	1:200

#### 10.2.2 Equipment

Necessary equipment are listed in Table 31.

**Table 31:** Necessary equipment.

Equipment	Company	Purpose
1.5 or 2 Ml reaction tube	Eppendorf	Preparation of staining mix, Live/Dead dye solution
5 mL FACS tubes	FALCON	For cell staining and analysis
50 mL conical tube	FALCON	Store and wash cells after isolation
Pipettes	Eppendorf	Washing and cell/mAb dispensing
Centrifuge	Hettich Zentrifugen	Centrifugation of cells
LSRFortessa X‐20	BD	Flow cytometry analysis

### 10.3 Step‐by‐step sample preparation

#### 10.3.1 Preparation of media and buffer

FACS staining buffer: PBS supplemented with 0.1% BSA and 0.02% NaN_3_.

Live/Dead dye solution: dilute Fixable Viability Dye eFluor 780 1:1000 in PBS.

#### 10.3.2 Flow cytometry analysis

In the section **9** **Preparation of single‐cell suspensions from human tumors**, we provide a detailed protocol on how to isolate cells from different human tumors for analysis by flow cytometry.

1. Start staining for flow cytometry analysis with 5 × 10^5^ cells per tube and add 2 mL FACS staining buffer.
2. Centrifuge at 530 × *g*, 5 min, 4°C.
3. Discard sup and resuspend pellet in 2 mL FACS staining buffer per FACS tube.
4. Centrifuge at 530 × *g*, 5 min, 4°C.
5. If the desired staining panel comprise FVD eFluor780, resuspend cell pellets in Live/dead dye solution, incubate in the dark at RT for 5 min, and add 2 mL FACS staining buffer to wash. NB: Skip this step if your staining panel contains 7AAD.
6. Centrifuge at 530 × *g*, 5 min, 4°C.
7. Discard the supernatant and resuspend pellet in 100 µL of antibody staining mix containing the correct final dilution of antibodies (e.g. see Table 32) in FACS staining buffer 1:1 diluted with Brilliant staining buffer.
8. Incubate for 30 min at 4°C in the dark.
9. Wash cells using FACS staining buffer, and centrifuge at 530 × *g*, 5 min, 4°C.
10. Discard sup and resuspend cells in 200–300 µL of FACS staining buffer and keep in the dark at 4°C until data acquisition on a BD LSRFortessa X‐20. When using 7‐AAD as live/dead marker, add 5 min prior to measurement (keep in the dark at 4°C).

**Table 32:** Staining workflow for human tumor cDC panel.

	Marker	Fluorochrome	Dilution	Diluent	Incubation (min/temp)
**1**	FVD^a^	eFluor780	1:10,000	PBS	5’/4°C
**2**	Wash (staining buffer)				
**3**	Backbone markers:				
	CD11c	APC	1:100	FACS Staining buffer:Brilliant stain buffer (1:1)	30’/4°C
	CD11c	APC‐CY7	1:100		
	CD14	PerCP‐Cy5.5	1:20		
	CD14	FITC	1:100		
	CD1a	PE	1:100		
	CD1c	PE‐Cy7	1:100		
	CD45	AF700	1:20		
	(CD19	PE‐CF594	1:100)		
	HLA‐DR	APC	1:100		
	HLA‐DR	BV786	1:200		
	Additional markers:				
	CD40	BV711	1:50		
	CD80	FITC	1:50		
	CD80	BV650	1:50		
	CD83	FITC	1:50		
	CD83	PE‐CF594	1:25		
	CD86	PE	1:50		
	CD88	BV711	1:50		
	CD89	BV421	1:50		
	CD89	BV786	1:50		
	CD141	FITC	1:50		
	CD141	BV711	1:50		
	CD163	PE	1:75		
	CD163	BV421	1:75		
	CD40	BV421	1:25		
	TIM3	BV421	1:20		
	PD‐L2	BV711	1:25		
	PD‐L1	BV786	1:25		
	PD‐L1	FITC	1:25		
	HLA‐DR	BV786	1:200		
**4**	Wash (staining buffer)				
**5**	Resuspend cells in 200–300 µL of staining buffer and keep in the dark at 4°C until acquisition				

^a^May be replaced by 7‐AAD.

### 10.4 Data analysis

**Figure 9:**
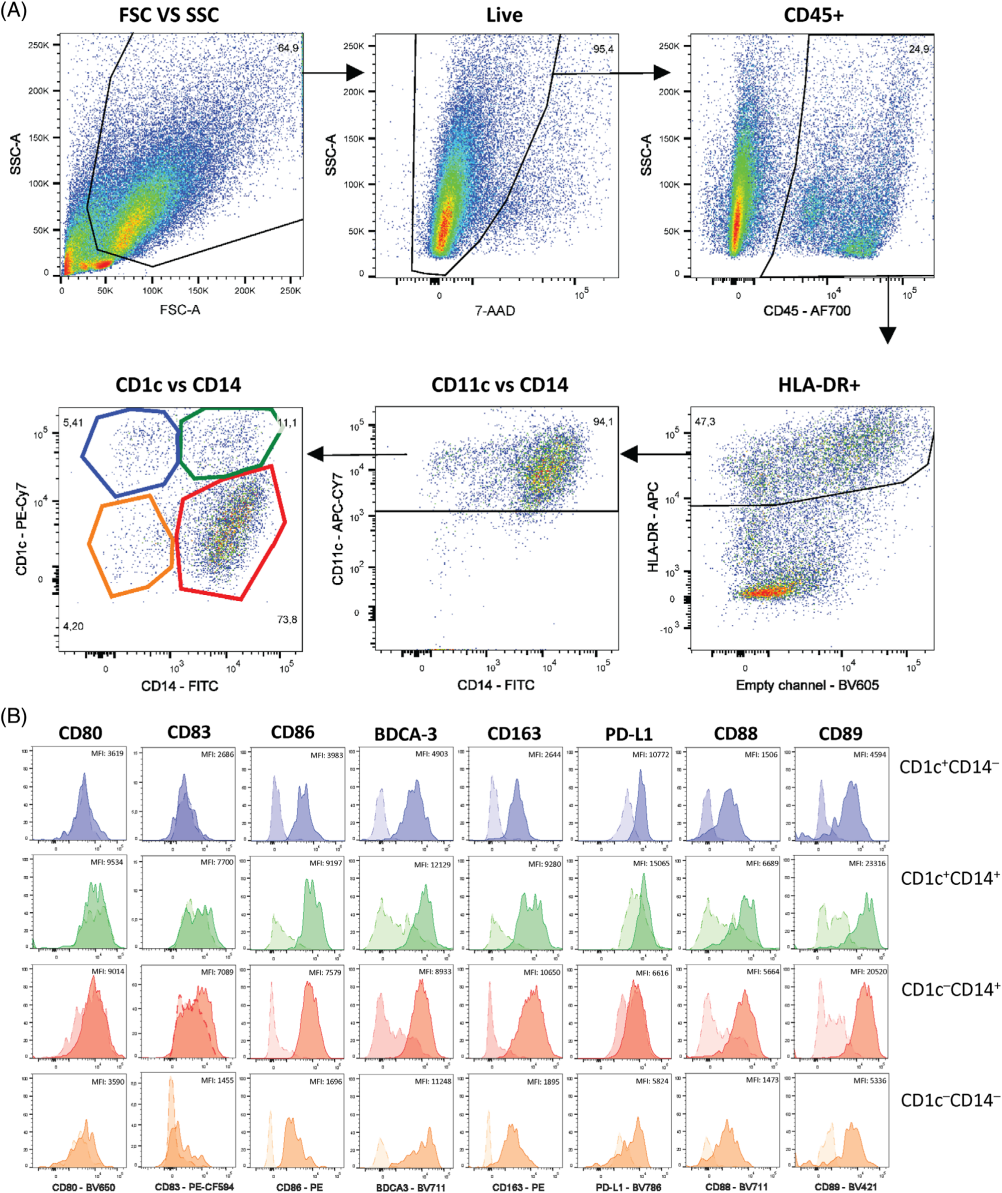
cDC gating strategy in the flow cytometric analysis of a single‐cell suspension derived from a human colorectal primary carcinoma. (A) Gating strategy for the quantitation and phenotypic analysis of myeloid APC/cDC subsets among live CD45^+^ leukocytes. (B) Expression of subset‐defining and co‐stimulatory markers as well as PD‐L1 on the identified APC/cDC subsets. Histogram overlays with fluorescence‐minus‐one controls are shown.

**Figure 10:**
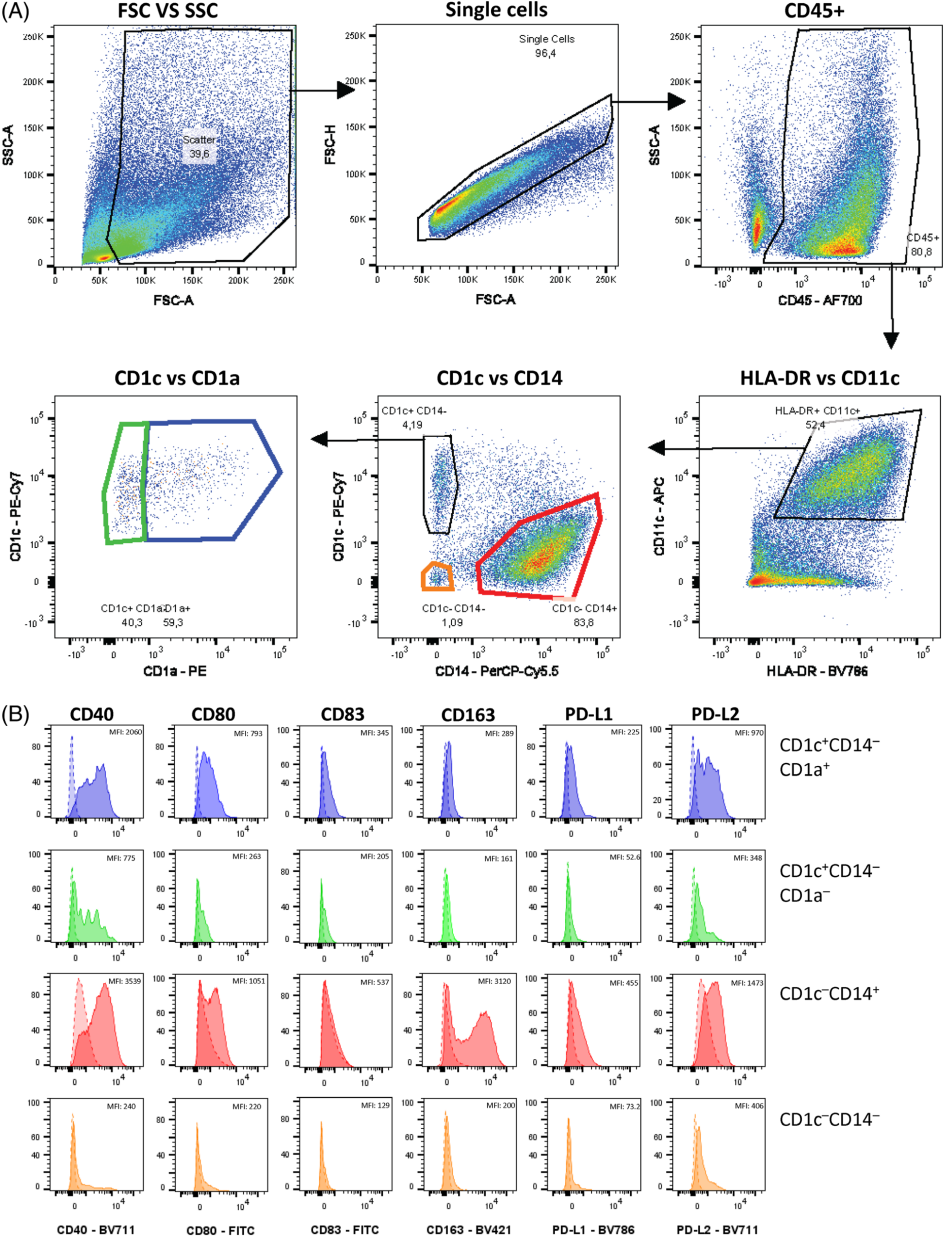
cDC gating strategy in the flow cytometric analysis of a single‐cell suspension derived from non‐small‐cell lung carcinoma. (A) Gating strategy for the quantitation and phenotypic analysis of myeloid APC/cDC subsets among CD45^+^ leukocytes. (B) Expression of subset‐defining and co‐stimulatory markers as well as PD‐L1 on the identified APC/cDC subsets. Histogram overlays with fluorescence‐minus‐one controls are shown.

**Figure 11:**
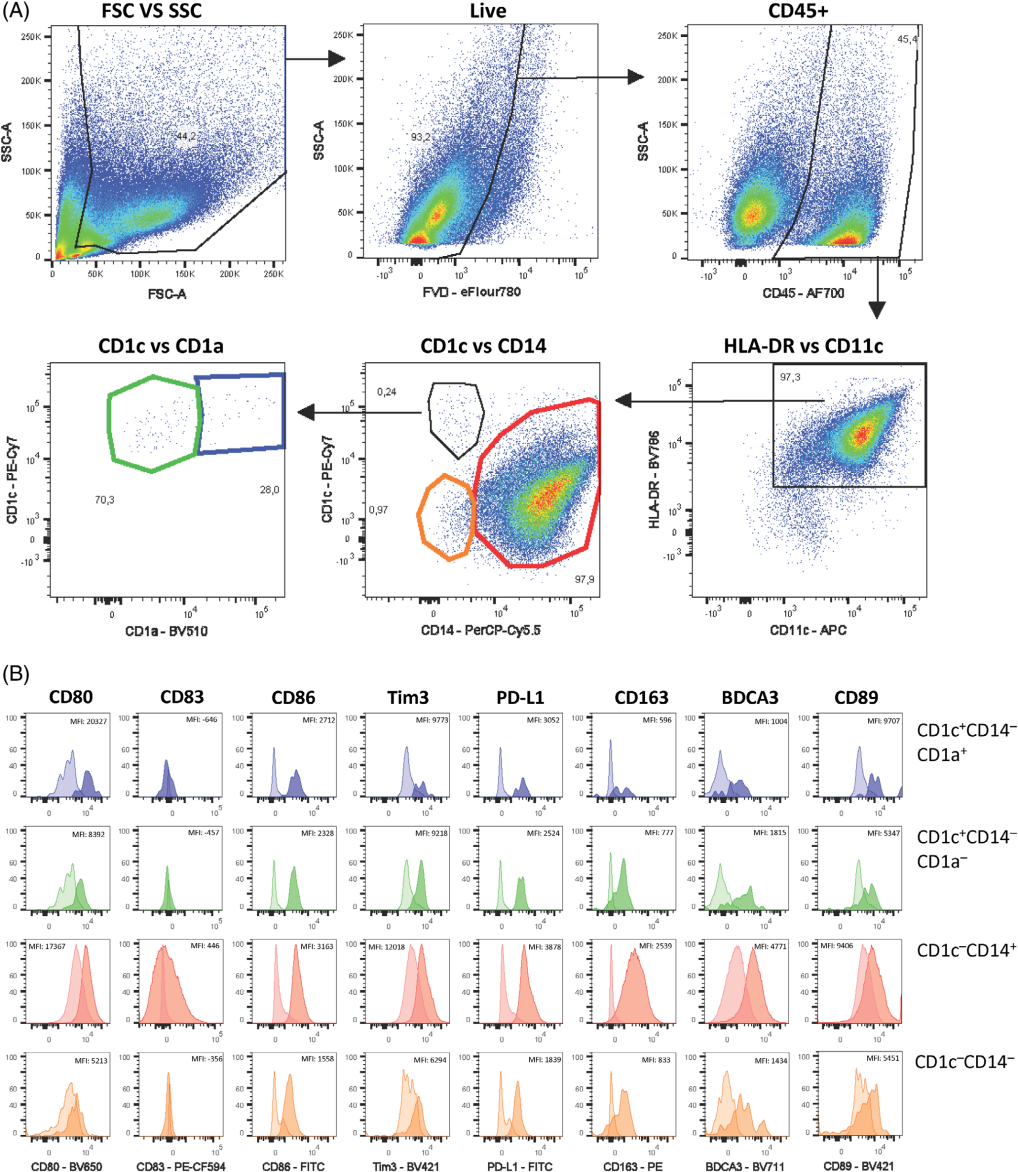
cDC gating strategy in the flow cytometric analysis of a single‐cell suspension derived from melanoma metastasis. (A) Gating strategy for the quantitation and phenotypic analysis of myeloid APC/cDC subsets among live CD45^+^ leukocytes. (B) Expression of subset‐defining and co‐stimulatory markers as well as PD‐L1 on the identified APC/cDC subsets. Histogram overlays with fluorescence‐minus‐one controls are shown.

Data acquisition was performed with a BD LSRFortessa X‐20 flow cytometer in a 3‐laser configuration (488 nm blue laser, 633 nm red laser, and 405 nm violet laser). Tables 30, 31, 32 show the used media, buffers, reagents, antibodies (including manufacturers, fluorochromes, clone names, and catalog numbers), necessary equipment, and workflow for antibody FACS staining. Of note, slightly different staining panels were used for the three samples, showcased in Fig. 9, 10, 11. We suggest building the panel by combining the markers shown in Table 32 based on the focus of specific research. In Table 33, a proposed staining panel is shown. This panel was used for the melanoma sample shown in Fig. 11. Acquired data were analyzed using FlowJo software (version 10.7).

**Table 33:** Proposed tumor DC staining panel.

	FITC	PE	PE‐CF594	PerCP‐Cy5.5	PE‐Cy7	APC	AF700	APC‐Cy7/H7^a^	BV421	V500/ BV510	BV650	BV711	BV786
1		FMM	FMM	CD14	CD1c	CD11c	CD45	FVD	FMM	FMM	FMM	FMM	HLA‐DR
2	PD‐L1	CD163	CD83	CD14	CD1c	CD11c	CD45	FVD	Tim3	CD1a	CD80	CD141	HLA‐DR
3	CD86	Tumor marker		CD14	CD1c	CD11c	CD45	FVD	CD89	CD1a		CD141	HLA‐DR

Abbreviations: FMM, fluorescence minus multiple; tumor marker, for example, EGFR EpCam, MCSP.

^a^ Or eFluor780.

Fig. 9, 10, 11 show the applied myeloid APC/cDC subset gating strategy for single‐cell suspensions derived from a primary colorectal tumor sample, a primary non‐small‐cell lung cancer (NSCLC) specimen, and a melanoma lymph node metastasis, respectively. After the gating of cells based on Forward and Side scatter properties (excluding lymphocytes), live cells were gated, and the live leukocytes were based on CD45 expression. Prior to CD45 gating, one could opt to also gate on singlets (carried out in Fig. 10A and 11A); we do, however, advise caution with singlet gating since DC often bind other cells (most notably T cells), this may lead to loss of DC from the analysis and one should check for this. Often lineage markers are included in DC analyses to exclude non‐DC in particular inclusion of CD19 would have relevance as B cells share various markers with cDC (CD1c amongst others). We have however found that excluding lymphocytes, based on scatter properties, and gating on relatively high CD11c levels (see Fig. 9, 10, 11) ensures the gating of myeloid cells. Next, HLA‐DR^+^ cells were selected to exclude possible HLA‐DR^−^ monocytic myeloid‐derived suppressor cells. This can be done against an empty channel, to evaluate autofluorescence (Fig. 9A) or by directly selecting CD11c^+^HLA‐DR^+^ cells (Fig. 10A and 11A). If not done simultaneously, after the HLA‐DR gate, CD11c^+^ cells should be selected. Subsequently, CD1c was plotted against CD14, identifying cDC‐like APCs (CD1c^+^) or more macrophage‐like APCs (CD1c^−^ and CD14^+^). In the CD1c^+^ cDC population, sometimes a CD1a^+^ subpopulation could be discerned (Fig. 10A and 11A). While CD1a is considered a backbone marker, its gating can be difficult without an FMO; this has been facilitated by the proposed panel in Table 33. Additional marker expression on these APC subsets, defined by their CD14, CD1c, and CD1a expression patterns, can inform their possible functionality and cellular origins (see Fig. 9, 10, 11). While CD11c^+^CD1c^+^CD14^−^ cDC might be considered cDC2, based on their CD1c expression, their expression of CD88 and CD89 belies this notion and indicates their monocytic origins (see Fig. 10B and 10C; [10]). Similarly, CD11c^+^CD14^−^CD1c^−^CD141^+^ APCs might be thought of as classic cDC1, but this is contrary to their expression of both CD88 and CD89 (Fig. 10B and 11B). CD141, known as a cDC1 marker, is upregulated by IL‐10, and like CD163 is widely expressed on the surface of tumor‐conditioned monocyte‐derived macrophages and cDC [91, 92]. To identify “true” cDC1, combined staining for XCR1 and/or CLEC9A is required, but in human tumors generally this subset is very rare. In fact, all CD11c^+^ APCs found in the here analyzed melanoma and colon tumors were positively identified as monocytic in origin, based on both CD88 and CD89 expression (Fig. 10B and 11B). This strongly suggests that all assessed subsets in actual fact represent contiguous differentiation stages of monocytes recruited in large numbers to the tumor. The observed differentiation states are determined by the prevailing cytokine conditions in the TME, with the predominant CD1c^−^CD14^+^ subset adopting a macrophage‐like phenotype, defined by expression of CD163, CD141, and PD‐L1, but, for example, in the case of the analyzed NSCLC, also of CD80, placing them anywhere on the spectrum between an M2‐like, tumor‐promoting state, or an M1‐like, putatively proinflammatory state (Fig. 10B). CD1c^+^ cDC‐like cells also express CD163 and PD‐L1, a clear sign of tumor‐imposed immune suppression. The least frequent CD1a^+^ population appears to be most advanced along the DC differentiation pathway, as evidenced by higher expression levels of CD80 and CD83 (Fig. 10B and 11B). This actually reflects in vitro MoDC differentiation, where CD1c expression precedes CD1a expression. Alternatively, CD1c^+^CD14^+^ cells may represent a separate monocyte or cDC2‐like subset, previously identified in both peripheral blood and metastatic lesions from melanoma patients, also expressing both CD163 and PD‐L1 and endowed with T‐cell suppressive capabilities [99]. In any case, we have shown that combined GM‐CSF/IL‐4 exposure and STAT3/p38 MAPK inhibition can efficiently convert CD14^+^ monocytic cells from metastatic melanoma‐derived single‐cell suspensions to CD1a^+^ cDC, fully consistent with the notion that tumors are predominantly infiltrated by monocytes, the differentiation of which may be therapeutically modulated to skew them toward a T‐cell stimulatory cDC state [95].

In conclusion, polychromatic flow cytometry can shed light on the myeloid APC/cDC populations present in single‐cell suspensions derived from clinical tumor samples. They provide a snapshot of cell populations with an exceptionally high phenotypic plasticity, constantly in flux, which poses serious challenges to the interpretation of the obtained data.

### 10.5 Pitfalls

Some cDC subsets in tumors are present at low frequencies; be sure to acquire as many events as possible, at least 2 × 10^5^. At low event counts it becomes hard to accurately identify and gate the smaller subsets. Also, rates of infiltrating immune cells can vary widely between samples (from virtually absent to over 80% of all cells).

There can be considerable interindividual fluctuations in the fluorescence intensity of the (backbone) markers on cDC subsets (even in lymph nodes from healthy donors) that may require compensation or gating adjustments. Also, autofluorescence may vary depending on the activation state of the cDC/myeloid APC but is generally quite high. Novel spectral flow cytometry platforms may help overcome these hurdles.

As cDC1 can express lower levels of CD11c than other cDC subsets [100], it is advisable to check for cDC1 in HLA‐DR^+^ cells expressing lower CD11c levels (if present). Caution is however warranted since immature cDC and macrophages (the latter expressing variable levels of CD11c and CD14) can acquire BDCA3/CD141 expression (e.g. induced by IL‐10); their expression of CD89 may further identify them as monocytic in origins rather than bona fide cDC1.

Important information that is lacking in flow cytometric data sets concerns the spatial context of APC/cDC in the TME. Are they found in tumor fields or stroma? What are their nearest neighbors, for example, effector T cells or regulatory T cells? This information may inform their putative functionality. High‐dimensional single‐cell platforms such as spatially resolved CyToF or transcriptomics can yield a wealth of data on the individual myeloid cells and relate this directly to their function and cross‐talk with other cells in the tumor tissue context.

### 10.6 Top tricks

#### 10.6.1 Tumor cell detection

The inclusion of EpCAM (for epithelial tumors) or MCSP (for melanoma) in the FACS panel also allows for the gating and analysis of tumor cells.

#### 10.6.2 Plasmacytoid DC

This protocol paper focused on cDC subsets, but obviously, pDC can also be analyzed by the use of, for example, CD303 and CD123.

### 10.7 Summary of the phenotype

The overall phenotype of immune cells covered by the markers included in the panel is detailed in Table 34.

**Table 34:** Summary of marker expression on analyzed cell populations.

	Monocyte/macrophages	Mo‐cDC	cDC1	cDC2	cDC3
CD11c	++	++	+	++	++
CD1c	−	+	–	+	+
CD1a	−	±	–	−	−
CD14	±	−^a^	–	−^b^	+
CD141	+	±	++	+	−
XCR1	−	−	+	−	−
CD88/89	+	+	−	−	−
HLA‐DR	+	++	+	+	+

^a^ Can become + under tumor‐mediated immune suppressive conditions

^b^ A subpopulation with CD14 expression is found in human tumors [99].

## 11 Preparation of single‐cell suspensions from human tumor‐draining lymph nodes

### 11.1 Introduction

Lymph nodes are secondary lymphoid organs, which are essential in orchestrating immune responses by being strategically placed throughout the body where they can bring together recirculating lymphocytes and antigen‐presenting cells, most notably DC, and where fluid containing soluble antigen is collected from afferent lymph vessels. DC are most powerfully equipped for the task of priming or tolerizing T cells, according to microenvironmental cues. Lymph nodes can contain various DC subsets, including migratory and LNR cDC, pDC. Which particular DC subsets are present in lymph nodes and what markers they present, can vary greatly depending on the tissue that the lymph node is draining. Through flow cytometric analysis of tumor‐free lymph nodes draining the primary tumor site in melanoma patients, we and others identified both CD1a^+^ dermal DC (DDC) and LC subsets, migrated from the skin, and CD1a^−^ LNR‐cDC subsets, derived from blood‐borne precursors [67, 101, 102]. Interestingly, although migratory DC displayed higher levels of activation and co‐stimulatory markers, LNR‐cDC proved to be the more potent primers of allogeneic T cells [67]. In vivo, studies recently provided evidence that interplay between migratory DC and LNR‐cDC subsets ultimately determines the antigen presentation to, and priming outcome of, effector cytotoxic T lymphocytes [69].

TDLN are in a category of their own. Not only can they contain metastasized tumor cells, which can disrupt the lymph node architecture and directly influence the local microenvironment [82, 103], but soluble factors secreted by (primary) tumors will also drain to the lymph node and can create an immune suppressed environment ready to receive metastasizing tumor cells in a process known as metastatic niche formation [104]. DC within TDLN often display a more suppressed phenotype with decreased expression of activation and co‐stimulatory molecules, and increased expression of immune checkpoint molecules [82, 103, 104]. Remarkably, in patients with early‐stage melanoma, we found decreased rates of migratory DC subsets in the first‐line draining TDLN, the so‐called SLN, to be associated with increased loco‐regional recurrence, whereas suppressed activation of LNR‐cDC subsets was associated with distant recurrence [82].

In order to thoroughly investigate the different aspects of DC biology in cancer and subsequent immunotherapy, we share this protocol to obtain single‐cell suspensions from TDLN material, without interference in any diagnostic procedures [105, 106] and propose a polychromatic flow cytometry panel, based on marker sets employed by us for the initial characterization of cDC subsets in TDLN as demonstrated in the section **12** **Flow cytometry analysis of conventional dendritic cell subsets from human tumor‐draining lymph nodes**.

### 11.2 Materials

#### 11.2.1 Reagents

A complete list of reagents is provided in Table 35.

**Table 35:** Reagents, antibodies, chemicals, and solutions.

Reagent	Manufacturer	Ordering number
RPMI 1640 medium	Lonza	BE12‐702F
Fetal Calf Serum	Hyclone	SV30160.03
Penicillin‐streptomycin‐glutamine	Gibco	10378016
Collagenase A	Roche	10103586001
Deoxyribonuclease I (DNAse I)	Roche	10104159001

#### 11.2.2 Equipment

Necessary equipment are listed in Table 36.

**Table 36:** Necessary equipment.

Equipment	Company	Purpose
1.5 or 2 mL reaction tube	Eppendorf	preparation of staining mix, Live/Dead dye solution
Surgical blade (size no.22)	Swann Morton Ltd.	To scrape TDLN cut surface
Dissociation flask with magnetic stir bar	Wheaton, USA	To contain TDLN cells during enzymatic digestion
Submersible magnetic stirrer	Thermo Scientific	To stir TDLN cells during enzymatic dissociation
100 µm cell strainer	FALCON	Filtering of cell suspension
50 mL conical tube	FALCON	Store and wash cells after isolation
Pipettes	Eppendorf	Washing and cell/mAb dispensing

### 11.3 Step‐by‐step sample preparation

#### 11.3.1 Preparation of media and buffer

##### 11.3.1.1 Complete medium

RPMI 1640 supplemented with 10% FCS, 100 µg/mL streptomycin sulfate, 100 I.E./mL sodium penicillin, 2 mM l‐glutamine (P/S/G).

##### 11.3.1.2 Dissociation medium

RPMI 1640 supplemented with P/S/G, 0.1% DNase I, 0.14% collagenase A, and 5% FCS.

#### 11.3.2 TDLN processing to single‐cell suspension

TDLN were sampled under written informed consent, according to a protocol approved by the Amsterdam UMC institutional review board. Sampling was carried out under the supervision of a pathologist, and only when the lymph node diameter exceeded 0.5 cm.

1. Collect TDLN(s) immediately after surgical removal in 10 mL complete medium in a 50 mL polypropylene tube. The TDLN should be at least 0.5 cm in diameter.
2. If further isolation and culture or functional testing of TDLN is planned, preferably process TDLN(s) in a sterile environment like a laminar flow hood; or keep processed TDLN as sterile as possible.
3. Prepare TDLN and cut pieces of fat away.
4. Cut TDLN(s) into two halves crosswise with a sterile scalpel (no. 22) on a sterile Petri dish on ice.
5. Optional (for later immunohistochemical analysis): make imprints by dabbing the cut surface(s) of the TDLN(s) (smallest half) on clean glass slides (4 per slide; 20 slides per cut surface). Let these imprints dry overnight at RT before 10 min fixation in acetone (after fixation, store slides at –20°C or −80°C).
6. Scrape the cut surfaces of the TDLN with the surgical blade at an angle of 45°; in case of scraping one cut surface, select the largest one. Using this method allows the use of the remaining lymph node for diagnostic purposes.
7. Detach cells from the surgical blade by vigorously stirring the scalpel in 50 mL tube containing 10 mL dissociation medium.
8. Repeat scraping several times (to a total of 10 per cut surface). Stir the surgical blade in the same tube after every scrape to collect cells.
9. Transfer the 10 mL dissociation medium with the TDLN cells to a sterile flask with a magnetic stir bar, rinse the 50 mL tube twice more with 10 mL dissociation medium, and add to the same flask (to a total volume of 30 mL).
10. Place a magnet stirrer in a water bath at 37°C.
11. Put the flask on the stirrer and switch on the stirrer; leave the suspension to stir at a gentle rotation for 30 min.
12. Switch off the stirrer and run the cell suspension from the flask through a 100 µm‐pore cell strainer; rinse the cell strainer twice with 10 mL complete medium and collect the cell suspension in a new sterile 50 mL conical PP tube.
13. Centrifuge for 5 min at 530 × *g* and 4°C.
14. Resuspend cell pellet in 10 mL complete medium, or more depending on the size of the pellet.
15. Count cells and either cryostore or proceed with flow cytometry analysis as detailed in the section **12** **Flow cytometry analysis of conventional dendritic cell subsets from human tumor‐draining lymph nodes**.

### 11.4 Data analysis

Examples of flow cytometric data analysis of cDC subsets from tumor‐draining lymph nodes using the described single cell suspensions are shown in detail in the section **12** **Flow cytometry analysis of conventional dendritic cell subsets from human tumor‐draining lymph nodes**.

### 11.5 Pitfalls

Cell yields from the described harvesting method, that is, through the scraping of TDLN cutting surfaces, can vary dramatically and range between 1 × 10^5^ and 50 × 10^6^, depending on experience of the investigator and consistency of the TDLN, as well as prior applied treatment regimens (e.g., immuno‐, chemo‐, or radiotherapy).

If yields allow, TDLN‐derived single‐cell suspensions can be viably cryopreserved but note that in particular the CD14^+^ cDC subset may go down in frequency after cryostorage and thawing [107].

### 11.6 Top tricks

#### 11.6.1 Cell viability

The interval from collection to digestion significantly impacts cell viability. Minimizing this timeframe is crucial. Moreover, the tissue should be maintained on ice consistently until digestion.

#### 11.6.2 Prevention of loss of membrane markers during enzymatic digestion

Membrane proteins may be cleaved during DNAse/Collagenase digestion. We have found that the addition of 5% FCS to the dissociation medium could largely prevent this [77].

#### 11.6.3 Sampling TDLN that are not essential for pathological staging and/or clinical decision making

When TDLN, unlike SLN, are not essential for subsequent clinical staging and/or clinical decision‐making, a chunk (e.g. half) of the TDLN may be available for analysis. In that case, first cut the TDLN in 1–2 mm pieces using a (sterile) surgical blade and forceps, before enzymatic digestion and further processing as listed above in the “Step by step sample preparation” paragraph.

#### 11.6.4 Imprints for immunohistochemistry

Rather than scraping both cut surfaces for live cell harvesting, one may consider making imprints from one of the cut surfaces for later immunohistochemistry analysis. Imprints are made by dabbing the cut surface of the TDLN on clean glass slides (4 per slide; 20 slides per cut surface). These imprints are dried overnight at RT before 10 min fixation in acetone (after fixation, slides can be stored at –20 or −80°C). Depending on the quality (i.e. cell density) of the imprints, single cells can be stained and analyzed in a spatial context in parallel to flow cytometry‐based analysis [67].

#### 11.6.5 Culture of TDLN single‐cell suspensions

The obtained suspensions can also be used for in vitro culture and immune modulation. We have successfully studied cDC and pDC activation through exposure to TLR ligands in cultures that were maintained for up to five days. Although DC rates dropped in the cultures after two days, their relative frequency and activation state could still readily and reliably be assessed by FACS analysis at day 5 (and compared with a medium control condition), which also allowed for the assessment of indirect activation of human LNR‐cDC subsets upon TLR9‐ligand exposure [108].

#### 11.6.6 Transcriptional and functional assays

The cells obtained with this protocol can be subsequently used not only for flow cytometry as described in the section **12** **Flow cytometry analysis of conventional dendritic cell subsets from human tumor‐draining lymph nodes** of this section but also for transcriptomics as well as functional assays.

## 12 Flow cytometry analysis of conventional dendritic cell subsets from human tumor‐draining lymph nodes

### 12.1 Introduction

The role of TDLN and DC as key players in tumor immunology and the immunotherapy of cancer has recently been (re)discovered. We and others have shown the essential role of TDLN in immunotherapy efficacy, in preclinical models and in the clinical trial setting [102, 109–114]. When the TDLN was surgically resected before tumor‐bearing mice were treated with either PD‐1 or PD‐L1 blocking antibody, the therapeutic efficacy was profoundly decreased [112, 115]. And when melanoma patients were injected with the TLR9‐ligand CPG7909 into the scar where shortly before the primary tumor had been resected while the TDLN were left in place, not only was there clear immune activation within the first‐line TDLN, the so‐called SLN, but importantly, this also led to a significantly increased systemic anti‐tumor T‐cell response, and strongly decreased recurrence of disease compared with placebo‐treated patients [102, 111]. It has become clear that pDC and LNR‐cDC, respectively directly and indirectly activated by the injected TLR9‐ligand, play a pivotal role in this process.

In order to thoroughly investigate the different aspects of DC biology in cancer and subsequent immunotherapy, we share this protocol to obtain single‐cell suspensions from TDLN material, without interference in any diagnostic procedures [105, 106], and propose a polychromatic flow cytometry panel, based on marker sets employed by us for the initial characterization of cDC subsets in TDLN.

### 12.2 Materials

#### 12.2.1 Reagents

A complete list of reagents is provided in Table 37 and of used antibodies in Table 38.

**Table 37:** Reagents, chemicals, and solutions.

Reagent	Manufacturer	Ordering number
BSA (albumin bovine fraction V)	Sigma‐Aldrich	B6917
Brilliant staining buffer	BD	563794

**Table 38:** Reagents and antibodies used for flow cytometric analysis

Specificity	Fluorochrome	Clone	Manufacturer	Catalog #	Dilution
CD1a	PE	5c3 HI149	BD	555807	1:50
CD14	PerCP‐Cy5.5	MΦP9	BD	562692	1:20
CD1c	PE‐Cy7	L161	Sony	2257580	1:100
CD141	FITC	AD5‐14H12	Miltenyi Biotec	130‐090‐513	1:50
CD19	PE‐CF594	HIB19	BD	562294	1:100
CD80	FITC	L307.4	BD	557226	1:50
CD83	FITC	HB15a	Beckman Coulter	PN IM2410U	1:50
CD11c	APC	S‐HCL‐3	BD	333144	1:100
CD45	AF700	HI30	BioLegend	304024	1:20
FVD (live/dead)	eFluor780		eBioscience	65‐0865‐14	1:1000
EpCAM	BV421	EBA‐1	BD	563180	1:100
CD163	BV421	GHI/61	BD	562643	1:75
CD40	BV421	5C3	BD	563396	1:25
TIM3	BV421	F38‐2E2	BioLegend	345008	1:20
PD‐L2	BV711	MIH18	BD	564258	1:25
PD‐L1	BV786	MIH1	BD	563739	1:25
HLA‐DR	BV786	L243	BioLegend	307642	1:200

#### 12.2.2 Equipment

Necessary equipment are listed in Table 39.

**Table 39:** Necessary equipment.

Equipment	Company	Purpose
1.5 or 2 mL reaction tube	Eppendorf	Preparation of staining mix, Live/Dead dye solution
5 mL FACS tubes	FALCON	For cell staining and analysis
50 mL conical tube	FALCON	Store and wash cells after isolation
Pipettes	Eppendorf	Washing and cell/mAb dispensing
Centrifuge	Hettich Zentrifugen	Centrifugation of cells
LSRFortessa X‐20	BD	Flow cytometry analysis

### 12.3 Step‐by‐step sample preparation

#### 12.3.1 Preparation of media and buffer

FACS staining buffer: PBS supplemented with 0.1% BSA and 0.02% NaN_3_.

Live/Dead dye solution: dilute fixable viability dye eFluor 780 1:1000 in PBS.

#### 12.3.2 Flow cytometry analysis

In the section **11** **Preparation of single‐cell suspensions from human tumor‐draining lymph nodes**, we provide a detailed protocol on how to isolate cells from human tumor‐draining lymph nodes for analysis by flow cytometry.

1. Start staining for flow cytometry analysis with 2–5 × 10^5^ cells per tube and add 2 mL FACS staining buffer.
2. Centrifuge at 530 × *g*, 5 min, 4°C.
3. Discard sup and resuspend pellet in 2 mL FACS staining buffer per FACS‐tube
4. Centrifuge at 530 × *g*, 5 min, 4°C.
5. Resuspend cell pellets in Live/Dead dye solution, incubate in the dark at RT for 5 min, and add 2 mL FACS staining buffer to wash.
6. Centrifuge at 530 × *g*, 5 min, 4°C.
7. Discard supernatant, resuspend pellet in 100 µL of antibody staining mix containing the correct final dilution of antibodies in FACS staining buffer 1:1 diluted with Brilliant staining buffer
8. Incubate for 30 min at 4°C in the dark (see Table 40).
9. Wash cells using FACS staining buffer and centrifuge at 1500 rpm, 5 min, 4°C.
10. Discard sup and resuspend cells in 200–300 µL of FACS staining buffer and keep in the dark at 4°C until data acquisition on a BD LSRFortessa X‐20.

**Table 40:** Staining workflow for human TDLN cDC panel.

	Marker	Fluorochrome	Dilution	Diluent	Incubation (min/temp)
**1**	FVD	eFluor780	1:10,000	PBS	5’/4°C
					
**2**	Wash (staining buffer)				
**3**	Backbone markers:				
	CD11c	APC	1:100	FACS staining buffer:Brilliant stain buffer (1:1)	30’/4°C
	CD14	PerCP‐Cy5.5	1:20		
	CD1a	PE	1:100		
	CD1c	PE‐Cy7	1:100		
	CD45	AF700	1:20		
	(CD19	PE‐CF594	1:100)		
	Additional markers:				
	CD80	FITC	1:50		
	CD83	FITC	1:50		
	EpCAM	BV421	1:100		
	CD163	BV421	1:75		
	CD141	FITC	1:50		
	CD40	BV421	1:25		
	TIM3	BV421	1:20		
	PD‐L2	BV711	1:25		
	PD‐L1	BV786	1:25		
	HLA‐DR	BV786	1:200		
**4**	Wash (staining buffer)				
**5**	Resuspend cells in 200–300 µL of staining buffer and keep in the dark at 4°C until acquisition				

### 12.4 Data analysis

Data acquisition was performed with a BD LSRFortessa X‐20 flow cytometer in a 3‐laser configuration (488 nm blue laser, 633 nm red laser, and 405 nm violet laser). Tables 37, 38, 39, 40 show the used media, buffers, reagents, antibodies (including manufacturers, fluorochromes, clone names, and catalog numbers), necessary equipment, and workflow for antibody FACS staining. Acquired data were analyzed using FlowJo software (version 10.7).

**Figure 12:**
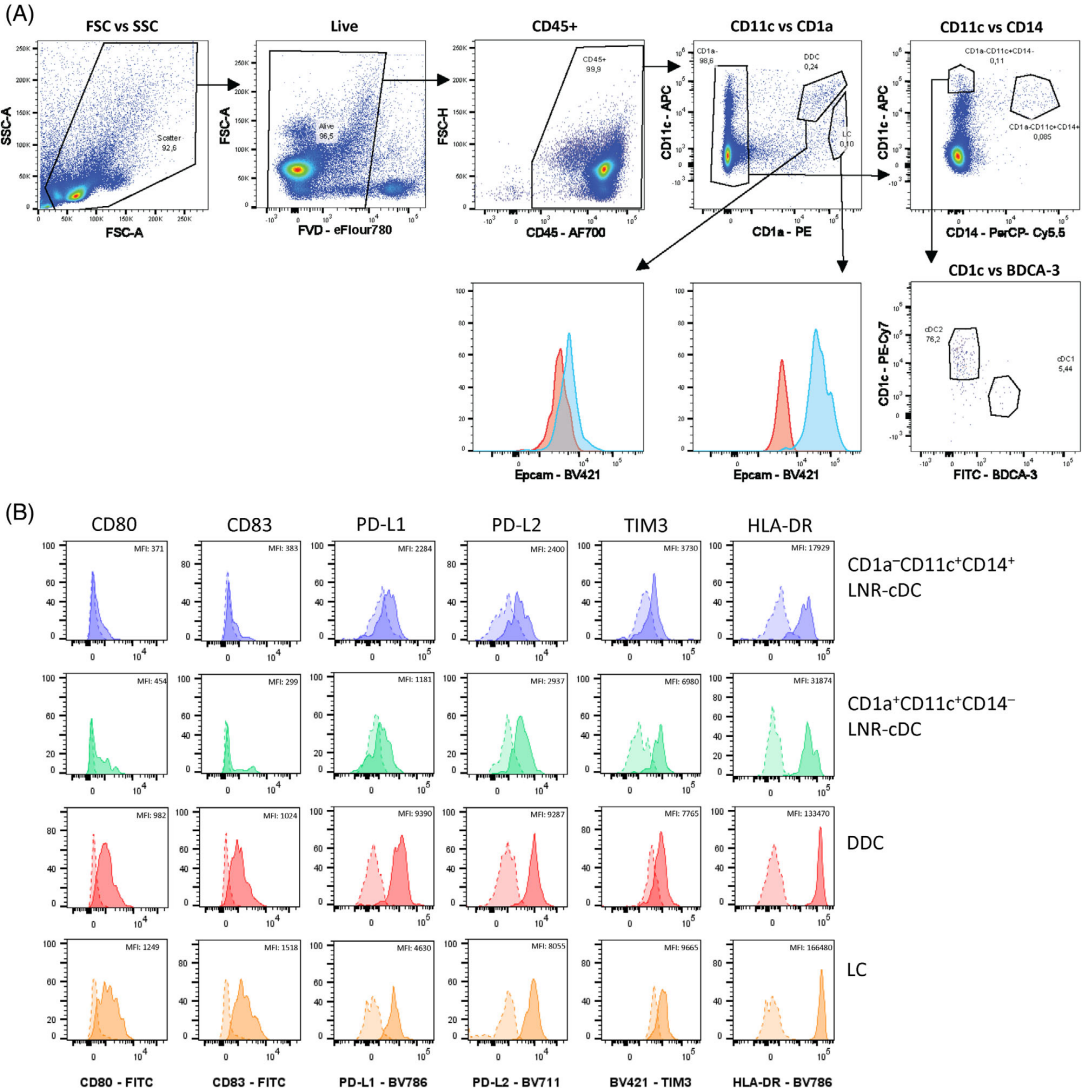
cDC subset gating strategy in Melanoma TDLN. (A) Gating strategy for the quantitation and phenotypic analysis of two migratory and two LNR‐cDC subsets among live CD45^+^ leukocytes. (B) Expression of activation and co‐stimulatory markers as well as immune checkpoints on the identified cDC subsets. Histogram overlays with fluorescence‐minus‐one controls are shown.

**Figure 13:**
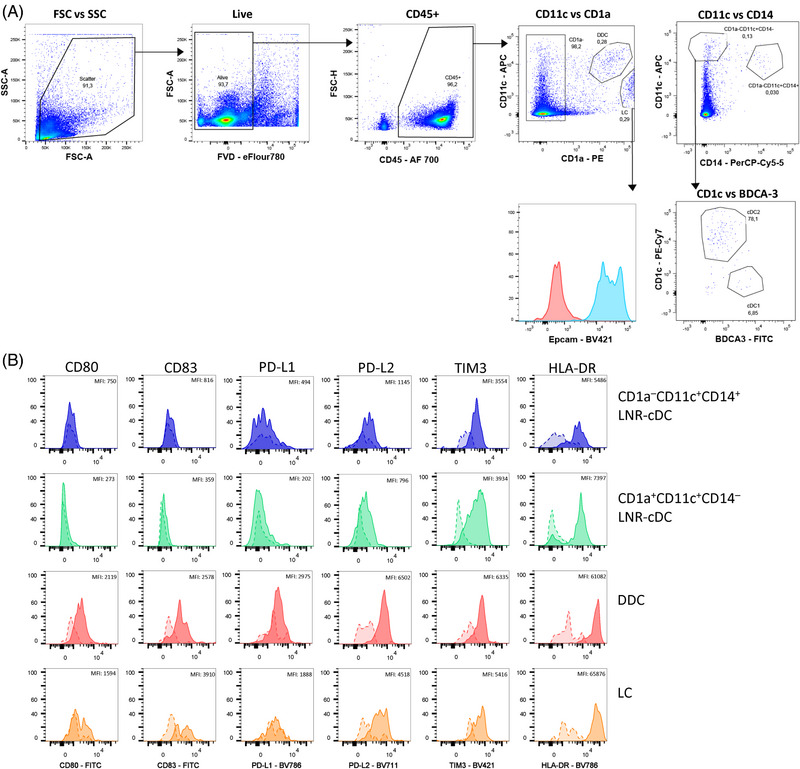
cDC subset gating strategy in mammary TDLN. (A) Gating strategy for the quantitation and phenotypic analysis of two migratory and two LNR‐cDC subsets among live CD45^+^ leukocytes. (B) Expression of activation and co‐stimulatory markers as well as immune checkpoints on the identified cDC subsets. Histogram overlays with fluorescence‐minus‐one controls are shown.

**Figure 14:**
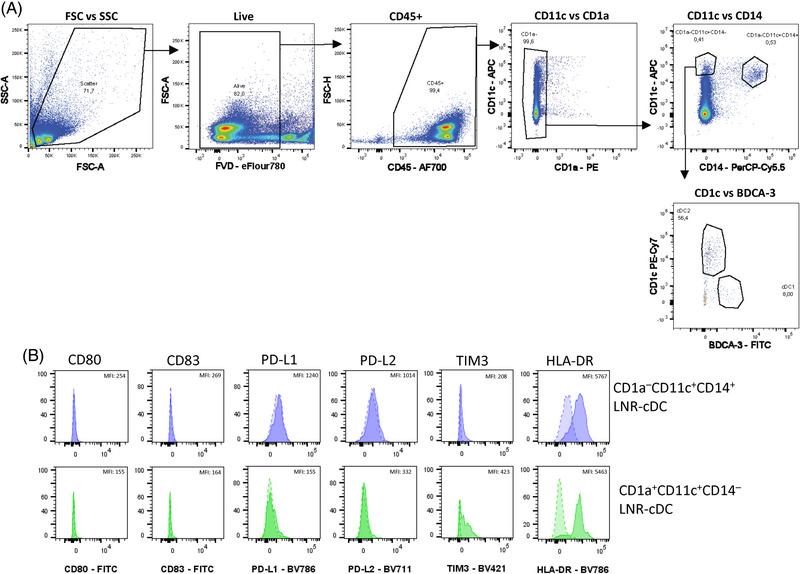
cDC subset gating strategy in non‐small‐cell lung cancer TDLN. (A) Gating strategy for the quantitation and phenotypic analysis of cDC subsets among live CD45^+^ leukocytes. Note the absence of detectable CD1a^+^ migratory subsets. (B) Expression of activation and co‐stimulatory markers as well as immune checkpoints on the identified cDC subsets. Shown are histogram overlays with fluorescence‐minus‐one controls.

Fig. 12, 13, 14 show the employed cDC subset gating strategy for TDLN derived from a melanoma, a mammary carcinoma, and a NSCLC, respectively. Of note, slightly different staining panels were used for the three samples showcased in Fig. 12, 13, 14. We suggest building the panel by combining the markers shown in Table 40 based on the focus of specific research. After gating of cells based on forward and side scatter properties, live cells were gated, and the live leukocytes based on CD45 expression. Prior to CD45 gating, one could opt to also gate on singlets; however, we have found that since DC often bind other cells (most notably T cells), this may lead to loss of DC from the analysis. To avoid cell adhesion or clumping, EDTA or DNAse‐I could be added to the FACS staining buffer. After CD45 gating, CD11c was plotted against CD1a. In skin‐draining TDLN, migratory cDC subsets could now be gated based on CD1a expression, with DDC expressing high levels of CD11c, and LC expressing low levels of CD11c and high levels of CD1a [67, 104]. NB: LC identity could be further confirmed by EpCAM expression (Fig. 12A and 13A), which was low or absent on DDC (Fig. 13A). Note that in visceral, non‐skin‐draining TDLN, like those derived from NSCLC, often no CD1a^+^ migratory cDC can be discerned (see Fig. 14A; [105]). Next, CD1a^−^ cells were gated and CD11c was plotted against CD14. Two LNR‐cDC subsets could now be distinguished: CD11c^hi^CD14^−^ or CD11c^hi^CD14^+^ [67]. The bona fide DC identity of the former LNR‐cDC subset may be confirmed by the inclusion of lineage markers (like CD19) in the panel, but experience has taught us that gating on CD11c^hi^ cells guarantees DC gating [67]. CD11c^hi^CD14^−^ LNR‐cDC could be further defined as cDC2 or cDC1, based on CD1c versus CD141 plotting, with CD1c^+^CD141^lo^ cells representing cDC2, and CD1c^−^CD141^+^ cells representing cDC1. Usually, the latter constitute less than 5–10% of the CD11c^hi^CD14^−^ LNR‐cDC subset, with their numbers too low for reliable analysis of expression of further (activation) markers.

Further analysis of markers expressed on the four cDC subsets (like co‐stimulatory molecules or immune checkpoints), revealed differences between migratory versus resident subsets, and differences between tumor types (see Fig. 12, 13, 14). Most notably, LNR‐cDC seemed to be in a less activated state than migratory cDC, and cDC in general seemed to be more activated in the melanoma‐derived TDLN than in the mammary carcinoma, or NSCLC‐derived TDLNs. The latter may be due to the fact that the melanoma TDLN was an SLN, removed at least four weeks after the primary melanoma was removed, whereas the other two TDLNs were removed simultaneously with their primary tumors. As a result, tumor‐induced immune suppression might have been waning in the melanoma TDLN, but still very much in force in the mammary carcinoma‐ and NSCLC‐derived TDLNs. Interestingly, TIM3 seemed to be particularly highly expressed on all cDC subsets derived from the mamma TDLN, which is in keeping with previous reports that TIM3 was expressed on cDC in breast tumors and thus interfered with their cGAS/STING mediated activation and their co‐localization with CD8^+^ T cells [116, 117, 118].

### 12.5 Pitfalls

cDC subsets in TDLN are present at low frequencies (ranging from 0.1 to 1.0% of total leukocytes): be sure to acquire as many events as possible, at least 1–2 × 10^5^. At low event counts it becomes hard to accurately gate them.

There can be some inter‐individual fluctuations in the fluorescence intensity of the (backbone) markers on cDC subsets (even in lymph nodes from healthy donors) that may require compensation or gating adjustments. Also, autofluorescence may vary depending on the activation state of the cDC.

As cDC1 can express slightly lower levels of CD11c than the other LNR‐cDC subsets [100], it is advisable to check for cDC1 at lower CD11c levels. Caution is however warranted since immature cDC and macrophages (the latter expressing variable levels of CD11c and CD14) can acquire BDCA3/CD141 expression under tumor‐associated conditions (e.g. induced by IL‐10).

### 12.6 Top tricks

#### 12.6.1 Tumor cell detection

Inclusion of EpCAM in the cDC panel also allows for the gating (as CD45^−^ EpCAM^+^) and analysis of tumor cells in the TDLN from epithelial tumors, for example, for the expression of HLA‐DR or PD‐L1.

#### 12.6.2 Plasmacytoid DC

This protocol paper focused on cDC subsets, but obviously, pDC can also be analyzed by the use of, for example, CD303 and CD123. We found that pDC could easily be detected as CD11c^−^ and CD123^hi^ or CD303^+^.

### 12.7 Summary of the phenotype

The overall phenotype of immune cells covered by the markers included in the panel is detailed in Table 41.

**Table 41:** Summary of marker expression on TDLN cDC populations.

	Langerhans cells	Dermal DC	CD14^−^ LNR‐cDC	CD14^+^ LNR‐cDC
CD11c	+	++	++^b^	++
CD1a	++	+	−	−
EpCAM	+	−	−	−
CD14	−^a^	−^a^	−	+
HLA‐DR	+	+	+	+

Abbreviation: LNR, lymph node‐resident.

^a^ Can become + under tumor‐mediated immune suppressive conditions.

^b^ Consists mostly of CD1c^+^CD141^+^ cDC2 and for 5–20% of CD141^hi^ cDC1.

## 14 Conflict of interest

The authors declare no commercial or financial conflict of interest.

## 15 Author contributions

Sections **1** and **2** **Preparation of human single‐cell suspensions of the lung** and **Flow cytometric analysis of the human lung DC compartment**

16

Regine J. Dress, Charles‐Antoine Dutertre and Florent Ginhoux, lead author Florent Ginhoux

17

Section **3** **Preparation of single‐cell suspensions from human skin**

18

Claudia Zelle‐Rieser, Christoph H. Tripp, Helen Strandt and Patrizia Stoitzner, lead author Patrizia Stoitzner

19

Section **4** **Flow cytometry analysis of DC subsets in human skin**

20

Helen Strandt, Florian Hornsteiner, Martina M Sykora, Christoph H Tripp, Sieghart Sopper and Patrizia Stoitzner, lead author Patrizia Stoitzner

21

Section **5** **Preparation of single‐cell suspensions of human gingiva**

22

Paz Kles, Or Saar, Yael Horev, Oded Heyman, Gabriel Mizraji and Asaf Wilensky, lead author Asaf Wilensky

23

Section **6** **Flow cytometry analysis of human dendritic cell subsets in the gingiva**

24

Or Saar, Paz Kles, Ruth Lubin, Yael Horev, Oded Heyman, Gabriel Mizraji, Simon Yona and Asaf Wilensky, lead author Asaf Wilensky

25

Sections **7** and **8** **Preparation of single‐cell suspensions from immune compartments of the human intestine** and **Flow cytometry analysis of cDC from immune compartments of the human intestine**

26

Thomas M. Fenton and William W Agace, lead author William W Agace

27

Sections **9** and **10** **Preparation of single‐cell suspensions from human tumors** and **Flow cytometry analysis of conventional dendritic cells in human tumors**

28

Anastasia Prokopi, Elisa C. Toffoli, Vinitha Kandiah, Marieke F. Fransen, Rieneke van de Ven, and Tanja D. de Gruijl, lead author Tanja D. de Gruijl

29

Sections **11** and **12** **Preparation of single‐cell suspensions from human tumor‐draining lymph nodes** and **Flow cytometry analysis of conventional dendritic cell subsets from human tumor‐draining lymph nodes**

30

Marieke F. Fransen, Vinitha Kandiah, Kim van Pul, Joyce Bakker, Rieneke van de Ven, and Tanja D. de Gruijl, lead author Tanja D. de Gruijl

31

### 31.2 Peer review

The peer review history for this article is available at https://publons.com/publon/10.1002/eji.202250325.

**Abbreviations**
AF: autofluorescent
BALF: bronchioalveolar lavage fluid
cDC: conventional DC
DDC: dermal DC
DNAse I: deoxyribonuclease I
GALT: gut‐associated lymphoid tissues
ILFs: isolated lymphoid follicles
LC: Langerhans cells
LNR: lymph node‐resident
LP: lamina propria
NSCLC: non‐small‐cell lung cancer
pDC: plasmacytoid DC
PP: Peyer's patches
SI: small intestine
SLN: sentinel lymph node
TDLN: tumor‐draining lymph nodes
TDLN: Tumor‐draining lymph nodes
TME: tumor microenvironment

## Data Availability

The data that support the findings of this study are available from the lead author of the respective section upon reasonable request.

## References

[1] Baharom, F. , Rankin, G. , Blomberg, A. and Smed‐Sörensen, A. , Human lung mononuclear phagocytes in health and disease. Front. Immunol. 2017. 8: 499. 28507549 10.3389/fimmu.2017.00499 PMC5410584

[2] Guilliams, M. , Dutertre, C. A. , Scott, C. L. , McGovern, N. , Sichien, D. , Chakarov, S. , Van Gassen, S. et al., Unsupervised high‐dimensional analysis aligns dendritic cells across tissues and species. Immunity 2016. 45: 669–684. 27637149 10.1016/j.immuni.2016.08.015 PMC5040826

[3] Condon, T. V. , Sawyer, R. T. , Fenton, M. J. and Riches, D. W. H. , Lung dendritic cells at the innate‐adaptive immune interface. J. Leukoc. Biol. 2011. 90: 883–895. 21807741 10.1189/jlb.0311134 PMC3206474

[4] Szabo, P. A. , Dogra, P. , Gray, J. I. , Wells, S. B. , Connors, T. J. , Weisberg, S. P. , Krupska, I. et al., Longitudinal profiling of respiratory and systemic immune responses reveals myeloid cell‐driven lung inflammation in severe COVID‐19. Immunity 2021. 54: 797–814.e6. 33765436 10.1016/j.immuni.2021.03.005 PMC7951561

[5] Schulte‐Schrepping, J. , Reusch, N. , Paclik, D. , Baßler, K. , Schlickeiser, S. , Zhang, B. , Krämer, B. et al., Severe COVID‐19 is marked by a dysregulated myeloid cell compartment. Cell 2020. 182: 1419–1440.e23. 32810438 10.1016/j.cell.2020.08.001 PMC7405822

[6] Dress, R. J. and Ginhoux, F. , Monocytes and macrophages in severe COVID‐19 – friend, foe or both? Immunol. Cell Biol. 2021. 99: 561–564. 34053124 10.1111/imcb.12464 PMC8242688

[7] Steinman, R. M. and Cohn, Z. A. , Identification of a novel cell type in peripheral lymphoid organs of micE. J. Exp. Med. 1973. 137: 1142–1162. 4573839 10.1084/jem.137.5.1142 PMC2139237

[8] Dress, R. J. , Wong, A. Y. and Ginhoux, F. , Homeostatic control of dendritic cell numbers and differentiation. Immunol. Cell Biol. 2018. 96: 463–476. 29473216 10.1111/imcb.12028

[9] See, P. , Dutertre, C.‐A. , Chen, J. , Günther, P. , McGovern, N. , Irac, S. E. , Gunawan, M. et al., Mapping the human DC lineage through the integration of high‐dimensional techniques. Science (80‐.) 2017. 356: 3009. 10.1126/science.aag3009 PMC7611082 28473638

[10] Dutertre, C. A. , Becht, E. , Irac, S. E. , Khalilnezhad, A. , Narang, V. , Khalilnezhad, S. , Ng, P. Y. et al., Single‐Cell Analysis of Human Mononuclear Phagocytes Reveals Subset‐Defining Markers and Identifies Circulating Inflammatory Dendritic Cells. Immunity 2019. 51: 573–589.e8. 31474513 10.1016/j.immuni.2019.08.008

[11] Merad, M. , Sathe, P. , Helft, J. , Miller, J. and Mortha, A. , The dendritic cell lineage: ontogeny and function of dendritic cells and their subsets in the steady state and the inflamed setting. Annu. Rev. Immunol. 2013. 31: 563–604. 23516985 10.1146/annurev-immunol-020711-074950 PMC3853342

[12] Dress, R. J. , Dutertre, C. A. , Giladi, A. , Schlitzer, A. , Low, I. , Shadan, N. B. , Tay, A. et al., Plasmacytoid dendritic cells develop from Ly6D+ lymphoid progenitors distinct from the myeloid lineage. Nat. Immunol. 2019. 20: 852–864. 31213723 10.1038/s41590-019-0420-3

[13] Bauer, J. , Dress, R. J. , Schulze, A. , Dresing, P. , Ali, S. , Deenen, R. , Alferink, J. et al., Cutting Edge: IFN‐β expression in the spleen is restricted to a subpopulation of plasmacytoid dendritic cells exhibiting a specific immune modulatory transcriptome signature. J. Immunol. 2016. 196: 4447–4451. 27183572 10.4049/jimmunol.1500383

[14] Steinman, R. M. , Decisions about dendritic cells: past, present, and future. Annu. Rev. Immunol. 2012. 30: 1–22. 22136168 10.1146/annurev-immunol-100311-102839

[15] Guilliams, M. , Ginhoux, F. , Jakubzick, C. , Naik, S. H. , Onai, N. , Schraml, B. U. , Segura, E. et al., Dendritic cells, monocytes and macrophages: a unified nomenclature based on ontogeny. Nat. Rev. Immunol. 2014. 14: 571–578. 25033907 10.1038/nri3712 PMC4638219

[16] Collin, M. , McGovern, N. and Haniffa, M. , Human dendritic cell subsets. Immunology 2013. 140: 22–30. 23621371 10.1111/imm.12117 PMC3809702

[17] O'Keeffe, M. , Mok, W. H. and Radford, K. J. , Human dendritic cell subsets and function in health and disease. Cell. Mol. Life Sci. 2015. 72: 4309–4325. 26243730 10.1007/s00018-015-2005-0 PMC11113503

[18] Villar, J. and Segura, E. , Decoding the heterogeneity of human dendritic cell subsets. Trends Immunol. 2020. 41: 1062–1071. 33250080 10.1016/j.it.2020.10.002

[19] Haniffa, M. , Gunawan, M. and Jardine, L. , Human skin dendritic cells in health and disease. J. Dermatol. Sci. 2015. 77: 85–92. 25301671 10.1016/j.jdermsci.2014.08.012 PMC4728191

[20] Klechevsky, E. , Human dendritic cells — stars in the skin. Eur. J. Immunol. 2013. 43: 3147–3155. 24222336 10.1002/eji.201343790

[21] Clausen, B. E. and Stoitzner, P. , Functional specialization of skin dendritic cell subsets in regulating T cell responses. Front. Immunol. 2015. 6: 534. 26557117 10.3389/fimmu.2015.00534 PMC4617171

[22] Romani, N. , Clausen, B. E. and Stoitzner, P. , Langerhans cells and more: langerin‐expressing dendritic cell subsets in the skin. Immunol. Rev. 2010. 234: 120–141. 20193016 10.1111/j.0105-2896.2009.00886.x PMC2907488

[23] McGovern, N. , Schlitzer, A. , Gunawan, M. , Jardine, L. , Shin, A. , Poyner, E. , Green, K. et al., Human dermal CD14 + cells are a transient population of monocyte‐derived macrophages. Immunity 2014. 41: 465–477. 25200712 10.1016/j.immuni.2014.08.006 PMC4175180

[24] Chu, C.‐C. , Ali, N. , Karagiannis, P. , Di Meglio, P. , Skowera, A. , Napolitano, L. , Barinaga, G. et al., Resident CD141 (BDCA3)+ dendritic cells in human skin produce IL‐10 and induce regulatory T cells that suppress skin inflammation. J. Exp. Med. 2012. 209: 935–945. 22547651 10.1084/jem.20112583 PMC3348099

[25] Haniffa, M. , Shin, A. , Bigley, V. , McGovern, N. , Teo, P. , See, P. , Wasan, P. S. et al., Human tissues contain CD141(hi) cross‐presenting dendritic cells with functional homology to mouse CD103(+) nonlymphoid dendritic cells. Immunity 2012. 37: 60–73. 22795876 10.1016/j.immuni.2012.04.012 PMC3476529

[26] Kashem, S. W. , Haniffa, M. and Kaplan, D. H. , Antigen‐presenting cells in the skin. Annu. Rev. Immunol. 2017. 35: 469–499. 28226228 10.1146/annurev-immunol-051116-052215

[27] Romano, E. , Cotari, J. W. , Barreira da Silva, R. , Betts, B. C. , Chung, D. J. , Avogadri, F. , Fink, M. J. et al., Human Langerhans cells use an IL‐15R‐α/IL‐15/pSTAT5‐dependent mechanism to break T‐cell tolerance against the self‐differentiation tumor antigen WT1. Blood 2012. 119: 5182–5190. 22510877 10.1182/blood-2011-09-382200 PMC3369609

[28] Carpentier, S. , Vu Manh, T.‐P. , Chelbi, R. , Henri, S. , Malissen, B. , Haniffa, M. , Ginhoux, F. et al., Comparative genomics analysis of mononuclear phagocyte subsets confirms homology between lymphoid tissue‐resident and dermal XCR1(+) DCs in mouse and human and distinguishes them from Langerhans cells. J. Immunol. Methods 2016. 432: 35–49. 26966045 10.1016/j.jim.2016.02.023 PMC4859332

[29] Stoitzner, P. , Romani, N. , McLellan, A. D. , Tripp, C. H. and Ebner, S. , Isolation of skin dendritic cells from mouse and man. Methods Mol Biol. 2010. 595: 235–248. 19941117 10.1007/978-1-60761-421-0_16

[30] Candi, E. , Schmidt, R. and Melino, G. , The cornified envelope: a model of cell death in the skin. Nat. Rev. Mol. Cell Biol. 2005. 6: 328–340. 15803139 10.1038/nrm1619

[31] Eckert, R. L. , Crish, J. F. and Robinson, N. A. , The epidermal keratinocyte as a model for the study of gene regulation and cell differentiation. Physiol. Rev. 1997. 77: 397–424. 9114819 10.1152/physrev.1997.77.2.397

[32] Banchereau, J. and Steinman, R. M. , Dendritic cells and the control of immunity. Nature 1998. 392: 245–252. 9521319 10.1038/32588

[33] Yao, C. , Zurawski, S. M. , Jarrett, E. S. , Chicoine, B. , Crabtree, J. , Peterson, E. J. , Zurawski, G. et al., Skin dendritic cells induce follicular helper T cells and protective humoral immune responses. J. Allergy Clin. Immunol. 2015. 136: 1387–1397.e7. 25962902 10.1016/j.jaci.2015.04.001 PMC4639468

[34] Merad, M. , Ginhoux, F. and Collin, M. , Origin, homeostasis and function of Langerhans cells and other langerin‐expressing dendritic cells. Nat. Rev. Immunol. 2008. 8: 935–947. 19029989 10.1038/nri2455

[35] Bigley, V. , McGovern, N. , Milne, P. , Dickinson, R. , Pagan, S. , Cookson, S. , Haniffa, M. et al., Langerin‐expressing dendritic cells in human tissues are related to CD1c+ dendritic cells and distinct from Langerhans cells and CD141high XCR1+ dendritic cells. J. Leukoc. Biol. 2015. 97: 627–634. 25516751 10.1189/jlb.1HI0714-351R PMC4370053

[36] Mc Dermott, R. , Ziylan, U. , Spehner, D. , Bausinger, H. , Lipsker, D. , Mommaas, M. , Cazenave, J.‐P. et al., Birbeck granules are subdomains of endosomal recycling compartment in human epidermal langerhans cells, which form where langerin accumulates Pfeffer SR, ed. Mol. Biol. Cell. 2002. 13: 317–335. 11809842 10.1091/mbc.01-06-0300 PMC65091

[37] Malissen, B. , Tamoutounour, S. and Henri, S. , The origins and functions of dendritic cells and macrophages in the skin. Nat. Rev. Immunol. 2014. 14: 417–428. 24854591 10.1038/nri3683

[38] Nestle, F. O. , Conrad, C. , Tun‐Kyi, A. , Homey, B. , Gombert, M. , Boyman, O. , Burg, G. et al., Plasmacytoid predendritic cells initiate psoriasis through interferon‐α production. J. Exp. Med. 2005. 202: 135–143. 15998792 10.1084/jem.20050500 PMC2212894

[39] Debes, G. F. and McGettigan, S. E. , Skin‐Associated B Cells in Health and Inflammation. J. Immunol. 2019. 202: 1659–1666. 30833422 10.4049/jimmunol.1801211 PMC6402607

[40] Wade, C. G. , Rhyne, R. H. , Woodruff, W. H. , Bloch, D. P. and Bartholomew, J. C. , Spectra of cells in flow cytometry using a vidicon detector. J. Histochem. Cytochem. 1979. 27: 1049–1052. 110874 10.1177/27.6.110874

[41] Gauci, M. R. , Vesey, G. , Narai, J. , Veal, D. , Williams, K. L. and Piper, J. A. , Observation of single‐cell fluorescence spectra in laser flow cytometry. Cytometry 1996. 25: 388–393. 8946147 10.1002/(SICI)1097-0320(19961201)25:4<388::AID-CYTO11>3.0.CO;2-R

[42] Nolan, J. P. and Condello, D. , Spectral Flow Cytometry. Curr. Protoc. Cytom. 2013. 63: 1.27.1–1.27.13. 10.1002/0471142956.cy0127s63 PMC3556726 23292705

[43] Chambers, E. S. and Vukmanovic‐Stejic, M. , Skin barrier immunity and ageing. Immunology 2020. 160: 116–125. 31709535 10.1111/imm.13152 PMC7218662

[44] Lang, N. P. , Berglundh, T. , Giannobile, W V. and Sanz, M. , Lindhe's Clinical Periodontology and Implant Dentistry. In: Lindhe's Clinical Periodontology and Implant Dentistry. 7th ed. WILEY Blackwell, 2022.

[45] Wilensky, A. , Mizraji, G. , Tabib, Y. , Sharawi, H. and Hovav, A.‐H. , Analysis of leukocytes in oral mucosal tissues. In: Inflammation. Methods in Molecular Biology. Humana Press, New York, NY, 2017, pp. 267–278. 10.1007/978-1-4939-6786-5_18. 28063050

[46] Arizon, M. , Nudel, I. , Segev, H. , Mizraji, G. , Elnekave, M. , Furmanov, K. , Eli‐Berchoer, L. et al., Langerhans cells down‐regulate inflammation‐driven alveolar bone loss. Proc. Natl. Acad. Sci. 2012. 109: 7043–7048. 22509018 10.1073/pnas.1116770109 PMC3344981

[47] Heyman, O. , Horev, Y. , Koren, N. , Barel, O. , Aizenbud, I. , Aizenbud, Y. , Brandwein, M. et al., Niche specific microbiota‐dependent and independent bone loss around dental implants and teeth. J. Dent. Res. 2020. 99: 1092–1101. 32413268 10.1177/0022034520920577

[48] Iwasaki, A. and Medzhitov, R. , Control of adaptive immunity by the innate immune system. Nat. Immunol. 2015. 16: 343–353. 25789684 10.1038/ni.3123 PMC4507498

[49] Patel, A. A. , Ginhoux, F. and Yona, S. , Monocytes, macrophages, dendritic cells and neutrophils: an update on lifespan kinetics in health and disease. Immunology 2021. 163: 250–261. 33555612 10.1111/imm.13320 PMC8207393

[50] Heath, W. R. and Carbone, F. R. , Dendritic cell subsets in primary and secondary T cell responses at body surfaces. Nat. Immunol. 2009. 10: 1237–1244. 19915624 10.1038/ni.1822

[51] Hajishengallis, G. , Periodontitis: from microbial immune subversion to systemic inflammation. Nat. Rev. Immunol. 2015. 15: 30–44. 25534621 10.1038/nri3785 PMC4276050

[52] Heidkamp, G. F. , Sander, J. , Lehmann, C. H. K. , Heger, L. , Eissing, N. , Baranska, A. , Lu hr, J. J. et al., Human lymphoid organ dendritic cell identity is predominantly dictated by ontogeny, not tissue microenvironment. Sci. Immunol. 2016. 1: eaai7677–eaai7677. 28783692 10.1126/sciimmunol.aai7677

[53] Capucha, T. , Mizraji, G. , Segev, H. , Blecher‐Gonen, R. , Winter, D. , Khalaileh, A. , Tabib, Y. et al., Distinct murine mucosal Langerhans cell subsets develop from pre‐dendritic cells and monocytes. Immunity 2015. 43: 369–381. 26231115 10.1016/j.immuni.2015.06.017

[54] Sharawi, H. , Heyman, O. , Mizraji, G. , Horev, Y. , Laviv, A. , Shapira, L. , Yona, S. et al., The prevalence of gingival dendritic cell subsets in periodontal patients. J. Dent. Res. 2021. 100: 1330–1336. 33899566 10.1177/00220345211004864

[55] Mörbe, U. M. , Jørgensen, P. B. , Fenton, T. M. , von Burg, N. , Riis, L. B. , Spencer, J. and Agace, W. W. , Human gut‐associated lymphoid tissues (GALT); diversity, structure, and function. Mucosal Immunol. 2021. 14: 793–802. 33753873 10.1038/s41385-021-00389-4

[56] Fenton, T. M. , Jørgensen, P. B. , Niss, K. , Rubin, S. J. S. , Mörbe, U. M. , Riis, L. B. , Da Silva, C. et al., Immune profiling of human gut‐associated lymphoid tissue identifies a role for isolated lymphoid follicles in priming of region‐specific immunity. Immunity 2020. 52: 557–570.e6. 32160523 10.1016/j.immuni.2020.02.001 PMC7155934

[57] Jørgensen, P. B. , Fenton, T. M. , Mörbe, U. M. , Riis, L. B. , Jakobsen, H. L. , Nielsen, O. H. and Agace, W. W. , Identification, isolation and analysis of human gut‐associated lymphoid tissues. Nat. Protoc. 2021. 16: 2051–2067. 33619391 10.1038/s41596-020-00482-1

[58] Joeris, T. , Müller‐Luda, K. , Agace, W. W. and Mowat, A. M. , Diversity and functions of intestinal mononuclear phagocytes. Mucosal Immunol. 2017. 10: 845–864. 28378807 10.1038/mi.2017.22

[59] Persson, E. , Uronen‐Hansson, H. , Semmrich, M. , Rivollier, A. , Hägerbrand, K. , Marsal, J. , Gudjonsson, S. et al., IRF4 transcription‐factor‐dependent CD103+CD11b+ dendritic cells drive mucosal T helper 17 Cell differentiation. Immunity 2013. 38: 958–969. 23664832 10.1016/j.immuni.2013.03.009

[60] Flores‐Langarica, A. , Müller Luda, K. , Persson, E. K. , Cook, C. N. , Bobat, S. , Marshall, J. L. , Dahlgren, M. W. et al., CD103+CD11b+ mucosal classical dendritic cells initiate long‐term switched antibody responses to flagellin. Mucosal Immunol. 2018. 11: 681–692. 29346347 10.1038/mi.2017.105 PMC5912514

[61] Mayer, J. U. , Demiri, M. , Agace, W. W. , MacDonald, A. S. , Svensson‐Frej, M. and Milling, S. W. , Different populations of CD11b+ dendritic cells drive Th2 responses in the small intestine and colon. Nat. Commun. 2017. 8: 15820. 28598427 10.1038/ncomms15820 PMC5472728

[62] Luda, K. M. , Joeris, T. , Persson, E. K. , Rivollier, A. , Demiri, M. , Sitnik, K. M. , Pool, L. et al., IRF8 Transcription‐Factor‐Dependent Classical Dendritic Cells Are Essential for Intestinal T Cell Homeostasis. Immunity 2016. 44: 860–874. 27067057 10.1016/j.immuni.2016.02.008

[63] Joeris, T. , Gomez‐Casado, C. , Holmkvist, P. , Tavernier, S. J. , Silva‐Sanchez, A. , Klotz, L. , Randall, T. D. et al., Intestinal cDC1 drive cross‐tolerance to epithelial‐derived antigen via induction of FoxP3 + CD8 + T regs. Sci. Immunol. 2021. 6: eabd3774. 34088744 10.1126/sciimmunol.abd3774

[64] Guendel, F. , Kofoed‐Branzk, M. , Gronke, K. , Tizian, C. , Witkowski, M. , Cheng, H.‐W. , Heinz, G. A. et al., Group 3 innate lymphoid cells program a distinct subset of IL‐22BP‐producing dendritic cells demarcating solitary intestinal lymphoid tissues. Immunity 2020. 53: 1015–1032e8. 33207209 10.1016/j.immuni.2020.10.012

[65] Da Silva, C. , Wagner, C. , Bonnardel, J. , Gorvel, J.‐P. and Lelouard, H. , The Peyer's patch mononuclear phagocyte system at steady state and during infection. Front. Immunol. 2017. 8: 1254. 29038658 10.3389/fimmu.2017.01254 PMC5630697

[66] Chen, D. S. and Mellman, I. , Elements of cancer immunity and the cancer–immune set point. Nature 2017. 541: 321–330. 28102259 10.1038/nature21349

[67] van de Ven, R. , van den Hout, M. , Lindenberg, J. J. , Sluijter, B. J. R. , van Leeuwen P a, M. , Lougheed, S. M. , Meijer, S. et al., Characterization of four conventional dendritic cell subsets in human skin‐draining lymph nodes in relation to T‐cell activation. Blood 2011. 118: 2502–2510. 21750314 10.1182/blood-2011-03-344838

[68] van Pul, K. M. , Fransen, M. F. , van de Ven, R. and de Gruijl, T. D. , Immunotherapy goes local: the central role of lymph nodes in driving tumor infiltration and efficacy. Front. Immunol. 2021. 12: 643291. 33732264 10.3389/fimmu.2021.643291 PMC7956978

[69] Borst, J. , Ahrends, T. , Bąbała, N. , Melief, C. J. M. and Kastenmüller, W. , CD4+ T cell help in cancer immunology and immunotherapy. Nat. Rev. Immunol. 2018. 18: 635–647. 30057419 10.1038/s41577-018-0044-0

[70] Wu, T. D. , Madireddi, S. , de Almeida, P. E. , Banchereau, R. , Chen, Y.‐J. J. , Chitre, A. S. , Chiang, E. Y. et al., Peripheral T cell expansion predicts tumour infiltration and clinical response. Nature 2020. 579: 274–278. 32103181 10.1038/s41586-020-2056-8

[71] Moussion, C. and Mellman, I. , The Dendritic Cell Strikes Back. Immunity 2018. 49: 997–999. 30566889 10.1016/j.immuni.2018.12.007

[72] Mayoux, M. , Roller, A. , Pulko, V. , Sammicheli, S. , Chen, S. , Sum, E. , Jost, C. et al., Dendritic cells dictate responses to PD‐L1 blockade cancer immunotherapy. Sci. Transl. Med. 2020. 12: eaav7431. 32161104 10.1126/scitranslmed.aav7431

[73] Yost, K. E. , Satpathy, A. T. , Wells, D. K. , Qi, Y. , Wang, C. , Kageyama, R. , McNamara, K. L. et al., Clonal replacement of tumor‐specific T cells following PD‐1 blockade. Nat. Med. 2019. 25: 1251–1259. 31359002 10.1038/s41591-019-0522-3 PMC6689255

[74] Oh, S. A. , Wu, D.‐C. , Cheung, J. , Navarro, A. , Xiong, H. , Cubas, R. , Totpal, K. et al., PD‐L1 expression by dendritic cells is a key regulator of T‐cell immunity in cancer. Nat. Cancer. 2020. 1: 681–691. 35122038 10.1038/s43018-020-0075-x

[75] Spranger, S. , Bao, R. and Gajewski, T. F. , Melanoma‐intrinsic β‐catenin signalling prevents anti‐tumour immunity. Nature 2015. 523: 231–235. 25970248 10.1038/nature14404

[76] Spranger, S. , Dai, D. , Horton, B. and Gajewski, T. F. , Tumor‐residing Batf3 dendritic cells are required for effector T cell trafficking and adoptive T cell therapy. Cancer Cell 2017. 31: 711–723.e4. 28486109 10.1016/j.ccell.2017.04.003 PMC5650691

[77] Mulder, W. M. C. , Koenen, H. , van de Muysenberg, A. J. C. , Bloemena, E. , Wagsfaff, J. and Scheper, R. J. , Reduced expression of distinct T‐cell CD molecules by collagenase/DNase treatment. Cancer Immunol. Immunother. 1994. 38: 253–258. 8168120 10.1007/BF01533516 PMC11038093

[78] Ginhoux, F. , Guilliams, M. and Merad, M. , Expanding dendritic cell nomenclature in the single‐cell era. Nat. Rev. Immunol. 2022. 22: 67–68. 35027741 10.1038/s41577-022-00675-7

[79] Poulin, L. F. , Salio, M. , Griessinger, E. , Anjos‐Afonso, F. , Craciun, L. , Chen, J.‐L. , Keller, A. M. et al., Characterization of human DNGR‐1+ BDCA3+ leukocytes as putative equivalents of mouse CD8alpha+ dendritic cells. J. Exp. Med. 2010. 207: 1261–1271. 20479117 10.1084/jem.20092618 PMC2882845

[80] Segura, E. , Durand, M. and Amigorena, S. , Similar antigen cross‐presentation capacity and phagocytic functions in all freshly isolated human lymphoid organ–resident dendritic cells. J. Exp. Med. 2013. 210: 1035–1047. 23569327 10.1084/jem.20121103 PMC3646495

[81] Cytlak, U. , Resteu, A. , Pagan, S. , Green, K. , Milne, P. , Maisuria, S. , McDonald, D. et al., Differential IRF8 transcription factor requirement defines two pathways of dendritic cell development in humans. Immunity 2020. 53: 353–370.e8. 32735845 10.1016/j.immuni.2020.07.003 PMC7447982

[82] van den Hout, M. , Koster, B. D. , Sluijter, B. J. R. , Molenkamp, B. G. , van de Ven, R. , van den Eertwegh, A. J. M. , Scheper, R. J. et al., Melanoma sequentially suppresses different DC subsets in the sentinel lymph node, affecting disease spread and recurrence. Cancer Immunol. Res. 2017. 5: 969–977. 28935649 10.1158/2326-6066.CIR-17-0110

[83] Binnewies, M. , Mujal, A. M. , Pollack, J. L. , Combes, A. J. , Hardison, E. A. , Barry, K. C. , Tsui, J. et al., Unleashing type‐2 dendritic cells to drive protective antitumor CD4+ T cell immunity. Cell 2019. 177: 556–571.e16. 30955881 10.1016/j.cell.2019.02.005 PMC6954108

[84] Prokopi, A. , Tripp, C. H. , Tummers, B. , Hornsteiner, F. , Spoeck, S. , Crawford, J. C. , Clements, D. R. et al., Skin dendritic cells in melanoma are key for successful checkpoint blockade therapy. J. Immunother. Cancer. 2021. 9: e000832. 33408092 10.1136/jitc-2020-000832 PMC7789456

[85] Rotman, J. , Heeren, A. M. , Gassama, A. A. , Lougheed, S. M. , Pocorni, N. , Stam, A. G. M. , Bleeker, M. C. G. et al., Adenocarcinoma of the uterine cervix shows impaired recruitment of cDC1 and CD8+ T cells and elevated β‐catenin activation compared with squamous cell carcinoma. Clin. Cancer Res. 2020. 26: 3791–3802. 32220890 10.1158/1078-0432.CCR-19-3826

[86] Luke, J. J. , Bao, R. , Sweis, R. F. , Spranger, S. and Gajewski, T. F. , WNT/β‐catenin pathway activation correlates with immune exclusion across human cancers. Clin. Cancer Res. 2019. 25: 3074–3083. 30635339 10.1158/1078-0432.CCR-18-1942 PMC6522301

[87] Garris, C. S. , Arlauckas, S. P. , Kohler, R. H. , Trefny, M. P. , Garren, S. , Piot, C. , Engblom, C. et al., Successful anti‐PD‐1 cancer immunotherapy requires T cell‐dendritic cell crosstalk involving the cytokines IFN‐γ and IL‐12. Immunity 2018. 49: 1148–1161.e7. 30552023 10.1016/j.immuni.2018.09.024 PMC6301092

[88] Duraiswamy, J. , Turrini, R. , Minasyan, A. , Barras, D. , Crespo, I. , Grimm, A. J. , Casado, J. et al., Myeloid antigen‐presenting cell niches sustain antitumor T cells and license PD‐1 blockade via CD28 costimulation. Cancer Cell 2021. 39: 1623–1642.e20. 34739845 10.1016/j.ccell.2021.10.008 PMC8861565

[89] Santegoets, S. , Gibbs, S. , Kroeze, K. , van de Ven, R. , Scheper, R. J. , Borrebaeck, C. A. , de Gruijl, T. D. et al., Transcriptional profiling of human skin‐resident Langerhans cells and CD1a+ dermal dendritic cells: differential activation states suggest distinct functions. J. Leukoc. Biol. 2008. 84: 143–151. 18436579 10.1189/jlb.1107750

[90] Lundberg, K. , Albrekt, A.‐S. , Nelissen, I. , Santegoets, S. , de Gruijl, T. D. , Gibbs, S. and Lindstedt, M. , Transcriptional profiling of human dendritic cell populations and models ‐ unique profiles of in vitro dendritic cells and implications on functionality and applicability Appel S, ed. PLoS One. 2013. 8: e52875. 23341914 10.1371/journal.pone.0052875 PMC3544800

[91] van de Ven, R. , Lindenberg, J. J. , Oosterhoff, D. and de Gruijl, T. D. , Dendritic cell plasticity in tumor‐conditioned skin: CD14+ cells at the cross‐roads of immune activation and suppression. Front. Immunol. 2013. 4: 403. 24324467 10.3389/fimmu.2013.00403 PMC3839226

[92] Lindenberg, J. J. , van de Ven, R. , Lougheed, S. M. , Zomer, A. , Santegoets, S. J. , Griffioen, A. W. , Hooijberg, E. et al., Functional characterization of a STAT3‐dependent dendritic cell‐derived CD14 + cell population arising upon IL‐10‐driven maturation. Oncoimmunology 2013. 2: e23837. 23734330 10.4161/onci.23837 PMC3654600

[93] Santegoets, S. J. , Duurland, C. L. , Jordanova, E. J. , van Ham, V. J. , Ehsan, I. , Loof, N. M. , Narang, V. et al., CD163+ cytokine‐producing cDC2 stimulate intratumoral type 1 T cell responses in HPV16‐induced oropharyngeal cancer. J. Immunother. cancer. 2020. 8: 1–15. 10.1136/jitc-2020-001053 PMC7418847 32771994

[94] Mulder, K. , Patel, A. A. , Kong, W. T. , Piot, C. , Halitzki, E. , Dunsmore, G. , Khalilnezhad, S. et al., Cross‐tissue single‐cell landscape of human monocytes and macrophages in health and disease. Immunity 2021. 54: 1883–1900.e5. 34331874 10.1016/j.immuni.2021.07.007

[95] Oosterhoff, D. , Lougheed, S. , van de Ven, R. , Lindenberg, J. , van Cruijsen, H. , Hiddingh, L. , Kroon, J. et al., Tumor‐mediated inhibition of human dendritic cell differentiation and function is consistently counteracted by combined p38 MAPK and STAT3 inhibition. Oncoimmunology 2012. 1: 649–658. 22934257 10.4161/onci.20365 PMC3429569

[96] López González, M. , Oosterhoff, D. , Lindenberg, J. J. , Milenova, I. , Lougheed, S. M. , Martiáñez, T. , Dekker, H. et al., Constitutively active GSK3β as a means to bolster dendritic cell functionality in the face of tumour‐mediated immune suppression. Oncoimmunology. 2019. 8: e1631119 31646076 10.1080/2162402X.2019.1631119 PMC6791458

[97] Sharma, M. D. , Rodriguez, P. C. , Koehn, B. H. , Baban, B. , Cui, Y. , Guo, G. , Shimoda, M. et al., Activation of p53 in immature myeloid precursor cells controls differentiation into Ly6c+CD103+ monocytic antigen‐presenting cells in tumors. Immunity 2018. 48: 91–106.e6. 29343444 10.1016/j.immuni.2017.12.014 PMC6005382

[98] Schetters, S. T. T. , Rodriguez, E. , Kruijssen, L. J. W. , Crommentuijn, M. H. W. , Boon, L. , van den Bossche, J. , den Haan, J. M. M. et al., Monocyte‐derived APCs are central to the response of PD1 checkpoint blockade and provide a therapeutic target for combination therapy. J. Immunother. Cancer. 2020. 8: e000588. 32690667 10.1136/jitc-2020-000588 PMC7371367

[99] van Ee, T. , Van Acker, H. , van Oorschot, T. , Van Tendeloo, V. , Smits, E. , Bakdash, G. , Schreibelt, G. et al., BDCA1+CD14+ immunosuppressive cells in cancer, a potential target? Vaccines 2018. 6: 65. 30235890 10.3390/vaccines6030065 PMC6161086

[100] Collin, M. and Bigley, V. , Human dendritic cell subsets: an update. Immunology 2018. 154: 3–20. 29313948 10.1111/imm.12888 PMC5904714

[101] Segura, E. , Valladeau‐Guilemond, J. , Donnadieu, M.‐H. , Sastre‐Garau, X. , Soumelis, V. and Amigorena, S. , Characterization of resident and migratory dendritic cells in human lymph nodes. J. Exp. Med. 2012. 209: 653–660. 22430490 10.1084/jem.20111457 PMC3328358

[102] Sluijter, B. J. R. , van den Hout, M. , Koster, B. D. , van Leeuwen, P. A. M. , Schneiders, F. L. , van de Ven, R. , Molenkamp, B. G. et al., Arming the melanoma sentinel lymph node through local administration of CpG‐B and GM‐CSF: recruitment and activation of BDCA3/CD141+ dendritic cells and enhanced cross‐presentation. Cancer Immunol. Res. 2015. 3: 495–505. 25633713 10.1158/2326-6066.CIR-14-0165

[103] van Krimpen, A. , Gerretsen, V. I. V. , Mulder, E. , van Gulijk, M. , van den Bosch, T. P. P. , von der Thüsen, J. , Grünhagen, D. J. et al., Immune suppression in the tumor‐draining lymph node corresponds with distant disease recurrence in patients with melanoma. Cancer Cell 2022. 40: 798–799. 35839777 10.1016/j.ccell.2022.06.009

[104] van Pul, K. M. , Vuylsteke, R. , van de Ven, R. , te Velde, E. A. , Rutgers, E. J. T. , van den Tol, P. M. , Stockmann, H. et al., Selectively hampered activation of lymph node‐resident dendritic cells precedes profound T cell suppression and metastatic spread in the breast cancer sentinel lymph node. J. Immunother. Cancer. 2019. 7: 133. 31118093 10.1186/s40425-019-0605-1 PMC6530094

[105] Vuylsteke, R. , van Leeuwen, P. A. M. , Meijer, S. , Wijnands, P. , Statius Muller, M. G. , Busch, D. H. , Scheper, R. J. et al., Sampling tumor‐draining lymph nodes for phenotypic and functional analysis of dendritic cells and T cells. Am. J. Pathol. 2002. 161: 19–26. 12107085 10.1016/S0002-9440(10)64152-1 PMC1850698

[106] Elliott, B. , Cook, M. G. , John, R. J. , Powell, B. , Pandha, H. and Dalgleish, A. G. , Successful live cell harvest from bisected sentinel lymph nodes research report. J. Immunol. Methods. 2004. 291: 71–78. 15345306 10.1016/j.jim.2004.04.025

[107] van Pul, K. M. , Vuylsteke, R. , Bril, H. , Stockmann, H. and de Gruijl, T. D. , Feasibility of flowcytometric quantitation of immune effector cell subsets in the sentinel lymph node of the breast after cryopreservation. J. Immunol. Methods. 2012. 375: 189–195. 22062586 10.1016/j.jim.2011.10.011

[108] van Pul, K. M. , Vuylsteke, R. , de Beijer, M. T. A. , van de Ven, R. , van den Tol, M. P. , Stockmann, H. and de Gruijl, T. D. , Breast cancer‐induced immune suppression in the sentinel lymph node is effectively countered by CpG‐B in conjunction with inhibition of the JAK2/STAT3 pathway. J. Immunother. Cancer. 2020. 8: e000761. 33046620 10.1136/jitc-2020-000761 PMC7552844

[109] Fransen, M. F. , Arens, R. and Melief, C. J. M. , Local targets for immune therapy to cancer: tumor draining lymph nodes and tumor microenvironment. Int. J. Cancer. 2013. 132: 1971–1976. 22858832 10.1002/ijc.27755

[110] Chamoto, K. , Chowdhury, P. S. , Kumar, A. , Sonomura, K. , Matsuda, F. , Fagarasan, S. and Honjo, T. , Mitochondrial activation chemicals synergize with surface receptor PD‐1 blockade for T cell‐dependent antitumor activity. Proc. Natl. Acad. Sci. 2017. 114: E761–E770. 28096382 10.1073/pnas.1620433114 PMC5293087

[111] Koster, B. D. , van den Hout, M. , Sluijter, B. J. R. , Molenkamp, B. G. , Vuylsteke, R. , Baars, A. , van Leeuwen, P. A. M. et al., Local adjuvant treatment with low‐dose CpG‐B offers durable protection against disease recurrence in clinical stage I–II melanoma: data from two randomized phase II trials. Clin. Cancer Res. 2017. 23: 5679–5686. 28972083 10.1158/1078-0432.CCR-17-0944

[112] Fransen, M. F. , Schoonderwoerd, M. , Knopf, P. , Camps, M. G. M. , Hawinkels, L. , Kneilling, M. , van Hall, T. et al., Tumor‐draining lymph nodes are pivotal in PD‐1/PD‐L1 checkpoint therapy. JCI Insight 2018. 3: 124507. 30518694 10.1172/jci.insight.124507 PMC6328025

[113] Rotman, J. , Koster, B. D. , Jordanova, E. S. , Heeren, A. M. and de Gruijl, T. D. , Unlocking the therapeutic potential of primary tumor‐draining lymph nodes. Cancer Immunol. Immunother. 2019. 68: 1681–1688. 30944963 10.1007/s00262-019-02330-y PMC6805797

[114] Koster, B. D. , de Jong, T. D. , van den Hout, M. , Sluijter, B. J. R. , Vuylsteke, R. , Molenkamp, B. G. , Vosslamber, S. et al., In the mix: the potential benefits of adding GM‐CSF to CpG‐B in the local treatment of patients with early‐stage melanoma. Oncoimmunology 2020. 9: 1708066. 32002303 10.1080/2162402X.2019.1708066 PMC6959435

[115] Dammeijer, F. , van Gulijk, M. , Mulder, E. E. , Lukkes, M. , Klaase, L. , van den Bosch, T. , van Nimwegen, M. et al., The PD‐1/PD‐L1‐checkpoint restrains T cell Immunity in tumor‐draining lymph nodes. Cancer Cell 2020. 38: 685–700.e8. 33007259 10.1016/j.ccell.2020.09.001

[116] de Mingo Pulido, Á. , Gardner, A. , Hiebler, S. , Soliman, H. , Rugo, H. S. , Krummel, M. F. , Coussens, L. M. et al., TIM‐3 regulates CD103+ dendritic cell function and response to chemotherapy in breast cancer. Cancer Cell 2018. 33: 60–74.e6. 29316433 10.1016/j.ccell.2017.11.019 PMC5764109

[117] de Mingo Pulido, Á. , Hänggi, K. , Celias, D. P. , Gardner, A. , Li, J. , Batista‐Bittencourt, B. , Mohamed, E. et al., The inhibitory receptor TIM‐3 limits activation of the cGAS‐STING pathway in intra‐tumoral dendritic cells by suppressing extracellular DNA uptake. Immunity. 2021. 54: 1154–1167.e7. 33979578 10.1016/j.immuni.2021.04.019 PMC8192496

[118] Gardner, A. , De Mingo Pulido, Á. , Hänggi, K. , Bazargan, S. , Onimus, A. , Kasprzak, A. , Conejo‐Garcia, J. R. et al., TIM‐3 blockade enhances IL‐12‐dependent antitumor immunity by promoting CD8 + T cell and XCR1 + dendritic cell spatial co‐localization. J. Immunother. Cancer. 2022. 10: e003571. 34987021 10.1136/jitc-2021-003571 PMC8734033

